# A Placebo-controlled Trial of Percutaneous Coronary Intervention for Stable Angina

**DOI:** 10.1056/NEJMoa2310610

**Published:** 2023-11-11

**Authors:** Christopher A Rajkumar, Michael J Foley, Fiyyaz Ahmed-Jushuf, Alexandra N Nowbar, Florentina Simader, John R Davies, Peter D O’Kane, Peter Haworth, Helen Routledge, Tushar Kotecha, Reto Gamma, Gerald Clesham, Rupert Williams, Jehangir Din, Sukhjinder S Nijjer, Nick Curzen, Neil Ruparelia, Manas Sinha, Jason N Dungu, Sashiananthan Ganesananthan, Ramzi Khamis, Lal Mughal, Tim Kinnaird, Ricardo Petraco, James C Spratt, Sayan Sen, Joban Sehmi, David J Collier, Afzal Sohaib, Thomas R Keeble, Graham D Cole, James P Howard, Darrel P Francis, Matthew J Shun-Shin, Rasha Al-Lamee

**Affiliations:** 1 Imperial College London; 2 Imperial College Healthcare NHS Trust; 3 Barking Havering and Redbridge University Hospitals NHS Trust; 4 Essex Cardiothoracic Centre, Mid and South Essex NHS Foundation Trust; 5 Anglia Ruskin University; 6 University Hospitals of Dorset NHS Foundation Trust; 7 Portsmouth Hospitals University NHS Trust; 8 Worcestershire Acute Hospitals NHS Trust; 9 Royal Free London NHS Foundation Trust; 10 St George’s University Hospitals NHS Foundation Trust; 11 University Hospital Southampton NHS Foundation Trust; 12 University of Southampton; 13 Royal Berkshire NHS Foundation Trust; 14 Salisbury NHS Foundation Trust; 15 Cardiff and Vale University Health Board; 16 Keele University; 17 Buckinghamshire Healthcare NHS Trust; 18 St George’s, University of London; 19 West Hertfordshire Hospitals NHS Trust; 20 Queen Mary University of London; 21 Barts Health NHS Trust

## Abstract

**Background:**

Percutaneous coronary intervention (PCI) is frequently performed to improve symptoms of stable angina. Whether PCI without background antianginal medication relieves angina beyond placebo remains unknown.

**Methods:**

We conducted a randomized, double-blind, placebo-controlled trial of PCI in patients with stable angina. Patients stopped all antianginal medications and underwent a 2-week pre-randomization symptom assessment. Patients were randomized 1:1 to PCI or placebo and were followed for 12-weeks. The primary end point was the angina symptom score calculated daily based on the number of angina episodes, antianginal medication restarts, unblinding for intolerable angina, acute coronary syndrome, and death.

**Results:**

A total of 301 patients were randomized, with 151 patients assigned to receive PCI and 150 to placebo. The mean age of patients was 64±9 years and 79% were male. Ischemia was present in one cardiac territory in 242 patients (80%), two territories in 52 patients (17%) and three territories in 7 patients (2%). The median fractional flow reserve was 0.63 (interquartile range, 0.49 to 0.75) and the median instantaneous wave-free ratio was 0.78 (interquartile range, 0.55 to 0.87). At 12-week follow-up, the angina symptom score was 2.9 for patients assigned to PCI and 5.6 for patients assigned to placebo (odds ratio 2.21; 95% confidence interval, 1.41 to 3.47, p<0.001). One patient in the placebo arm had intolerable angina. Acute coronary syndromes occurred in 4 patients in the PCI group and 6 in the placebo group.

**Conclusions:**

Among patients with stable angina on little or no antianginal medication and objective evidence of ischemia, PCI improved the angina symptom score compared to placebo. (Funded by NIHR Imperial Biomedical Research Centre, Medical Research Council, NIHR, British Heart Foundation, Philips, Coronary Flow Trust; ORBITA-2 ClinicalTrials.gov: NCT03742050)

## Introduction

Angina relief is the primary reason that patients with stable coronary artery disease undergo percutaneous coronary intervention (PCI).^1–3^ The evidence that PCI reduces angina comes from unblinded clinical trials^1,4–6^ where the overall effect of PCI on symptoms is due to both physical and placebo components.^7^ The size of the physical component, calculated with placebo control, is essential knowledge in clinical decision-making, especially for procedures with non-negligible risk and cost.^8^

ORBITA (Objective Randomized Blinded Investigation with optimal medical Therapy of Angioplasty in stable angina), a placebo-controlled trial of PCI that mandated the use of guideline-directed antianginal medications, found no effect of PCI on exercise time.^9^ However, it is possible that the absence of a difference between PCI and placebo was attributable to the high number of background antianginal medications. Intensive antianginal medical therapy can be difficult to achieve in clinical practice, in part due to side-effects and non-adherence, and there are instances when patients may prefer PCI to medication escalation.

The ORBITA-2 trial was designed to determine the effect of PCI compared to placebo without background antianginal medication.

## Methods

### Trial Design And Oversight

The ORBITA-2 trial was an investigator-initiated multicenter, double-blind, randomized, placebo-controlled trial performed at 14 sites in the United Kingdom. The full protocol^10^ has been published and is available online at NEJM.org. The trial was approved by the London Central Research Ethics committee (reference 18/LO/1203). All patients provided written informed consent. Study centers and investigators are listed in Table S1 in the Supplementary Appendix (available online with the full text of this article at NEJM.org). The trial steering committee and an independent data safety monitoring board supervised the trial conduct (a list of members is provided in the Supplementary Appendix). The trial was designed by the last author and MSS analyzed the data. The first draft of the manuscript was written by CAR and RAL. CAR, MSS, DPF and RAL were responsible for the final manuscript. The first and last authors vouch for the data and analysis and had final responsibility for the decision to submit for publication. The sponsor and the funders had no role in the design, collection, analysis, or interpretation of the data, or writing of the report.

### Patients

Patients were eligible if they were considered clinically suitable for PCI by the referring heart team, had angina or an anginal equivalent, anatomical evidence of at least one severe coronary stenosis identified by invasive diagnostic coronary angiography or computed tomography coronary angiography and non-invasive or invasive evidence of ischemia. Additional criteria are provided in the Supplementary Appendix.

### Study Procedures And Randomization

At enrollment, patients ceased antianginal medication. Antihypertensive medications with antianginal properties were replaced with alternatives with no antianginal effects. Medications with antianginal properties required for other clinical indications such as heart failure or atrial fibrillation rate control were continued. Risk reduction medications including dual antiplatelet medication and high-intensity statins were prescribed.

Patients were taught to use a dedicated smartphone application for daily angina symptom reporting. The design, features,^11^ and validation^12^ of the smartphone application are available (Fig. S1). Patients completed the Seattle Angina Questionnaire (SAQ) and the EuroQOL 5 dimensions (EQ-5D-5L). They then entered a 2-week pre-randomization symptom assessment phase and reported daily angina via the smartphone application. Patients had 24-hour access to the trial team and antianginal medications were introduced following a pre-specified protocol. They proceeded to randomization if they reported at least one angina episode during the symptom assessment phase. Asymptomatic patients were withdrawn.

Patients underwent coronary angiography with auditory isolation using over-the-ear headphones with music playing throughout the procedure. Pre-randomization invasive physiological assessments were performed in each vessel with a ≥50% visual stenosis; operators identified the target vessels for PCI. For randomization, evidence of ischemia was required in at least one cardiac territory. Non-ischemic patients were withdrawn.

Eligible patients were administered incremental doses of intravenous benzodiazepines and opiates to achieve a deep level of conscious sedation until they were unresponsive to verbal and tactile stimuli. Patients were then randomized 1:1 to PCI or placebo procedure using computer generated randomization with block size between 8 and 16 and no stratification.

### Blinding And Interventions

For the PCI group, angiographic and physiological complete revascularization of target vessels was mandated, and intravascular imaging encouraged. For multivessel coronary-artery disease, all vessels were treated during the index procedure. The placebo group remained sedated, without any further intervention, for at least 15 minutes after randomization.

There was no transfer of information regarding the study group assignment to blinded recovery staff. All subsequent medical caregivers were blinded to treatment allocation. The operator and research staff present during the randomization procedure had no further patient contact. Each patient and the recovery team underwent a test of blinding prior to discharge. Patients were discharged with standardized discharge documentation and dual antiplatelet medication on the day of randomization unless otherwise clinically indicated. Details of the blinding framework^13^ and testing of its efficacy are available (Table S2).

### Follow-Up

On the day of randomization, all antianginal medications initiated in the pre-randomization period were stopped. Patients entered the 12-week blinded follow-up phase, with continued daily smartphone application symptom reporting. During this period, antianginal medication initiation and uptitration was triggered by patient contact and managed by staff blinded to study group allocation, using an identical protocol to the pre-randomization phase.

At the end of the blinded follow-up period, patients returned for exercise stress test, dobutamine stress echo and symptom and quality of life questionnaires. After test completion, patients and research and medical teams were unblinded. This marked the end of the study. Patients returned to usual clinical care. Any actions or decisions after unblinding did not contribute to study end point data.

### End Points

The primary end point is the angina symptom score: an ordinal clinical outcome scale calculated daily from the number of angina episodes and the units of antianginal medication (Tables S3 and S4). More angina episodes and antianginal medication use led to a higher score. A patient with no angina and no antianginals scored zero. This ordinal scale also incorporated high-level category overrides of unblinding for intolerable angina (requiring unscheduled coronary angiography), acute coronary syndrome (unstable angina or myocardial infarction meeting the fourth universal definition) or death^10^ (Table S4).

Secondary end points included self-reported angina frequency (smartphone application); antianginal medication initiation and uptitration; treadmill exercise time increment; physician-assessed angina severity (Canadian Cardiovascular Society (CCS) class); angina frequency, physical limitation, angina stability, freedom from angina (SAQ); quality of life (SAQ and EQ-5D-5L); and stress echocardiography score.^14^

### Statistical Analysis

The sample size was determined by assuming a standard deviation of 6 angina symptom score units; 284 patients would give 80% power with an alpha of 0.05 to detect a difference of 2 angina symptom score units between the PCI and placebo groups, using a 2-sample t-test. Based on prior experience, the study planned to enroll 396 patients to achieve 284 randomized patients. The trial protocol specified that an analysis of covariance of the ordinal angina symptom score would be performed as the primary analysis. The Statistical Working Group prepared a Statistical Analysis Plan (V1.0, 14^th^ June 2021, 2.2 years prior to data lock; available online at NEJM.org). This analytical plan specified a Bayesian framework with a longitudinal analysis of the primary end point. However, NEJM required that we present the frequentist analysis of covariance to analyze the end points as originally specified in the study protocol. The Bayesian analysis is presented in the Supplementary Appendix.

For the primary end point, if daily symptom data were not available, the last entered value was used as the final follow-up value unless a high category override event occurred. During the COVID-19 pandemic, hospital research visits for exercise stress tests and stress echocardiography were suspended during national restrictions. This did not impact the primary or questionnaire-based end points. A complete case analysis is presented for the primary endpoint. A sensitivity analysis for the treadmill exercise time and stress echocardiography score endpoints using multiple imputation to account for missing data was performed.

The primary end point was analyzed by an ordinal analysis of covariance, which uses a cumulative probability model (also called “cumulative link model”) that does not impose distributional assumptions on the outcome.^15^ Individual components of the primary end point and the ordinal secondary endpoint of CCS class were analyzed using the same ordinal analysis of covariance technique. For freedom from angina, a logistic regression model was used. For other secondary end points, an ordinary least squares model was used. Restricted cubic splines were used to allow for non-linear effects.

There were no pre-specified plans to adjust for multiplicity. Therefore, the results are reported as point estimates and 95% confidence intervals and the widths of the confidence intervals should not be used in place of a hypothesis test. The blinding index for patients and staff at baseline and follow-up were calculated using published methods.^16^ All analyses were conducted using R^17^ using the package rms^18^ for the frequentist analysis, the rmsb^19^ package for Bayesian modeling and the BI package ^20^ for the blinding index.

## Results

### Patients

Between 12th November 2018 and 17th June 2023, 923 patients were assessed for eligibility. Of these, 439 were enrolled into the pre-randomization symptom assessment phase, and 301 were randomized to PCI or placebo (Fig. S2). Baseline characteristics of the randomized patients are described in Table 1. The mean age of patients was 64±9 years and 79% were male. The trial population was representative of patients with stable coronary artery disease in the United Kingdom (Table S5). At enrollment, 290 patients (96%) were CCS class II or III. Cardiovascular risk factor assessment is shown in Table S6. The median number of antianginal agents at enrollment and before protocol-mandated cessation was 1, equivalent to a median of 2 standardized antianginal units.

### Procedural characteristics

Procedural characteristics are described in Table 2. Radial artery access was used in 288 patients (96%). Invasive physiological assessment was performed in a median of 1 vessel per patient. Cardiac territories with ischemia were identified through pre-randomization invasive physiology and pre-enrollment non-invasive functional testing: 242 patients (80%) had one territory, 52 (17%) had two territories and 7 (2%) had three territories.

Images of qualifying coronary lesions from all randomized patients are shown in Figure S3. By quantitative coronary angiography, the mean diameter stenosis was 61.2±17.7%. FFR and iFR were performed in 349 (91.1%) and 352 (91.9%) of 383 target vessels, respectively. In the target vessels, the median FFR was 0.63 (interquartile range, 0.49 to 0.75) and iFR was 0.78 (interquartile range, 0.55 to 0.87). Complete revascularization was achieved in all but two patients. In both, pressure wire pullback and intravascular imaging demonstrated diffuse disease, which was managed conservatively. Post-PCI coronary physiology is reported in Table S7.

### Primary end point

**Figure 1:**
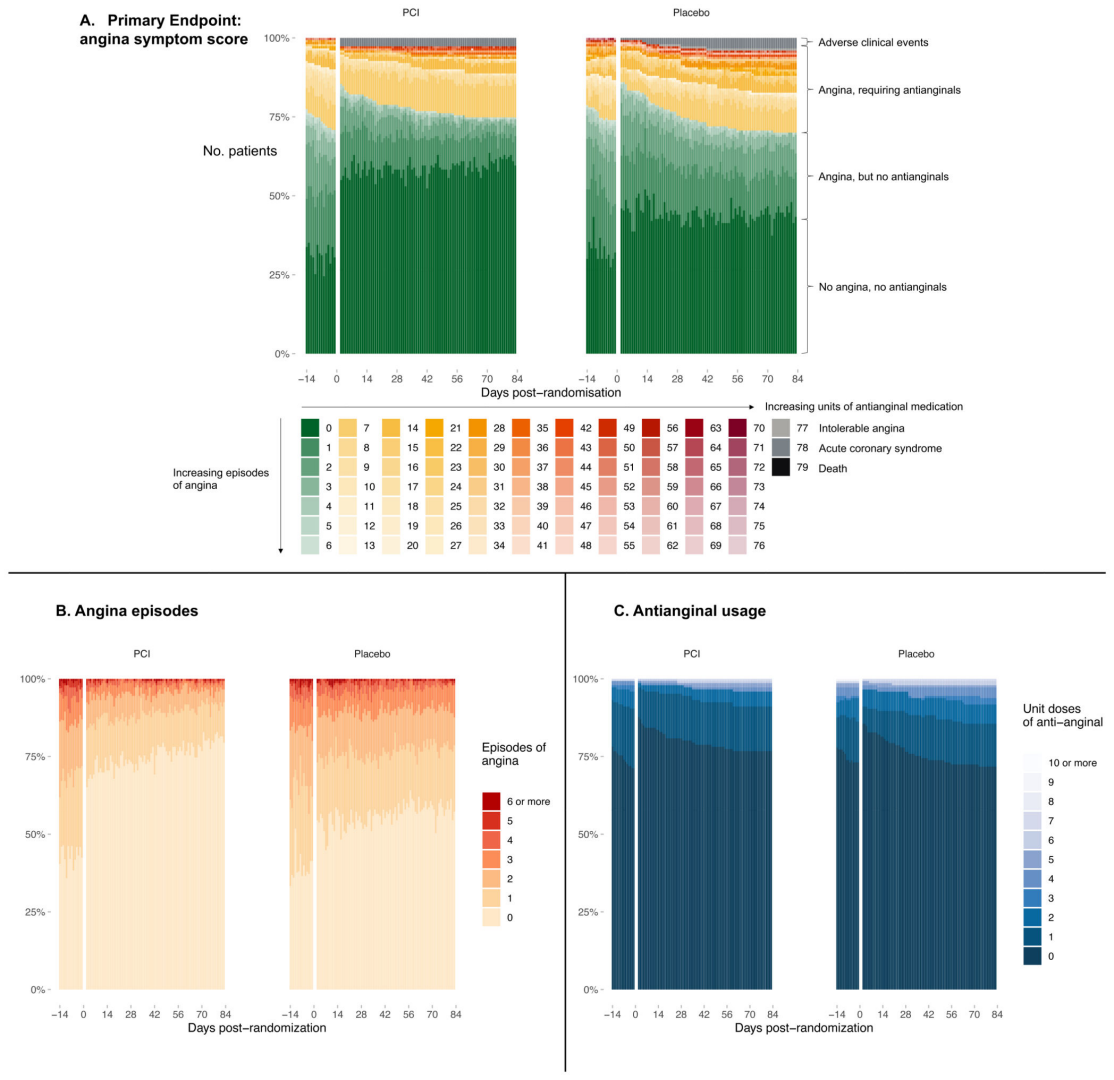
Primary end point of angina symptom score and its constituents. Panel A shows the individual patient data composition of the primary end point (angina symptom score) stratified by treatment allocation. The method for derivation of the score is depicted below and the overall calculated score is shown next to the colored box. Panel B shows individual patient data of daily angina episodes irrespective of the units of antianginal medication prescribed. Panel C shows the number of units of antianginal medications prescribed for each patient on each day of the trial.

Data were available for 99.7% of the 22,823 patient days in the trial. Two participants in the placebo group had missing data. For these participants, the last angina symptom score was carried forward for the primary end point. At 12-week follow-up, the angina symptom score was 2.9 for patients assigned to PCI and 5.6 for patients assigned to placebo (odds ratio, 2.21; 95% confidence interval, 1.41 to 3.47, p<0.001; Table 3, Fig. 1A). Daily angina frequency was 0.3 episodes among patients who received PCI and 0.7 among those who received placebo (odds ratio, 3.44; 95% CI, 2.00 to 5.91; Table 3, Fig. 1B). Daily antianginal medication was 0.2 and 0.3 units among patients who received PCI and placebo, respectively (odds ratio, 1.21; 95% CI, 0.70 to 2.10; Table 3, Fig 1C). The Bayesian longitudinal analysis of the primary end point is provided in Figures S4 through S18 and Table S8. Sensitivity analysis for priors on the treatment effect and anti-anginal medication use are provided in Tables S9 and S10 respectively.

### Secondary end points

**Figure 2:**
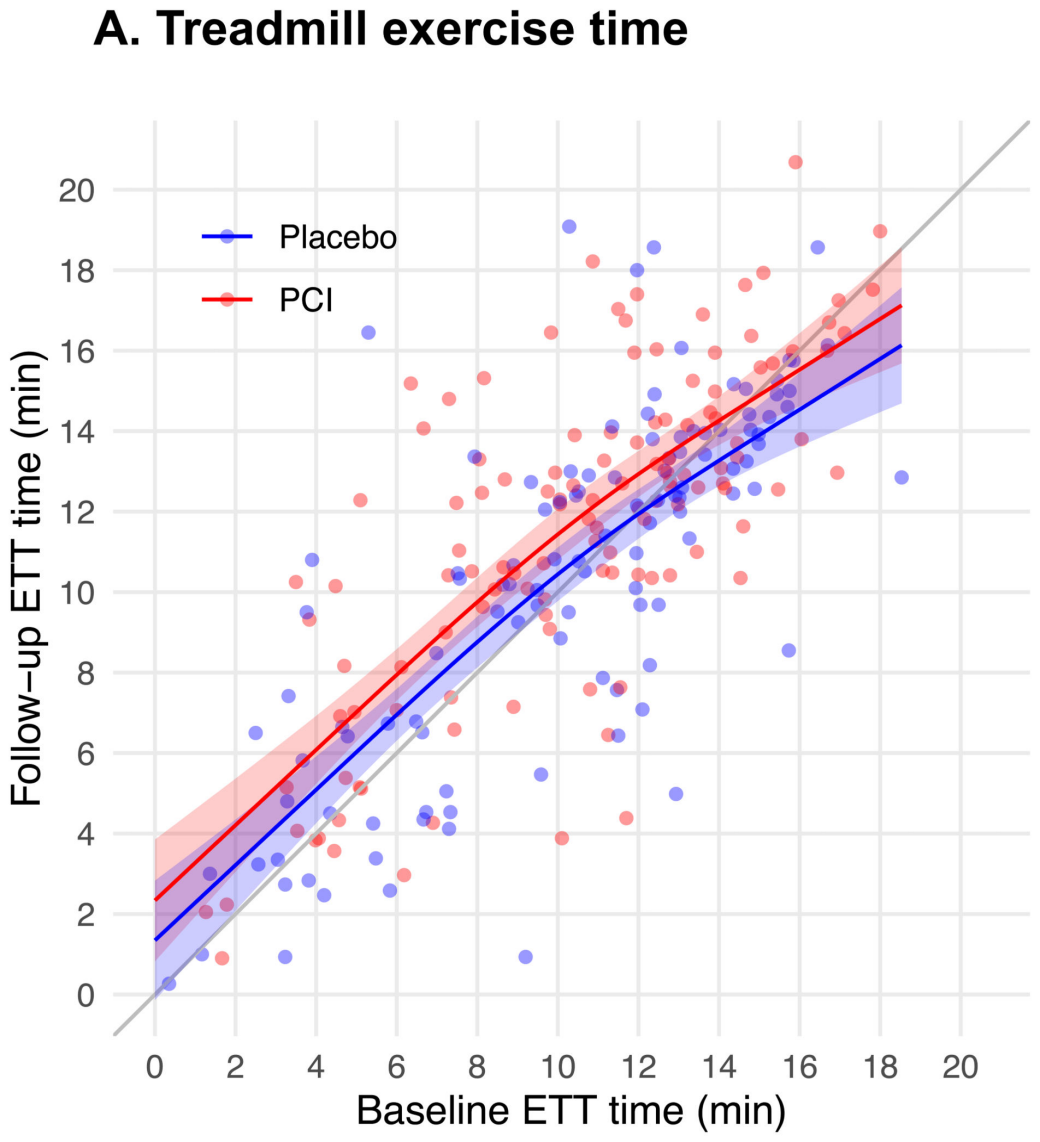
Secondary end points. Panel A shows treadmill exercise at follow-up stratified by baseline performance, with 95% confidence intervals. ETT denotes exercise treadmill time. Panel B shows the distribution of CCS class according to treatment group at pre-randomization and follow-up. CCS denotes Canadian Cardiovascular Society class. The widths of the confidence intervals have not been adjusted for multiplicity. Thus, the confidence intervals should not be used to reject or not reject treatment effects.

The treadmill exercise time (Fig. 2A); physician-assessed CCS class (Fig. 2B); SAQ angina frequency, physical limitation, quality of life and freedom from angina; EQ-5D descriptive system and visual analogue scale, and stress echocardiography score for patients assigned to receive PCI or placebo are shown in Table 3. A sensitivity analysis with multiple imputation for missing data is provided in Table S11. The Bayesian analyses of the secondary end points are provided in Table S8 and Figures S19 through S45.

### Serious Adverse Events

Unblinding for intolerable angina occurred in 0 patients in the PCI group and 1 patient in the placebo group. Acute coronary syndromes occurred in 4 patients in the PCI group and 6 patients in the placebo group. There were no deaths (Table S12). Periprocedural MI (type 4a) occurred in 4 patients in the PCI group and 0 patients in the placebo group. Spontaneous MI (type 1) occurred in 0 patients in the PCI group and 6 patients in the placebo group. In the placebo group, there were two major periprocedural bleeding events and two spontaneous bleeding events on dual antiplatelet therapy. Stroke occurred in two patients in the PCI group and no patients in the placebo group. Pressure wire complications occurred in one patient in the PCI group and two patients in the placebo group. All serious adverse events are reported in Tables S12 and S13. Cases of failure to deliver the randomized therapy are reported in Table S14.

### Blinding Index

The principal assessment of blinding was performed prior to discharge after PCI or placebo. The blinding index for the PCI group was 0.01 (95% CI, -0.05 to 0.06) and the placebo group was -0.09 (95% CI, -0.15 to -0.03) demonstrating effective blinding. The index for blinded staff for patients in the PCI group was 0.01 (95% CI, -0.01 to 0.04) and in the placebo group was 0 (95% CI, -0.03 to 0.03) demonstrating effective blinding. At the end of the 12-week follow-up period, the reassessment values were: 0.19 (95% CI, 0.05 to 0.34) in the PCI group and 0.24 (95% CI, 0.09 to 0.38) in the placebo group. For the corresponding staff groups, it was 0.01 (95% CI, -0.01 to 0.02) and 0 (95% CI, -0.02 to 0.02).

## Discussion

In this placebo-controlled trial in patients with stable angina on little or no antianginal medication, with coronary-artery stenoses causing ischemia, PCI improved the angina symptom score. Reduction in the angina symptom score appeared to result from reduction in daily angina episodes. Assignment to the PCI group was associated with a more than three-fold greater odds of becoming free from angina at the 12-week follow-up. The reduction in angina following PCI was observed immediately and persisted throughout the blinded follow-up period.

The results from ORBITA-2 differed from ORBITA because the studies were designed to answer different questions. In ORBITA, patients adhered to guideline-directed antianginal medications, with PCI as add-on therapy.^21,22^ The small effect size of PCI on exercise time was surprising in the context of larger effects seen in clinical practice and previous clinical trials. One plausible explanation is that the previous experience was unblinded and therefore augmented by the placebo effect. While guidelines recommend escalating antianginal medications for recurrent symptoms, approximately half of patients undergoing PCI are on zero or one antianginal medication.^23^ Achieving the high levels of antianginals applied in ORBITA is challenging.^24^ In fact, patients enrolled in ORBITA-2 had been referred for PCI whilst taking an average of just 1 full dose antianginal medication. Analogous to renal denervation trials,^25,26^ measurement of the efficacy of PCI on angina in a setting that is free from both placebo and the attenuating effect of background antianginal medication, required a trial with the ORBITA-2 study design.

ORBITA-2 introduces a new end point, informed by patient and public engagement and involvement, that is centered on contemporaneous documentation of daily angina on a smartphone application. This has several advantages: high temporal fidelity of data, minimizing recall bias and maximizing data completeness. This tool is being employed in several clinical trials (NCT05459051, NCT04280575, NCT04892537). The ordinal angina symptom score builds on these daily symptom data, incorporating antianginal medication use and relevant clinical events.

The ORBITA trial demonstrated the ethical basis, feasibility and necessity of placebo-controlled trials for studies examining PCI.^8,13,27^ ORBITA-2 builds on this by demonstrating the ethical basis, feasibility, and necessity of testing a coronary interventional procedure without background therapy that may attenuate its effect. Only by not mandating guideline-directed antianginal medication as a precondition for PCI,^28^ could we test its unattenuated efficacy on angina. The two trials together reveal that the recommendation to restrict PCI to patients with inadequate response to antianginal medications may be inadvertently selecting the cohort with the least to gain.

However, despite decades of technical advances in PCI, including the introduction of stents, the effect of PCI on exercise time in the blinded ORBITA-2 trial is still 37 seconds lower than the 96 second effect attributed to balloon angioplasty in the unblinded Angioplasty Compared to Medicine (ACME) trial performed three decades ago.^4^ The effect of PCI as monotherapy was a 59.5 second increment in treadmill exercise time, similar to the 48 to 55 seconds achieved with a full-dose single antianginal medication.^29,30^

With background antianginal medications and PCI, 61% of patients in ORBITA had residual symptoms. In ORBITA-2, with PCI and antianginal medications only if required, 59% still had residual symptoms. Notably, there was no difference in antianginal medication use between the PCI and placebo groups. In both trials, the PCI group had near normalized ischemia detected by stress echocardiography. These trials did not ascertain the cause of the residual symptoms. Perhaps for angina relief, the first therapy administered, either antianginal medication or an antianginal procedure, such as PCI, has the greatest chance of efficacy.

Our study had limitations. The study follow-up period ran for only a 12-week follow-up period. However, the daily data showed that the effect of PCI was immediate and sustained. The study also ceased antianginal medications against guideline recommendations. However, only this design allowed PCI to be tested as antianginal monotherapy. Withdrawal of antianginal medication may have led to unmeasured behavioral changes. The use of nitroglycerin spray was recorded as part of the SAQ but not included in the angina symptom score. While patients with single and multivessel disease were enrolled, 80% had ischemia in a single territory when tested systematically, similar to routine clinical practice.^31^ The smartphone symptom application was only available in English; translation was provided as necessary.

In summary, among patients with stable angina on little or no antianginal medication and objective evidence of ischemia, PCI improved the angina symptom score compared to placebo.

## Supplementary Material

Supplement

## Figures and Tables

**Figure:**
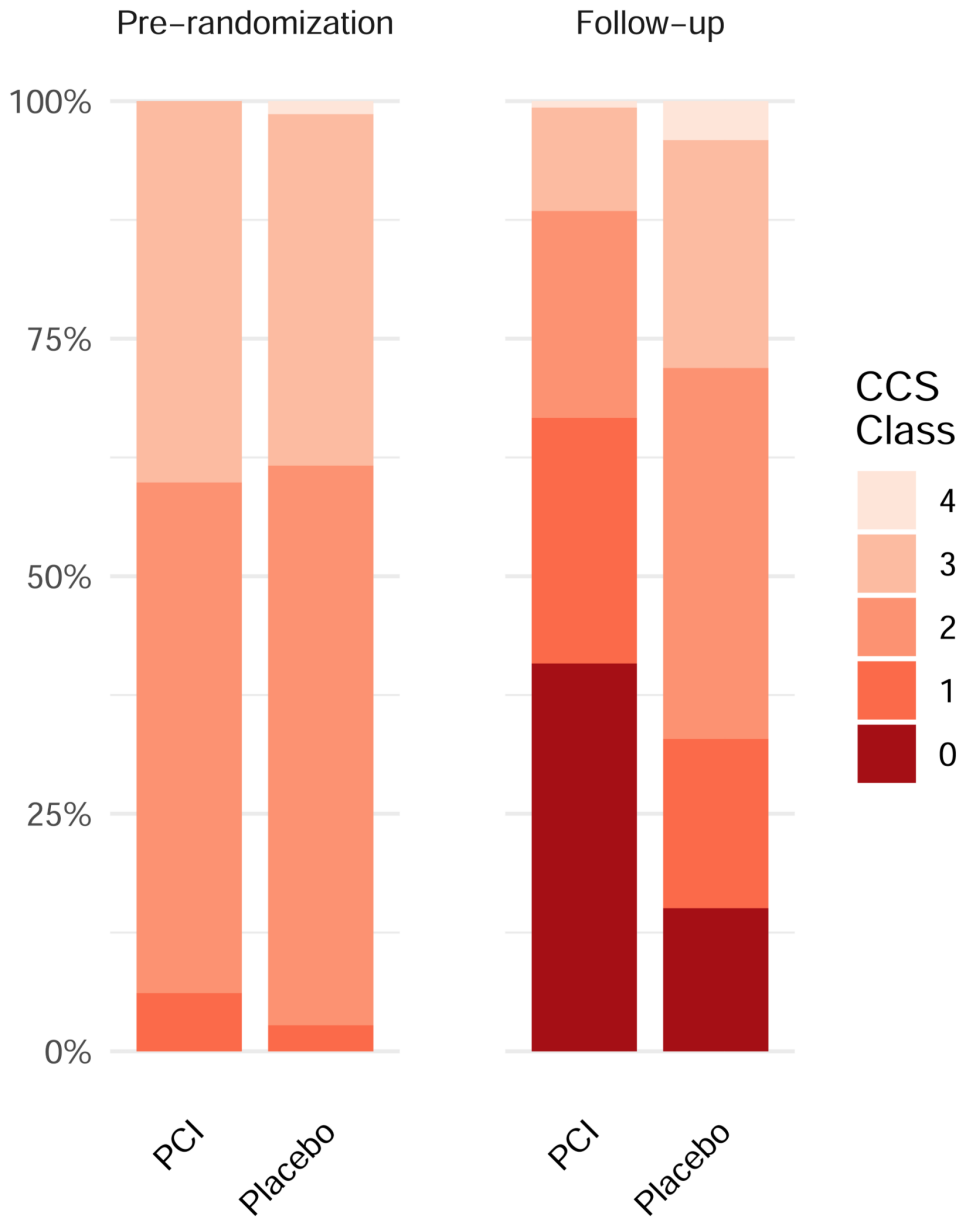


**Table 1:** Baseline Patient Characteristics*

	PCI (N=151)	Placebo (N=150)	Overall (N=301)
Age — yr	65±9	64±9	64±9
Male — no. (%)	120 (79)	118 (79)	238 (79)
Hypertension — no. (%)	97 (64)	92 (61)	189 (63)
Diabetes — no. (%)			
Non-insulin dependent	40 (26)	24 (16)	64 (21)
Insulin-dependent	9 (6)	11 (7)	20 (7)
Hyperlipidemia — no. (%)	113 (75)	104 (69)	217 (72)
Smoking Status — no. (%)			
Never smoked	65 (43)	50 (33)	115 (38)
Ex-smoker	67 (44)	84 (56)	151 (50)
Current smoker	19 (13)	16 (11)	35 (12)
Left ventricular systolic function — no. (%)			
Normal	144 (95)	146 (97)	290 (96)
Mild impairment	6 (4)	3 (2)	9 (3)
Moderate impairment	1 (1)	1 (1)	2 (1)
Canadian Cardiovascular Society Class — no. (%)			
I	10 (7)	1 (1)	11 (4)
II	87 (58)	87 (58)	174 (58)
III	54 (36)	62 (41)	116 (39)
Angina duration (IQR) — months	8 (4-14)	8 (5-14)	8 (5-14)

* Plus–minus values are means ±SD. Results may not total to 100% due to rounding. PCI denotes percutaneous coronary intervention. Left ventricular systolic function was defined as normal (≥55%), mildly impaired (45-54%) or moderately impaired (35-44%).

**Table 2:** Procedural Characteristics*

	PCI (N=151)	Placebo (N=150)	Overall (N=301)
No. vessels — no. (%)			
Single vessel disease	122 (81)	120 (80)	242 (80)
Two vessel disease	25 (17)	27 (18)	52 (17)
Three vessel disease	4 (3)	3 (2)	7 (2)
Vessels — no. (%)			
Left anterior descending	108 (56)	103 (54)	211 (55)
Circumflex	16 (8)	17 (9)	33 (9)
Right coronary artery	42 (22)	43 (23)	85 (22)
Branch vessels	27 (14)	27 (13)	54 (14)
Serial Stenoses	29 (19)	20 (14)	49 (16)
Quantitative coronary angiography diameter stenosis			
Percentage	61±18	62±17	61±18
Median (IQR) — %	60 (48-74)	63 (50-74)	61 (49-74)
Quantitative coronary angiography area stenosis			
Percentage	80±15	82±15	81±15
Median (IQR) — %	83 (73-92)	85 (75-93)	84 (74-92)
FFR			
Mean	0.60±0.16	0.62±0.16	0.61±0.16
Median — IQR	0.61 (0.47-0.74)	0.65 (0.51-0.75)	0.63 (0.49-0.75)
No. vessels assessed — no./total no.	178/193	171/190	349/383
iFR*			
Mean	0.68±0.22	0.71±0.23	0.70±0.22
Median — IQR	0.76 (0.50-0.86)	0.81 (0.58-0.89)	0.78 (0.55-0.87)
No. vessels assessed — no./total no.	178/193	174/190	352/383
Interventions			
No. stents implanted — IQR	2 (1-2)	-	-
Total length of stent implanted (IQR) — mm	42 (23-64)	-	-
Stent diameter (IQR) — mm Median (IQR)	3.0 (2.5-3.5)	-	-
Post-dilation — no./total no. (%)	242/284 (85)	-	-
Intravascular imaging — no./total no. (%)	104/151 (69)		
Drug eluting stent type — no. (%)			
Everolimus-eluting	171 (60)	-	-
Zotarolimus-eluting	83 (29)	-	-
Others	29 (10)		

* Plus–minus values are means ±SD. PCI denotes percutaneous coronary intervention, FFR fractional flow reserve, iFR instantaneous wave-free ratio.

* Where iFR was not available, an alternative non-hyperemic pressure ratio was utilized.

**Table 3:** Primary and Secondary End Points

	PCI	Placebo	Odds ratio or difference (95% CI)	P Value
Angina symptom score
— score/total no. patients	2.9/151	5.6/150	2.21 (1.41-3.47)	<0.001
Daily angina episodes — no./total no. patients	0.3/151	0.7/150	3.44 (2.00-5.91)	-
Daily antianginal medication — units/total no. patients	0.2/151	0.3/150	1.21 (0.70-2.10)	-
**Secondary End Points**				
Treadmill exercise time — seconds/total no. patients	700.9/123	641.4/112	59.5 (16.0-103.0)	-
Canadian Cardiovascular Society class — class/total no. patients	0.9/147	1.7/146	3.76 (2.43-5.82)	-
SAQ angina frequency — score/total no. patients	80.6/146	66.2/145	14.4 (9.5-19.4)	-
SAQ physical limitation — score/total no. patients	82.7/139	73.9/144	8.8 (4.7-12.9)	-
SAQ angina stability— score/total no. patients	61.8/145	55.3/145	6.5 (0.5-12.5)	-
SAQ quality of life— score/total no. patients	62.8/145	51.6/145	11.2 (6.2-16.1)	-
SAQ freedom from angina* — %/total no. patients	39.8/146	15.2/145	3.69 (2.10-6.46)	-
EQ-5D descriptive system— score/total no. patients	0.82/145	0.73/144	0.09 (0.05-0.13)	-
EQ-VAS— score/total no. patients	73.1/146	66.9/143	6.2 (2.4-10.0)	-
Stress echocardiography score— score/total no. patients	0.79/119	1.95/111	-1.17 (-1.56--0.78)	-

PCI denotes percutaneous coronary intervention, SAQ denotes Seattle Angina Questionnaire, EQ-5D denotes EuroQOL 5 dimensions, and EQ-VAS denotes EuroQOL visual analogue scale.

Treadmill exercise time and stress echocardiography score are presented for the patients who had both pre-randomization and follow-up scores.

The Canadian Cardiovascular Society class ranges from 0 to IV where class 0 denotes no angina and class IV denotes angina at rest. SAQ scores range from 0 to 100, with higher scores indicating better health status. European Quality of Life–5 Dimensions (EQ-5D) descriptive system values range from 0-1, and on the EQ-VAS values range from 0 to 100, with higher scores indicating better health status. The derivation of the stress echocardiography score has been previously published^14^

* Calculated as SAQ angina frequency of 100.

For all end points, the follow up values are derived from their respective models and a typical patient. A typical patient was taken to be the mean patient at baseline for treadmill exercise time and stress echocardiography score, and the median patient for all other end points.

The widths of the confidence intervals have not been adjusted for multiplicity. Thus, the confidence intervals should not be used to reject or not reject treatment effects.
