# Supplementary material for: A Placebo-controlled Trial of Percutaneous Coronary Intervention for Stable Angina
Source: N Engl J Med. Author manuscript; Available in PMC 2023 Dec 21. (PMC7615400; doi:10.1056/NEJMoa2310610)
Supplement: Supplement [file EMS190971-supplement-Supplement.pdf]

Supplementary Appendix

Supplement to: Rajkumar CA, Foley MJ, Ahmed-Jushuf F, et al. A Placebo-Controlled Trial of Percutaneous Coronary Intervention for Stable Angina. N Eng J Med. DOI:

This appendix has been provided by the authors to give readers additional information about the work.

Table of Contents

ORBITA-2 INVESTIGATORS 3
TRIAL STEERING COMMITTEE 5
TRIAL MANAGEMENT GROUP 5
DATA AND SAFETY MONITORING BOARD 6
STATISTICAL WORKING GROUP 6
WRITING COMMITTEE 6
CORE LABORATORIES 7
Stress Echocardiography Core Laboratory 7
Angiography Core Laboratory 7
ACKNOWLEDGEMENTS: 8
INCLUSION AND EXCLUSION CRITERIA 9
DERIVATION OF THE ORDINAL SCALE PRIMARY END POINT 10
SMARTPHONE APPLICATION DESCRIPTION 11
MEDICATION PRESCRIBING STANDARD OPERATING PROCEDURE FOR ORBITA-2. 12
MECHANISMS OF BLINDING 14
Blinding index assessment 14
STATISTICAL METHODS FOR BAYESIAN ANALYSIS 15
Primary endpoint 15
Components of the primary end point 16
Secondary end points 17
Presentation of results from the cumulative probability models in units of the original scale 17
General notes 17

SUPPLEMENTARY FIGURES **19**

Supplementary Figure S1: Screenshots from the ORBITA-2 smartphone application 19
Supplementary Figure S2: Consort diagram 20
CORONARY ANGIOGRAPHY IMAGES 21
Supplementary Figure S3: Coronary angiography from all 301 randomized patients 21
PRIMARY END POINT: ANGINA SYMPTOM SCORE 55
Supplementary Figure S4: Daily transition odds ratios for angina symptom score 55
Supplementary Figure S5: Regression model and coefficients for angina symptom score 56
Supplementary Figure S6: Assessment of model fit by predicted state occupancy for angina symptom score. 56
Supplementary Figure S7: Coefficient density plots: angina symptom score 57
Supplementary Figure S8: Chain plot of Markov chain Monte Carlo draws for angina symptom score 57
COMPONENTS OF PRIMARY END POINT: DAILY ANGINA EPISODES 58
Supplementary Figure S9: Daily transition odds ratios for daily angina episodes 58
Supplementary Figure S10: Regression model and coefficients for daily angina episodes 58
Supplementary Figure S11: Assessment of model fit by predicted state occupancy for daily angina episodes 59
Supplementary Figure S12: Coefficient density plots: daily angina episodes 59
Supplementary Figure S13: Chain plot of Markov chain Monte Carlo draws for daily angina episodes 60
COMPONENTS OF PRIMARY END POINT: DAILY ANTIANGINAL MEDICATION UNITS 61
Supplementary Figure S14: Daily transition odds ratios for daily antianginal medication units 61
Supplementary Figure S15: Regression model and coefficients for daily antianginal medication units 61
Supplementary Figure S16: Assessment of model fit by predicted state occupancy for daily antianginal medication units 62
Supplementary Figure S17: Coefficient density plots: daily antianginal medication units 62
Supplementary Figure S18: Chain plot of Markov chain Monte Carlo draws for daily antianginal medication units 63
SECONDARY END POINTS - TREADMILL EXERCISE TIME 64
Supplementary Figure S19: Regression model and coefficients for treadmill exercise time 64
Supplementary Figure S20: Coefficient density plots: treadmill exercise time 64

Supplementary Figure S21: Chain plot of Markov chain Monte Carlo draws for treadmill exercise time 65
Secondary End Points - Canadian Cardiovascular Society (CCS) Class 66
Supplementary Figure S22: Regression model and coefficients for CCS Class 66
Supplementary Figure S23: Coefficient density plots: CCS Class 66
Supplementary Figure S24: Chain plot of Markov chain Monte Carlo draws for CCS Class 67
Secondary End Points - Seattle Angina Questionnaire (SAQ) angina frequency 68
Supplementary Figure S25: Regression model and coefficients for SAQ angina frequency 68
Supplementary Figure S26: Coefficient density plots: SAQ angina frequency 68
Supplementary Figure S27: Chain plot of Markov chain Monte Carlo draws for SAQ angina frequency 69
Secondary End Points - Seattle Angina Questionnaire (SAQ) physical limitation 70
Supplementary Figure S28: Regression model and coefficients for SAQ physical limitation 70
Supplementary Figure S29: Coefficient density plots: SAQ physical limitation 70
Supplementary Figure S30: Chain plot of Markov chain Monte Carlo draws for SAQ physical limitation 71
Secondary End Points - Seattle Angina Questionnaire (SAQ) angina stability 72
Supplementary Figure S31: Regression model and coefficients for SAQ angina stability 72
Supplementary Figure S32: Coefficient density plots: SAQ angina stability 72
Supplementary Figure S33: Chain plot of Markov chain Monte Carlo draws for SAQ angina stability 73
Secondary End Points - Seattle Angina Questionnaire (SAQ) quality of life 74
Supplementary Figure S34: Regression model and coefficients for SAQ quality of life 74
Supplementary Figure S35: Coefficient density plots: SAQ quality of life 74
Supplementary Figure S36: Chain plot of Markov chain Monte Carlo draws for SAQ quality of life 75
Secondary End Points - EQ-5D descriptive system 76
Supplementary Figure S37: Regression model and coefficients for EQ-5D descriptive system 76
Supplementary Figure S38: Coefficient density plots: EQ-5D descriptive system. 76
Supplementary Figure S39: Chain plot of Markov chain Monte Carlo draws for EQ-5D descriptive system 77
Secondary Endpoints - EQ-VAS 78
Supplementary Figure S40: Regression model and coefficients for EQ-VAS 78
Supplementary Figure S41: Coefficient density plots: EQ-5D descriptive system 78
Supplementary Figure S42: Chain plot of Markov chain Monte Carlo draws for EQ-5D descriptive system 79
Secondary End Points - Stress echocardiography score 80
Supplementary Figure S43: Regression model and coefficients for stress echocardiography score 80
Supplementary Figure S44: Coefficient density plots: stress echocardiography score 80
Supplementary Figure S45: Chain plot of Markov chain Monte Carlo draws for stress echocardiography score 81

SUPPLEMENTARY TABLES **83**

Supplementary Table S1: Trial sites 83
Supplementary Table S2: DITTO blinding framework for the catheterization laboratory 85
Supplementary Table S3: Antianginal medication quantification 87
Supplementary Table S4: Derivation of the ordinal scale primary endpoint 88
Supplementary Table S5: Representativeness of Study Participants 91
Supplementary Table S6: Blood results for randomized patients 92
Supplementary Table S7: Post-PCI coronary physiology 93
Supplementary Table S8: Bayesian analysis of primary and secondary end points. 94
Supplementary Table S9: Sensitivity analysis for priors on treatment effect. 98
Supplementary Table S10: Antianginal medication use. 99
Supplementary Table S11: Sensitivity analysis with multiple imputation for missing data. 100
Supplementary Table S12: Serious adverse events contributing to primary end point. 101
Supplementary Table S13: Serious adverse events not contributing primary end point. 102
Supplementary Table S14: Failure to deliver randomized therapy. 104

REFERENCES **106**

ORBITA-2 Investigators

Imperial College London
Christopher Rajkumar, Michael Foley, Fiyyaz Ahmed-Jushuf, Florentina Simader, Sashiananthan Ganesananthan, Danqi Wang, Muhammad Mohsin, Rachel Pathimagaraj, Brian Wang, Krzysztof Macierzanka, Ricardo Petraco, Ramzi Khamis, Graham Cole, James Howard, Jamil Mayet, Darrel Francis, Rasha Al-Lamee

Imperial College London Healthcare NHS Trust
Arif Kokhar, Aisha Gohar, Ioannis Lampadakis, Henry Seligman, Amit Kaura, Sukhjinder Njjer, Sayan Sen, Punit Ramrakha, Raffi Kaprielian, Iqbal Malik, Masood Khan, Amarjit Sethi, Rodney Foale

Mid and South Essex NHS Foundation Trust
Thomas Keeble, Kare Tang, John Davies, Reto Gamma, Gerald Clesham, Jason Dungu, Alamgir Kabir, Shah Mohd Nazri

University Hospitals of Dorset NHS Foundation Trust
Peter O'Kane, Jonathan Hinton, Jehangir Din

Barking Havering and Redbridge University Hospitals NHS Trust
Alexandra Nowbar

Royal Free London NHS Foundation Trust
Tushar Kotecha

Portsmouth Hospitals University NHS Trust
Peter Haworth

St George's University Hospitals NHS Foundation Trust
James Spratt, Rupert Williams, Claudia Cosgrove, Pitt Lim

Worcestershire Acute Hospitals NHS Trust
Helen Routledge, Lal Mughal, Jasper Trevelyan

Salisbury NHS Foundation Trust
Manas Sinha

University Hospital Southampton NHS Foundation Trust
Nick Curzen, James Wilkinson, Rohit Sirohi, Alison Calver, John Rawlins, Richard Jabbour

Royal Berkshire NHS Foundation Trust
Neil Ruparelia

West Hertfordshire Hospitals NHS Trust
Joban Sehmi

Cardiff and Vale University Health Board
Tim Kinnaird

Sandwell and West Birmingham Hospitals NHS Trust
Fairoz Abdul

Royal Brompton and Harefield NHS Foundation Trust
Vasileios Panoulas

Queen Mary University of London
David Collier

Barts Health NHS Trust
Afzal Sohaib

Trial Steering Committee

Dr David Collier (Chair)
Clinical Director; The William Harvey Institute, Queen Mary University of London

Dr Rasha Al-Lamee
Reader and BHF Intermediate Research Fellow; National Heart and Lung Institute, Imperial College London

Dr Afzal Sohaib
Consultant Cardiologist; Barts Health NHS Trust

Mr Patrick McVeigh
Patient Representative

Dr Christopher Rajkumar
Clinical Research Fellow; Imperial College London

Dr Alexandra Nowbar
Cardiology Fellow; Barking Havering and Redbridge University Hospitals NHS Trust

Dr Thomas Keeble
Consultant Cardiologist; Essex Cardiothoracic Centre, Mid and South Essex NHS Foundation Trust

Professor Darrel Francis
Professor of Cardiology; National Heart and Lung Institute, Imperial College London

Trial Management Group

Dr Christopher Rajkumar
Clinical Research Fellow; Imperial College London

Dr Michael Foley
Clinical Research Fellow; Imperial College London

Dr Fiyyaz Ahmed-Jushuf
Clinical Research Fellow; Imperial College London

Dr Florentina Simader
Clinical Research Fellow; Imperial College London

Dr Rasha Al-Lamee
Reader and BHF Intermediate Research Fellow; National Heart and Lung Institute, Imperial College London

Data and Safety Monitoring Board

The Data and Safety Monitoring Board (DSMB) reviewed all serious adverse events and all changes to antianginal medication.

Dr Neil Chapman (Chair)
Honorary Senior Lecturer, Clinical Pharmacology Consultant; Imperial College London

Dr Vikas Kapil
Clinical Senior Lecturer, Clinical Pharmacology Consultant; Queen Mary University of London

Dr Jaymin Shah
Consultant Cardiologist; London North West University Healthcare NHS Trust

Statistical Working Group

Professor Frank E Harrell, Jr.
Professor of Biostatistics; Vanderbilt University School of Medicine

Dr Matthew Shun-Shin
Clinical Senior Lecturer and Consultant Cardiologist; Imperial College London

Dr Rasha Al-Lamee
Reader and BHF Intermediate Research Fellow; National Heart and Lung Institute, Imperial College London

Writing Committee

Dr Christopher Rajkumar
Clinical Research Fellow; Imperial College London

Dr Matthew Shun-Shin
Clinical Senior Lecturer and Consultant Cardiologist; Imperial College London

Professor Darrel Francis
Professor of Cardiology; Imperial College London

Dr Rasha Al-Lamee
Reader and BHF Intermediate Research Fellow; National Heart and Lung Institute, Imperial College London

Core Laboratories

Stress Echocardiography Core Laboratory

Dr Graham Cole
Honorary Senior Lecturer and Consultant Cardiologist; Imperial College London

Dr Joban Sehmi
Consultant Cardiologist; West Hertfordshire Hospitals NHS Trust

Professor Darrel Francis
Professor of Cardiology; Imperial College London

Angiography Core Laboratory

Dr Arif Kokhar
Imperial College Healthcare NHS Trust

Dr Aisha Gohar
Imperial College Healthcare NHS Trust

Dr Ioannis Lampadakis
Imperial College Healthcare NHS Trust

Acknowledgements

ORBITA-2 was an investigator-initiated trial sponsored by Imperial College London. We thank all patients and their families for their dedication and altruism, without which this research would not be possible. The trial was funded by grants from NIHR Imperial Biomedical Research Centre, Medical Research Council, British Heart Foundation, NIHR and the Imperial Coronary Flow Trust. Philips Volcano supplied the coronary pressure wires. We acknowledge the support of the NIHR Clinical Research Network. We thank NAMSA for independent trial monitoring. We are grateful to Nina Bual for her expertise in acquisition of the DSE data. We thank Drs Neil Chapman, Vikas Kapil and Jaymin Shah, comprising the independent data safety monitoring board committee. We thank Professor Frank E. Harrell Jr. for his assistance with the Bayesian analysis. We are grateful to Juliet Holmes for her dedication and support for the trial. We also thank Patrick McVeigh for his work as patient representative for the trial steering committee. We thank the research and administrative teams at each of our 14 trial centers for their patience, dedication, and support. Finally, we thank the National Health Service of the United Kingdom for encouraging and facilitating novel clinical research.

Disclosure forms provided by the authors are available with the full text of this article at NEJM.org.

Inclusion and Exclusion Criteria

Inclusion

ORBITA-2 enrolled participants who were deemed eligible for PCI by their clinical teams and met all 3 of the following criteria:

1. Angina or angina-equivalent symptoms

2. Anatomical evidence of a severe coronary stenosis in at least 1 vessel, either:
   – Invasive diagnostic coronary angiography indicating ≥70% stenosis
   – Computerised tomography coronary angiography (CTCA) indicating severe stenosis

3. Evidence of ischaemia, on any of the following tests:
   – Positive dobutamine stress echocardiography
   – Positive stress perfusion cardiac magnetic resonance imaging (MRI)
   – Positive nuclear medicine myocardial perfusion scan
   – Invasive pressure wire assessment suggestive of ischaemia, as judged by the interventional cardiologist, at the time of clinical or research coronary angiography

Exclusion

1. Age younger than 18
2. Recent acute coronary event (within last 6 months)
3. Previous coronary artery bypass graft surgery
4. Significant left main stem coronary disease
5. Chronic total occlusion in the target vessel
6. Contraindication to percutaneous coronary intervention or drug-eluting stent implantation
7. Contraindication to antiplatelet therapy
8. Severe valvular disease
9. Severe left ventricular systolic impairment (ejection fraction ≤35%)
10. Severe respiratory disease (requiring long term oxygen or symptoms deemed by investigator to be more likely attributable to respiratory disease)
11. Life expectancy less than 2 years, pregnancy, inability to consent

Derivation of the ordinal scale primary end point

The primary end point is the angina symptom score measured daily. This is an ordinal clinical outcome scale of angina health status, ranging from 0 to 79. The daily score is derived from the number of episodes of angina reported by a patient on a given day via the smartphone application, the units of antianginal medication prescribed on that day, and high-level category overrides for unblinding due to intolerable angina, acute coronary syndrome, and death.

The total daily dosage of commonly prescribed antianginal medications considered to be 1 unit is reported in Supplementary Table 3. Full details of the primary end point have been published previously[1]. Supplementary Table 4 reports the composition of each level of the primary end point.

Smartphone application description

The ORBITA-2 symptom smartphone application requires the participant to define their angina in their own words and then report the number of episodes of this symptom for each day of the trial. It also requires the participant to report for each week if they experienced angina with 2 activities that were set by the participant at enrollment as triggering their symptoms.

The symptom application approach permits not only a quantitative assessment of the time-course of angina evolution during the blinded period, but also a time-to-event analysis of occurrence of first angina episode.

Full details regarding development and use of the application have previously published.[2]

For the ORBITA-2 trial, the smartphone application was only available in the English language The smartphone application was intentionally designed to be very simple. An ethically and gender diverse patient focus group with lived experience of coronary artery disease assisted in the design of the smartphone application. Predominantly, participants were able to read, write and speak English. However, there was a minority of participants who could not read, write, or speak English. Most of these patients were assisted by a contact who was capable of translating. If participants did not have a contact who could offer this help, a blinded member of the research team arranged daily data entry using a translator service.

At enrollment, participants completed a training module, with a test component, which demonstrated and documented their ability to understand the app and input data.

Participants had 24/7 access to a blinded member of the trial team for any queries about the smartphone application.

Supplementary figure S1 contains screenshots from the ORBITA-2 symptom application.

Medication prescribing standard operating procedure for ORBITA-2.

This medication management SOP was developed in conjunction with the DSMB and has been previously published.[1]

All medication changes will be made by the research team with informed consent from the participant. Decisions will be discussed with primary care practitioners as necessary.

1. Participants not already taking the following medications will be started on:

Dual antiplatelet therapy:
Standard loading doses will be used. Thereafter, aspirin 75 mg once daily with either clopidogrel 75 mg once daily or ticagrelor 90 mg twice daily or prasugrel 5-10 mg once daily, dose adjusted for age and weight, will be administered.

Gastrointestinal (GI) protection:
If at high risk of adverse GI effects (based on previous GI ulceration, age or concomitant medications that increase risk), participants will be started on a proton pump inhibitor, lansoprazole 30mg once daily, in accordance with NICE guidance on gastro-oesophageal reflux disease and dyspepsia in adults (CG184).

Lipid-lowering medication:
Atorvastatin 80 mg once daily will be preferred. If participants are already taking lower dose atorvastatin, simvastatin or pravastatin, this will be changed to atorvastatin 80 mg once daily. If taking rosuvastatin, this will be continued.

2. Other concomitant risk factor modifying medication

Antihypertensives:
Antihypertensives with antianginal properties will be stopped. Participants will be given a blood pressure monitor and asked to perform home readings. Blood pressure control will be monitored by the research team, and if required, antihypertensives will be added. Agents without antianginal properties will be preferred.

3. Antianginal medication

Regular antianginal medications will be stopped on enrollment. All participants will be given glyceryl trinitrate spray to be used when necessary. The need for starting regular antianginals will be determined by participant preference and patient-reported symptoms.

An individualised protocol for potential introduction of antianginal medications will be prepared for each participant by the research team. This protocol will be based on the participant's medical history, heart rate, blood pressure and any medication intolerance. The preferred sequence will be as follows: Bisoprolol, nifedipine MR, isosorbide mononitrate MR, nicorandil, ranolazine.

Antianginals started prior to randomization will be stopped at randomization and re-introduced according to participant preference and symptoms as described above, by the blinded research team.

Mechanisms of blinding

Placebo optimization strategies are reported in Supplementary Table S2.

Blinding index assessment

Our protocol assessed for accidental leakage of information to staff and to patients. The ward clinical staff were asked to guess the treatment allocation at the time of discharge from the blinded procedure. The blinded research staff were asked to guess the treatment allocation from all information available to them at the follow-up visit prior to speaking to the patient.

Patient blinding was assessed at the time of discharge from the randomised blinded procedure. For completeness the same question was also asked when they attended for follow-up but at that time, they had the benefit of knowing the symptomatic responses and therefore this was no longer strictly a valid measure of blinding.

Patients and staff were asked to guess one of the following: (1) PCI, (2) Placebo, (3) Don't know. Patients and medical staff were asked to state the certainty of their answers grade 1-5 with 5 being most sure.

Statistical analysis of the blinding index was performed using published methods.[3]

Statistical Methods for Bayesian analysis

Primary endpoint

For the primary end point of the angina symptom score we calculate the daily odds ratio of transitioning to a better clinical state.

This was derived by constructing a Bayesian first-order Markov longitudinal ordinal model. This model maximizes power by utilizing daily symptom assessment while accounting for clinical events. The model includes the previous daily score (a first-order Markov model), mean score value during the pre-randomization period, trial day number, and randomization arm.

The Markov model conditions on the "lag 1" (previous day's) response and analyses the transitions as conditionally independent. The dependence is recognised when transition probabilities are converted into state occupancy probabilities. The dependence on the "lag 1" response was modelled using a nearly flat prior (mean of 0, SD of 100).

The trial day number was allowed to interact with the treatment group to allow for differing treatment effect each day. Effects were allowed to be non-linear with restricted cubic splines, and partial proportional odds with constraints with respect to time.

In addition to the daily odds ratio, clinically relevant estimates and contrasts can be drawn from the model. This is illustrated by deriving the number of days in a state (such as no angina, or no antianginal medication use) from the daily transition probabilities.

In addition to the angina symptom score, similar models were constructed for its components: the number of daily episodes of angina (irrespective of antianginal use), and number of standardized doses of antianginal medications. Only raw data are presented for high grade events (unblinding due to intolerable angina, acute coronary syndromes, and deaths).

For the primary end point, evidence of efficacy was expressed with Bayesian posterior probabilities of a beneficial effect of PCI over placebo. This is the probability of an odds ratio > 1 in an ordinal model, which for the primary outcome is identical to the probability of positive increase in days with improved symptoms.

The regression model specifications, output, chain mixing plots, and density plots are included below. The MCMC process used 8 Chains with 4,000 iterations each (with 2,000 burn-in iterations) for the primary endpoint. Goodness of fit was assessed by comparing data simulated from the model with the raw data.

For the angina symptom score model, the covariates were:

- The previous day's value with a restricted cubic spline with 4 knots (at the 5th, 35th, 65th and 95th centiles),
- The day number, which was allowed to interact with…
- Randomization arm,

- The mean angina symptom score value for the patient from the enrolment period with a restricted cubic spline with 4 knots (at the 5th, 35th, 65th and 95th centiles).

The partial proportional odds assumption was relaxed for day and Treatment, with a linear constraint.

The priors were previously specified in the Statistical Analysis Plan:

- *For the intercepts the priors are induced by a Dirichlet distribution on the cell probabilities when all covariates are set to their means. This enforces a strict ordering of the intercepts since they are defined by logits of cell probabilities accumulated over increasing values of the response.*
- *For the treatment effect (log odds ratio (OR)) the prior is normal with mean zero and standard deviation chosen so to that the prior probability that the OR < 0.25 equals the prior probability OR > 4 with both equalling 0.05. Thus, the analysis is skeptical about the treatment effect being large in either direction. Besides being more convincing to a skeptic (should there be evidence for benefit), the skeptical prior "pulls back" the OR more at early data looks to help avoid making a mistake in stopping a treatment arm early.*
- *For covariates a virtually flat prior will be used, I.e., a distribution with mean 0 and standard deviation of 100 on a normalized covariate scale.*

After performing the regression, the number of days in state was calculated by drawing from the model with an exemplar patient undergoing PCI and Placebo with the median baseline covariates (angina symptom score of 1 pre-randomizations). The cell occupancy probabilities were summated for each draw under both conditions and subtracted to derive the difference.

As a sensitivity analysis, to test the impact of the sceptical prior, we also repeated the analysis with a virtually flat prior with mean zero and standard deviation of 100 on a normalized covariate scale.

The first day of analysis was day 2 (with randomization day being day 0). This is because day 0 was the day of the procedure (with the patient in hospital), and some patients remained in hospital on day 1. For day 2, the lagged value was taking from day –1 (i.e., the day before randomization) as the covariates must come from the values available pre-randomization.

Components of the primary end point

In addition to the angina symptom score similar models were constructed for its components: the number of daily episodes of angina (irrespective of antianginal use), and number of standardized doses of antianginal medications.

For the angina episodes model, the covariates were:

- The previous day's value with a restricted cubic spline with 3 knots. As the range of values was limited, the spacing of knots was adjusted to the values 1, 3, and 5.
- The day number, which was allowed to interact with…
- Randomization arm,
- The mean angina episodes during the enrolment period with a restricted cubic spline with 4 knots (at the 5th, 35th, 65th and 95th centiles).

The partial proportional odds assumption was relaxed for day with a linear constraint.

For the number of Standardized antianginal medications model, the covariates were:

- The previous day's value with a restricted cubic spline with 3 knots. As the range of values was limited, the spacing of knots was adjusted to the values 1, 3, and 5.
- The day number, which was allowed to interact with…
- Randomization arm,
- The mean standardized units of antianginal medications during the enrolment period with a restricted cubic spline with 4 knots (at the 5th, 35th, 65th and 95th centiles).

The partial proportional odds assumption was relaxed for day with a linear constraint.

Secondary end points

The secondary end points were measured once at pre-randomization and once at the follow-up visit.

For both continuous and categorical outcome variables, an ordinal (proportional odds) analysis of covariance described was used[4] within a Bayesian framework. This uses a cumulative probability model (also called "cumulative link model") which does not impose distributional assumptions on the outcome.

The follow-up value was conditioned on the pre-randomization value, with non-linearity allowed using a restricted cubic spline with 3 knots (at the 10th, 50th and 90th centile), and randomization arm. We did not allow for interaction.

For the secondary end points 4 chains with 20,000 samples each (with 10,000 burn-in iterations) were used.

Presentation of results from the cumulative probability models in units of the original scale

For clinical interpretation, the contrast between the PCI and placebo groups are presented for a typical patient with either the median or mean baseline value (specified in the table), transformed to the original scale for presentation, rather than the underlying odds ratio. This transformation is performed by using a weighted mean of the possible response levels with weights equal to the cell probabilities estimated from the proportional odds model.

General notes

For all endpoints, evidence for efficacy was quantified with Bayesian posterior probabilities of a beneficial effect of PCI over placebo. This is the probability of an odds ratio > 1 in an ordinal model.

For consistency (as for some scores lower numbers represent a better health state - e.g. angina symptom score, CCS class) contrasts between the randomization arms were constructed so that a higher odds ratio represented an increased probability that the PCI arm would achieve a better health state.

Bayesian results are presented as the posterior mean with the credible interval being constructed from the 95% Highest Posterior Density Interval.
We did not adjust for multiplicity.

The regression model specifications, output, chain mixing plots, and density plots are included below.

Supplementary Figures

Supplementary Figure S1: Screenshots from the ORBITA-2 smartphone application

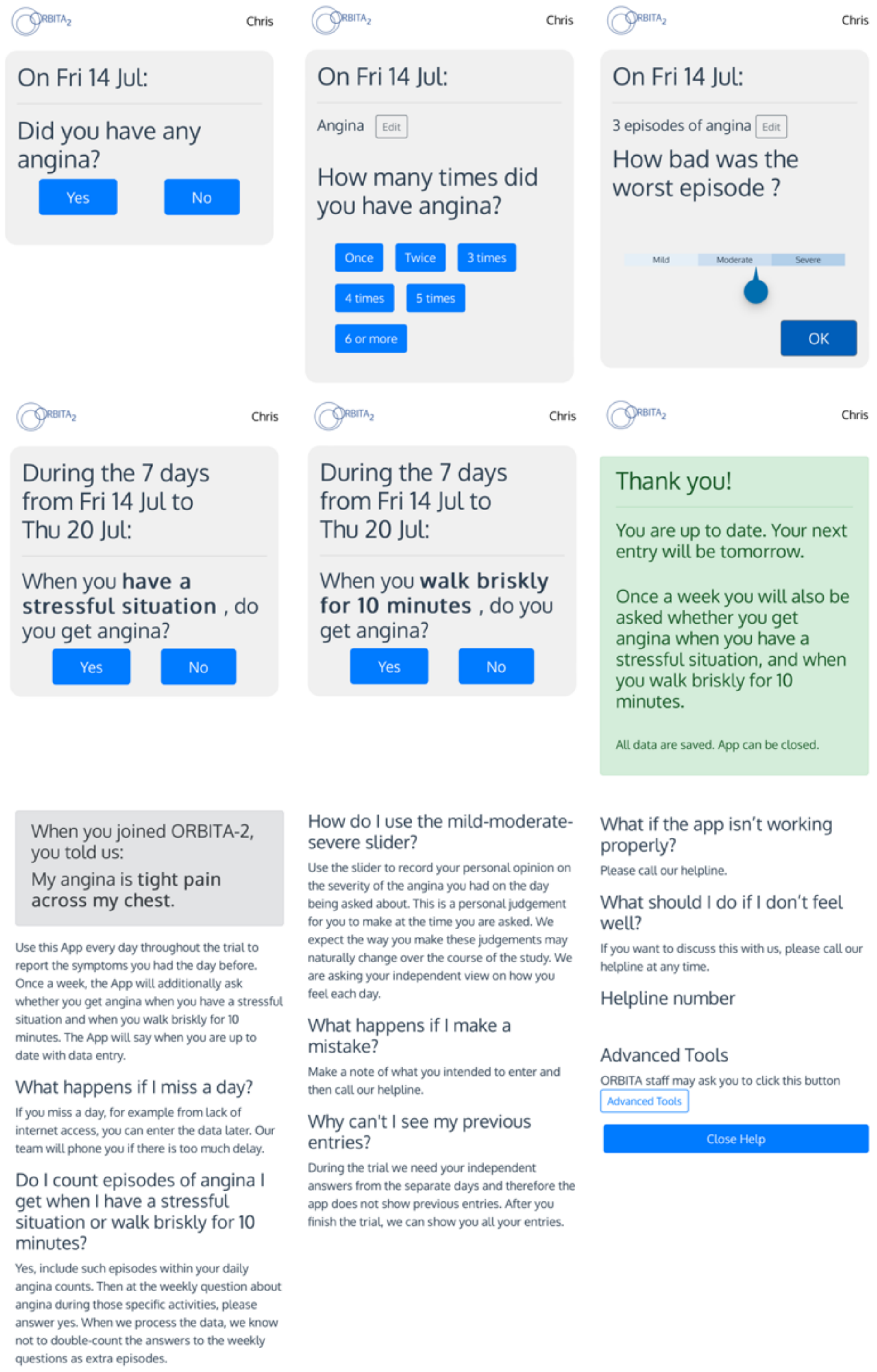

Supplementary Figure S2: Consort diagram

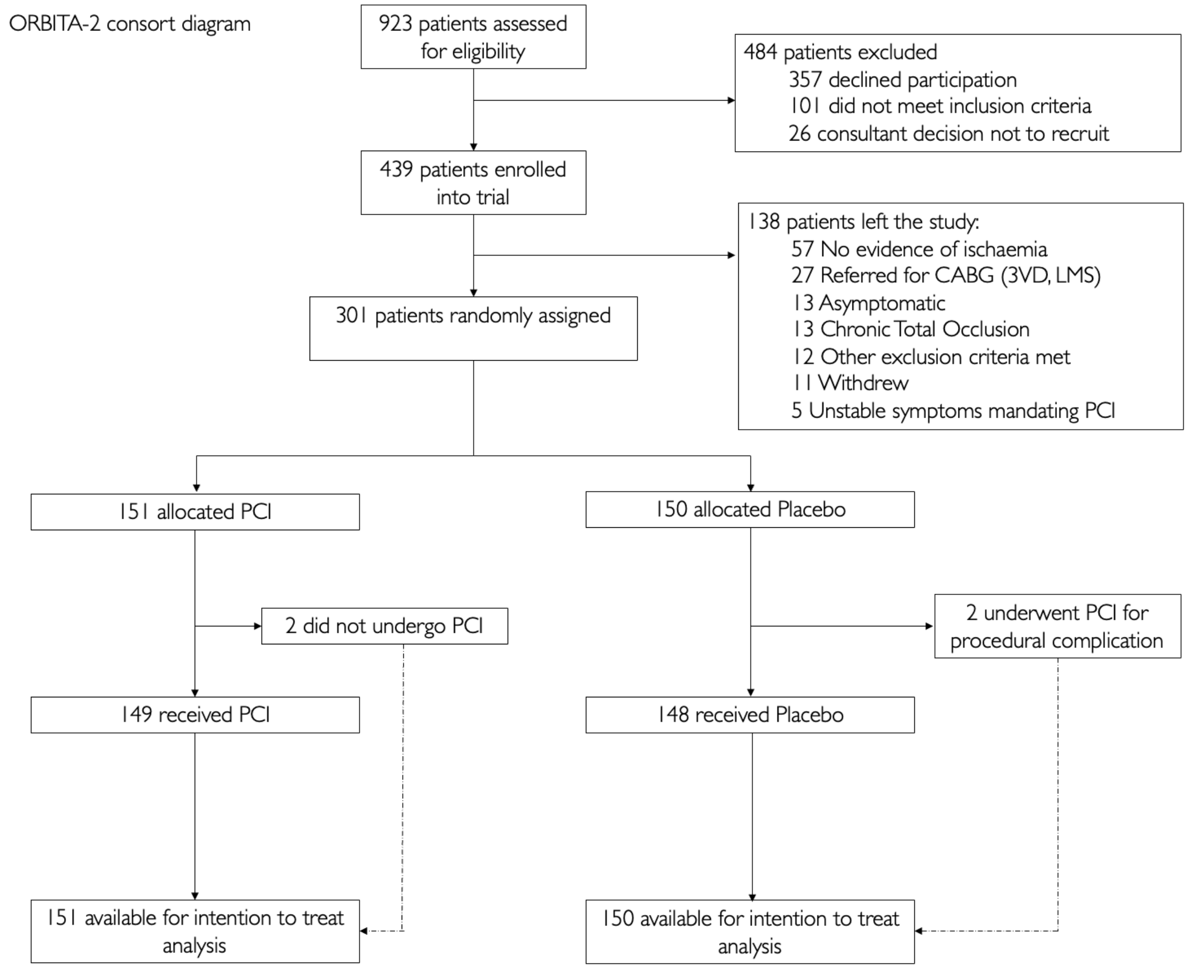

Each excluded participant was allocated a singular reason for exclusion.

Coronary angiography images

Supplementary Figure S3: Coronary angiography from all 301 randomized patients

The angiograms from each of the 301 randomized patients are shown below. Where there was more than one target vessel, multiple projections are shown, labelled sequentially a-c.
* Qualifying coronary-artery lesion.

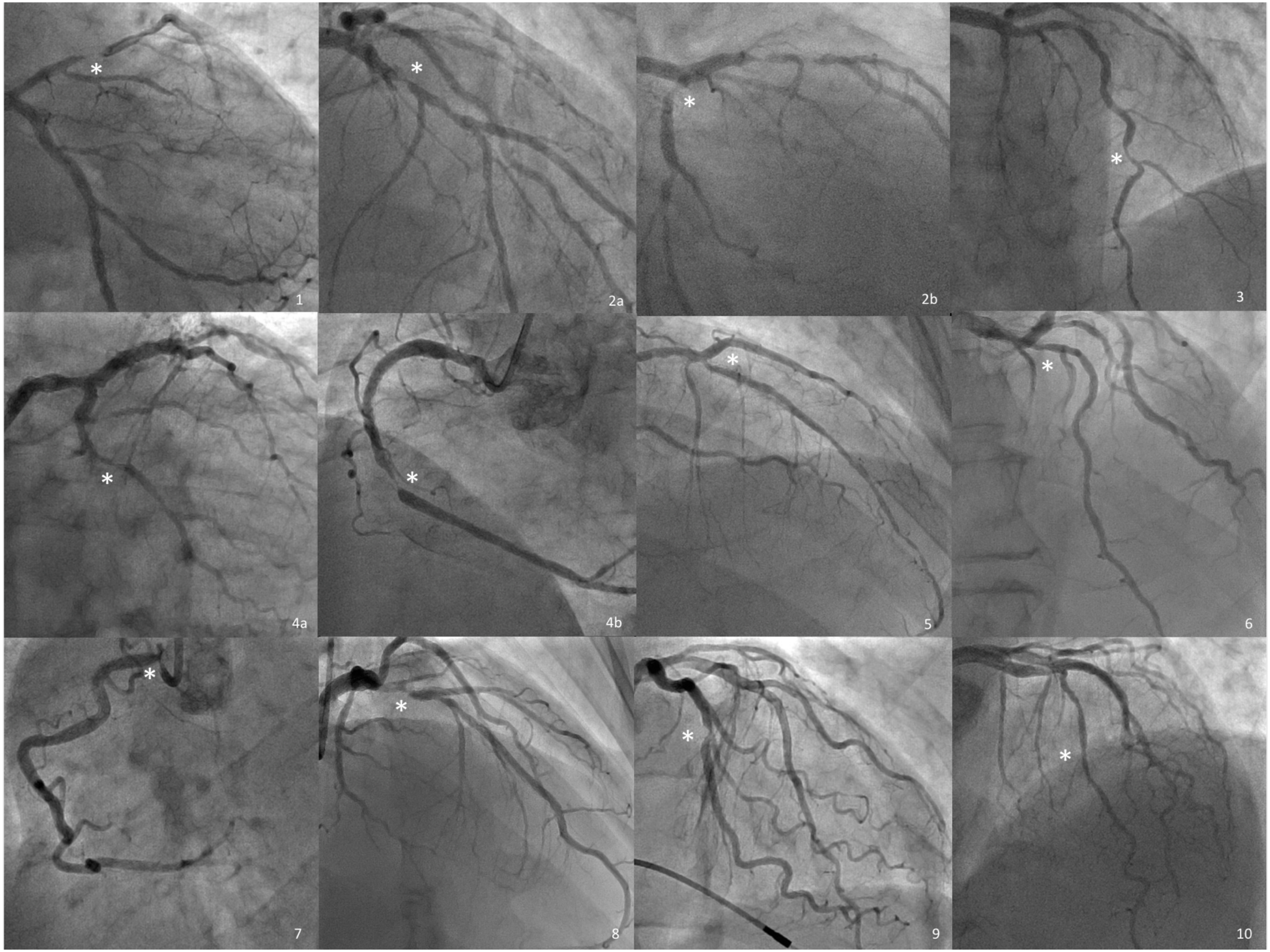
*
1
2a
2b
3
4a
4b
5
6
7
8
9
10

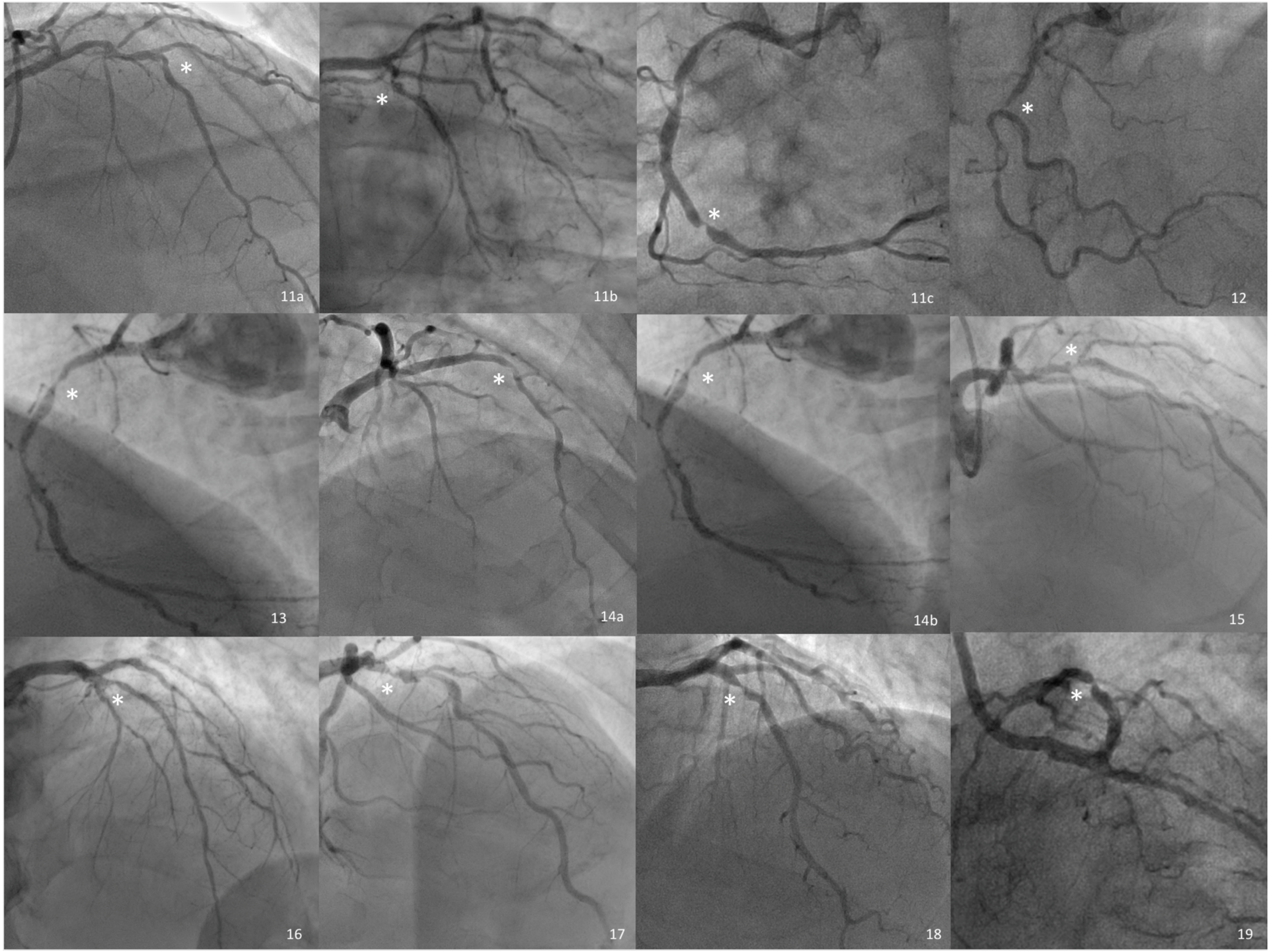
11a
11b
11c
12
13
14a
14b
15
16
17
18
19

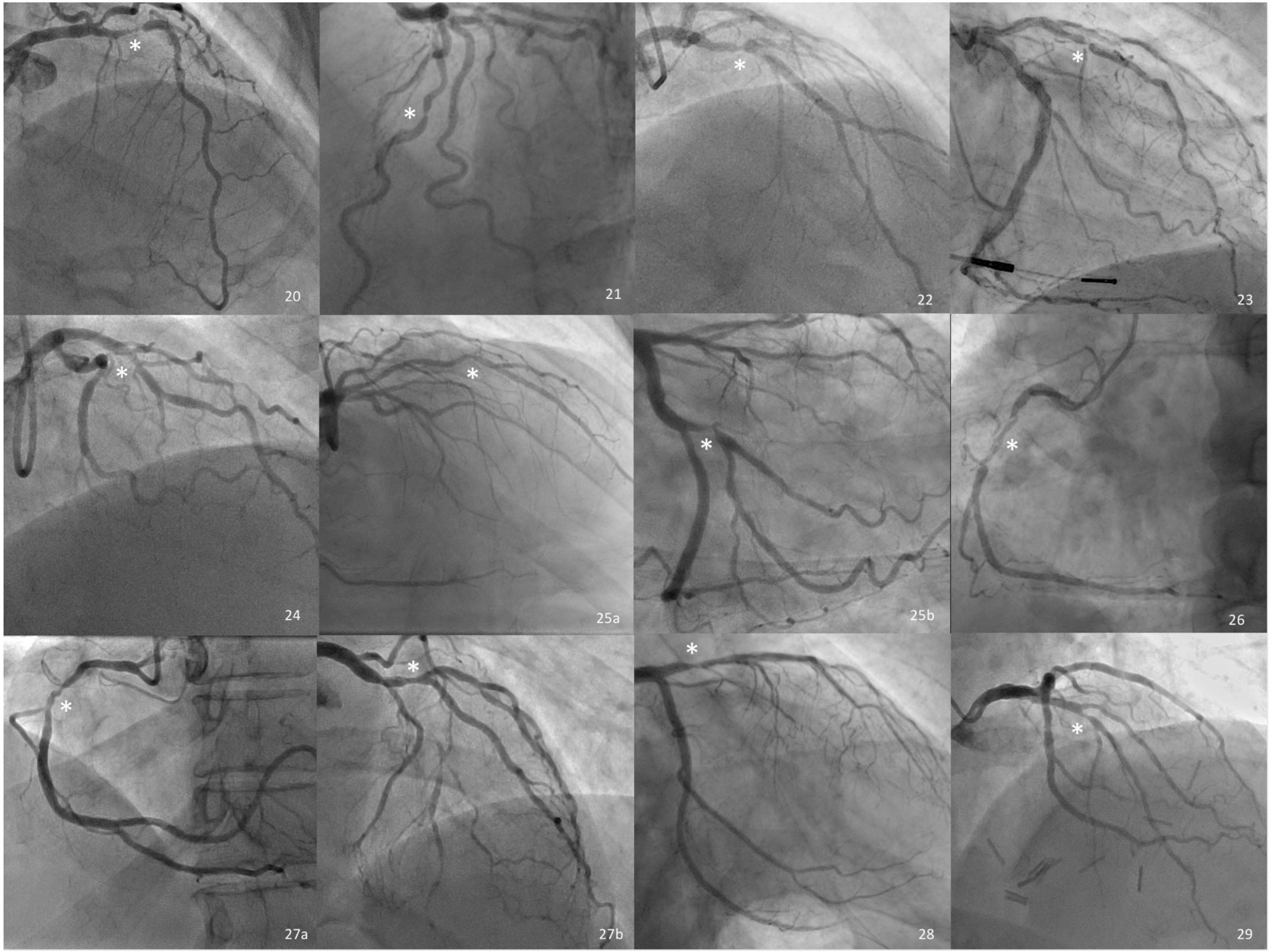
20
21
22
23
24
25a
25b
26
27a
27b
28
29

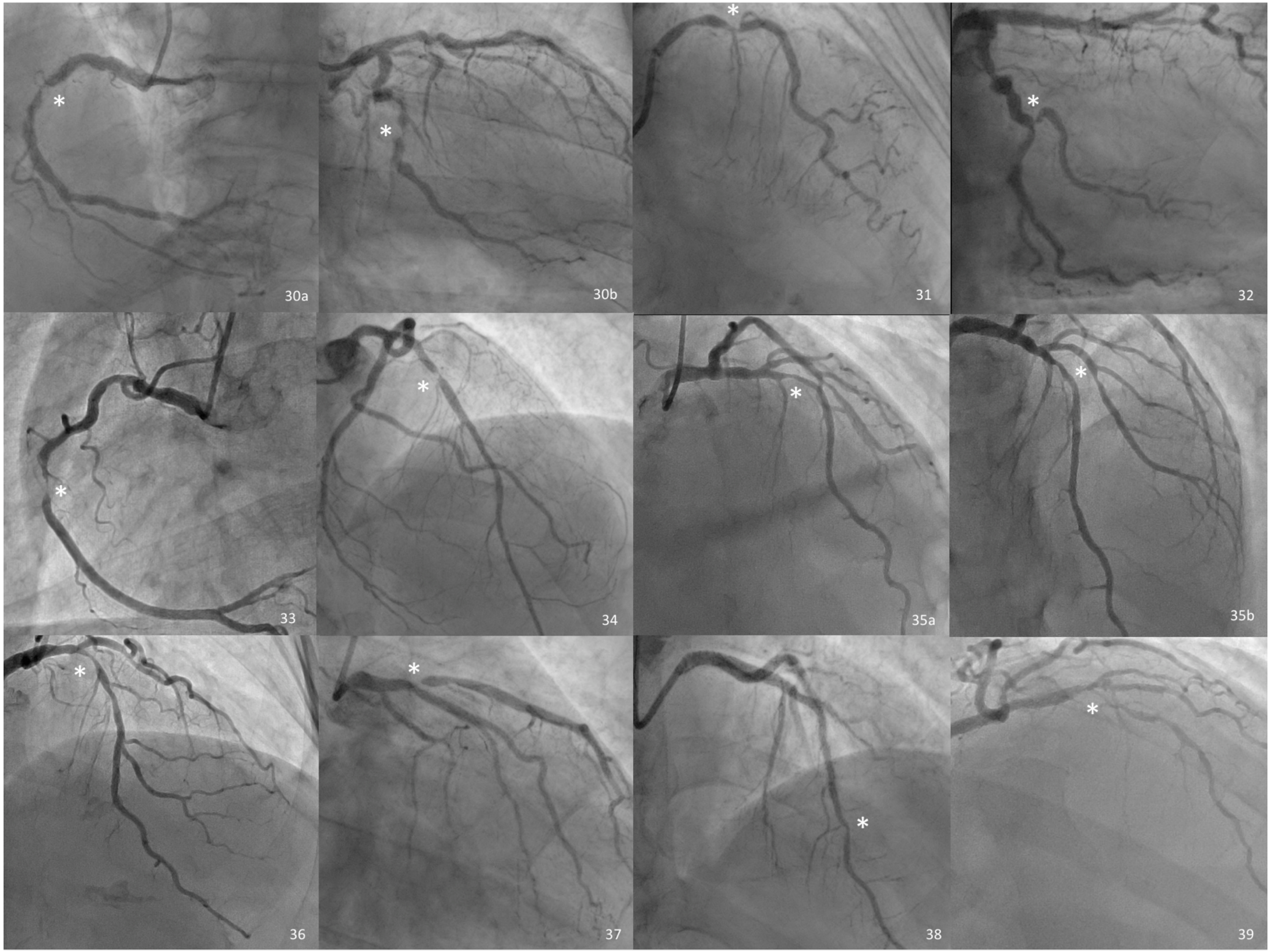
30a
30b
31
32
33
34
35a
35b
36
37
38
39

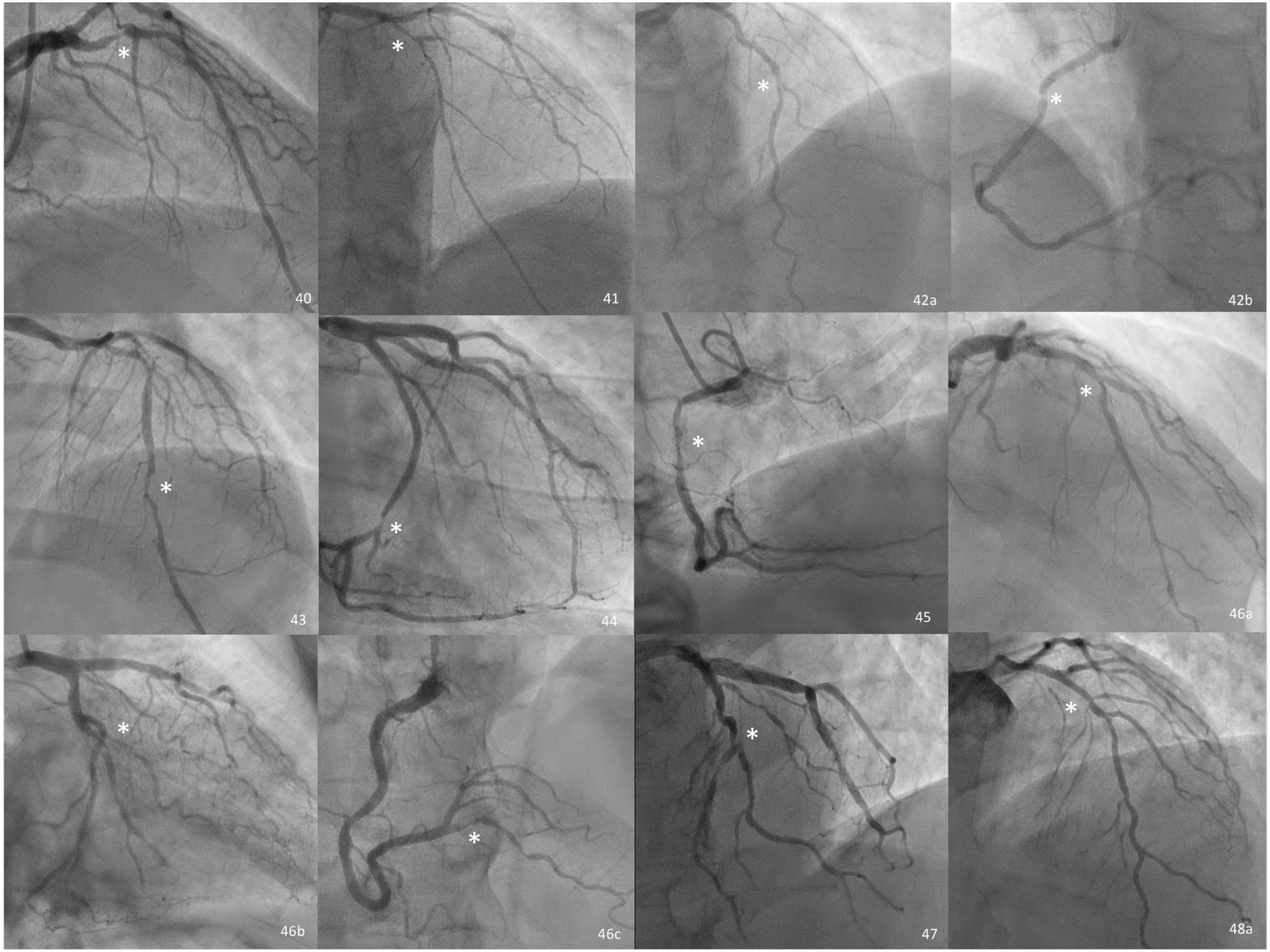
40
41
42a
42b
43
44
45
46a
46b
46c
47
48a

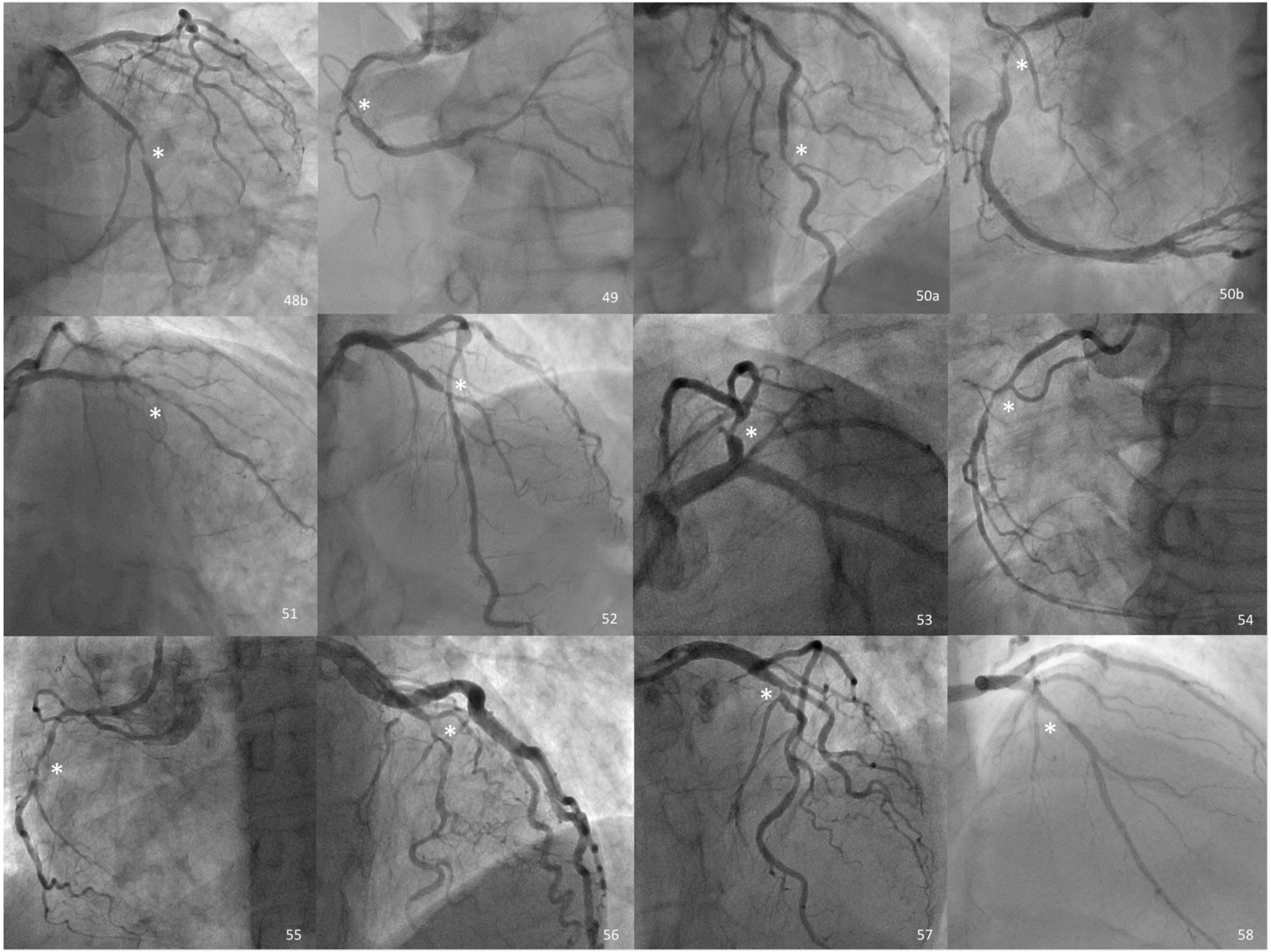
48b
49
50a
50b
51
52
53
54
55
56
57
58

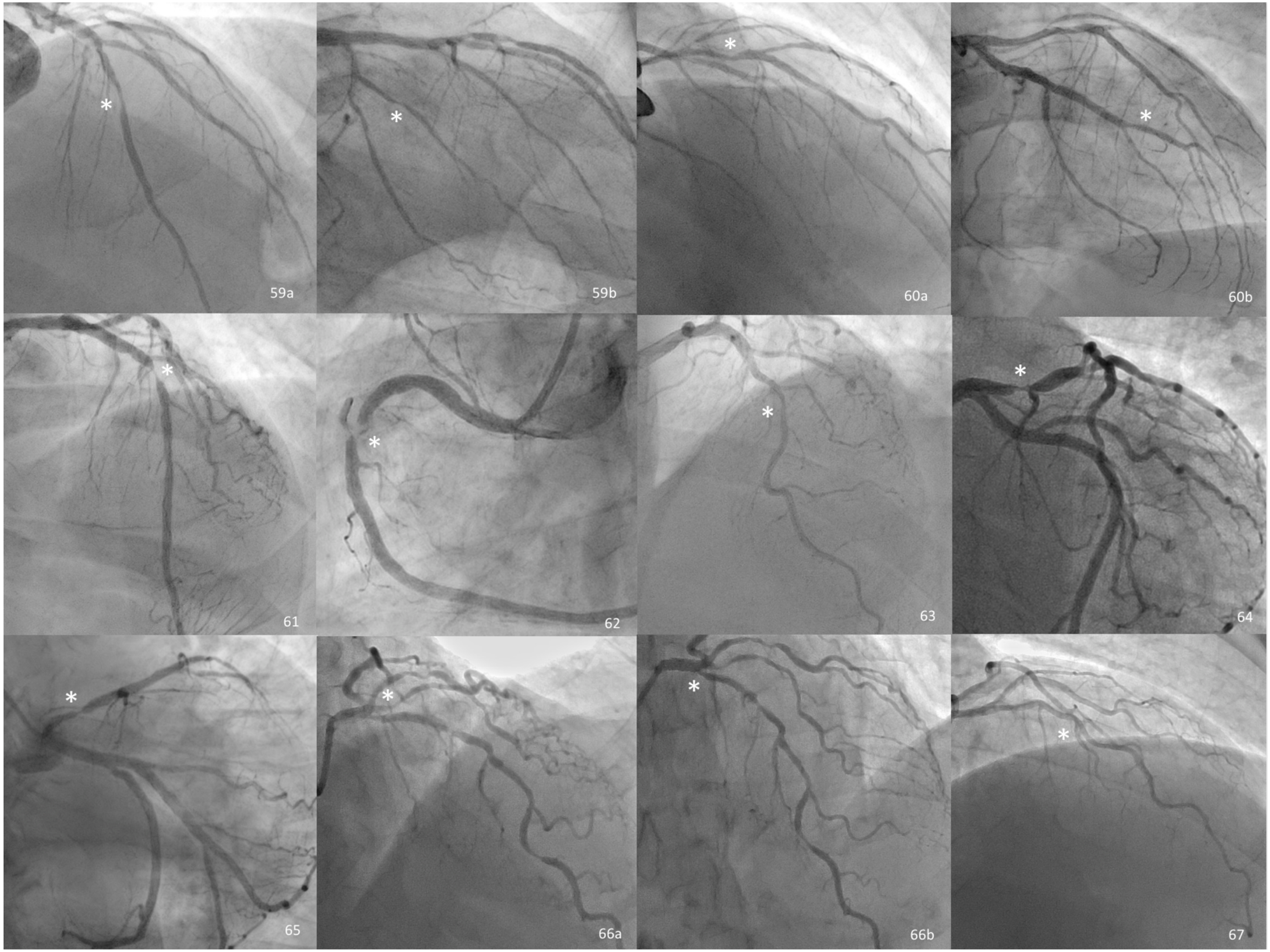
59a
59b
60a
60b
61
62
63
64
65
66a
66b
67

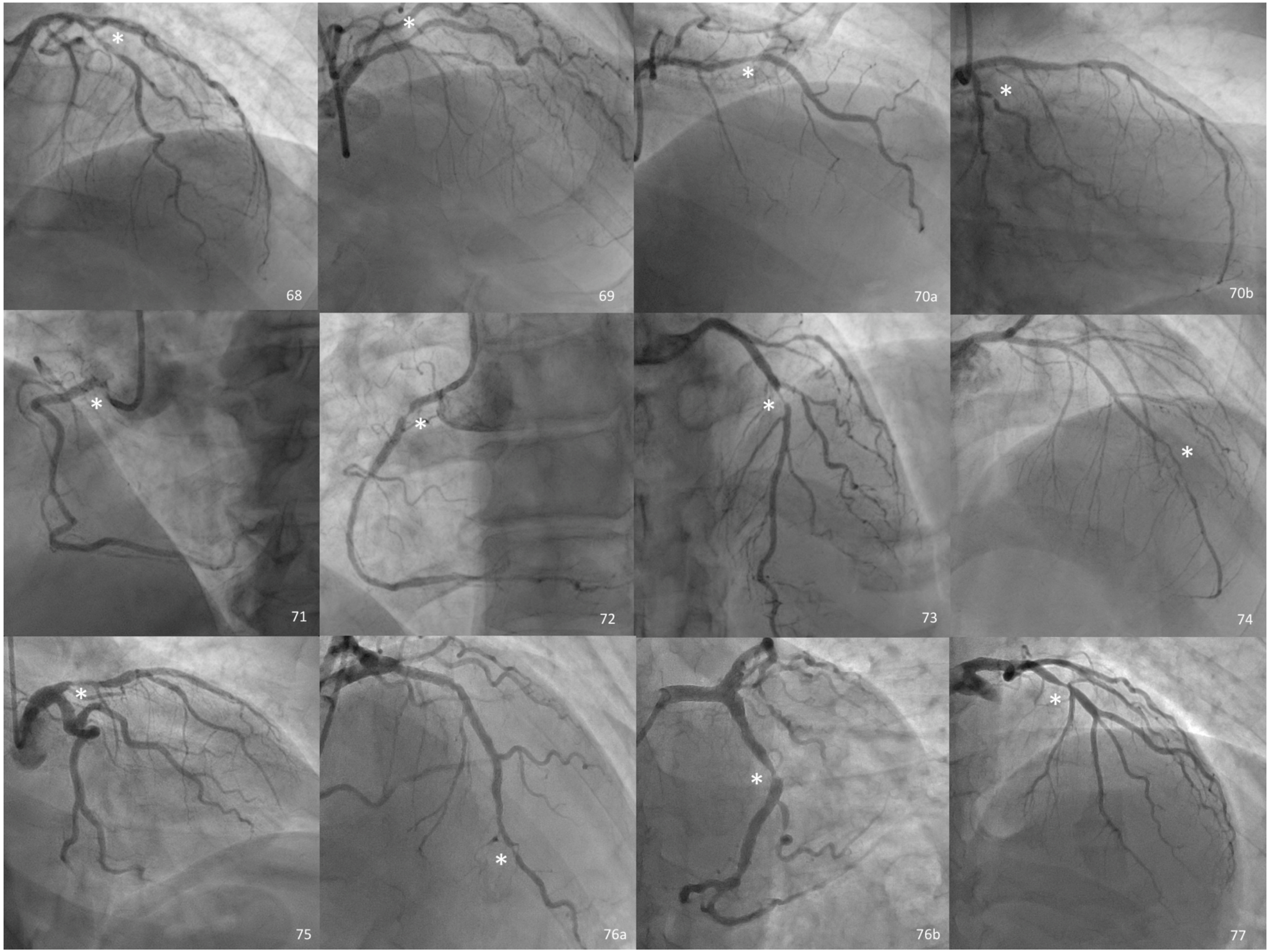
68
69
70a
70b
71
72
73
74
75
76a
76b
77

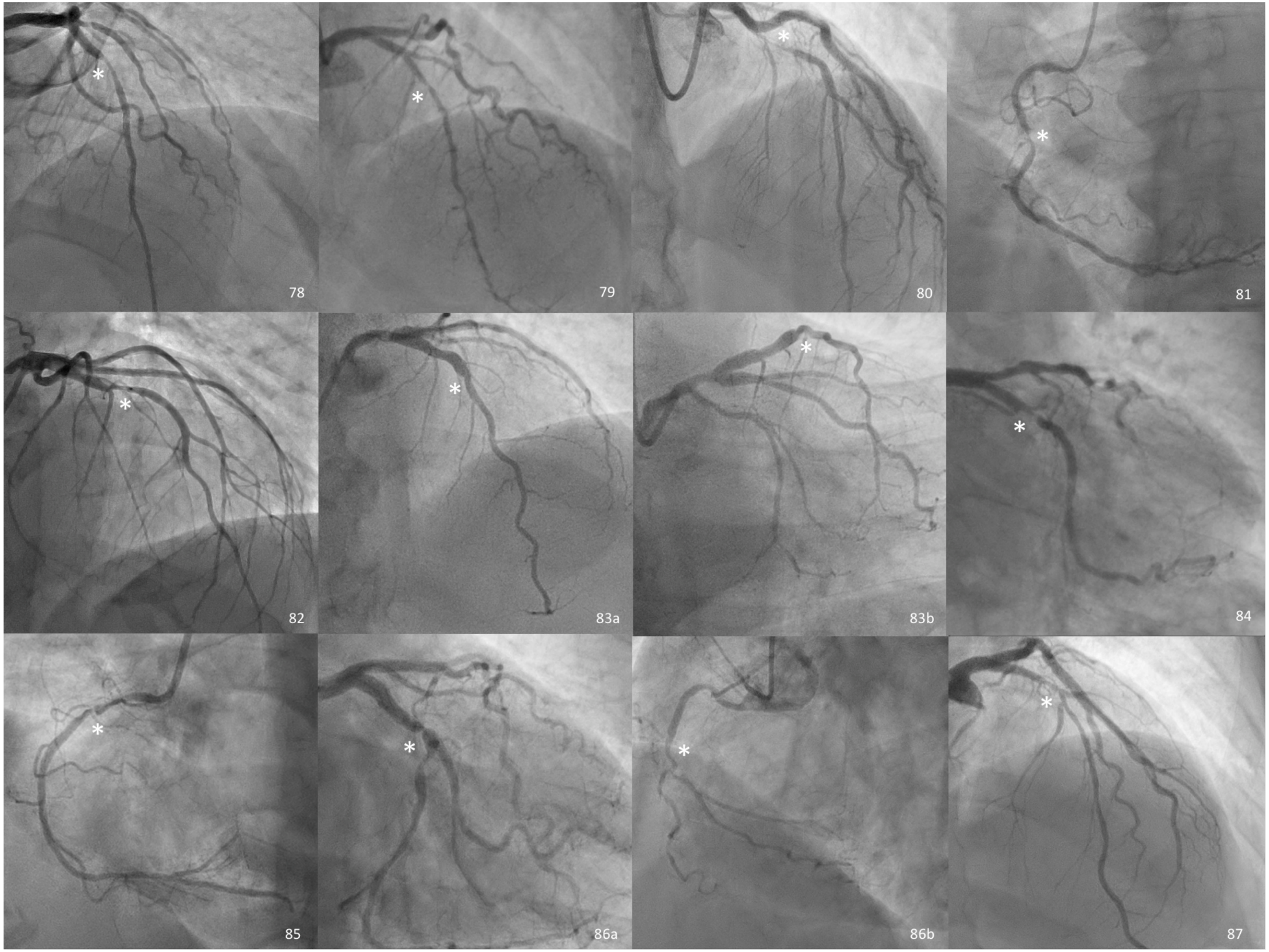
78
79
80
81
82
83a
83b
84
85
86a
86b
87

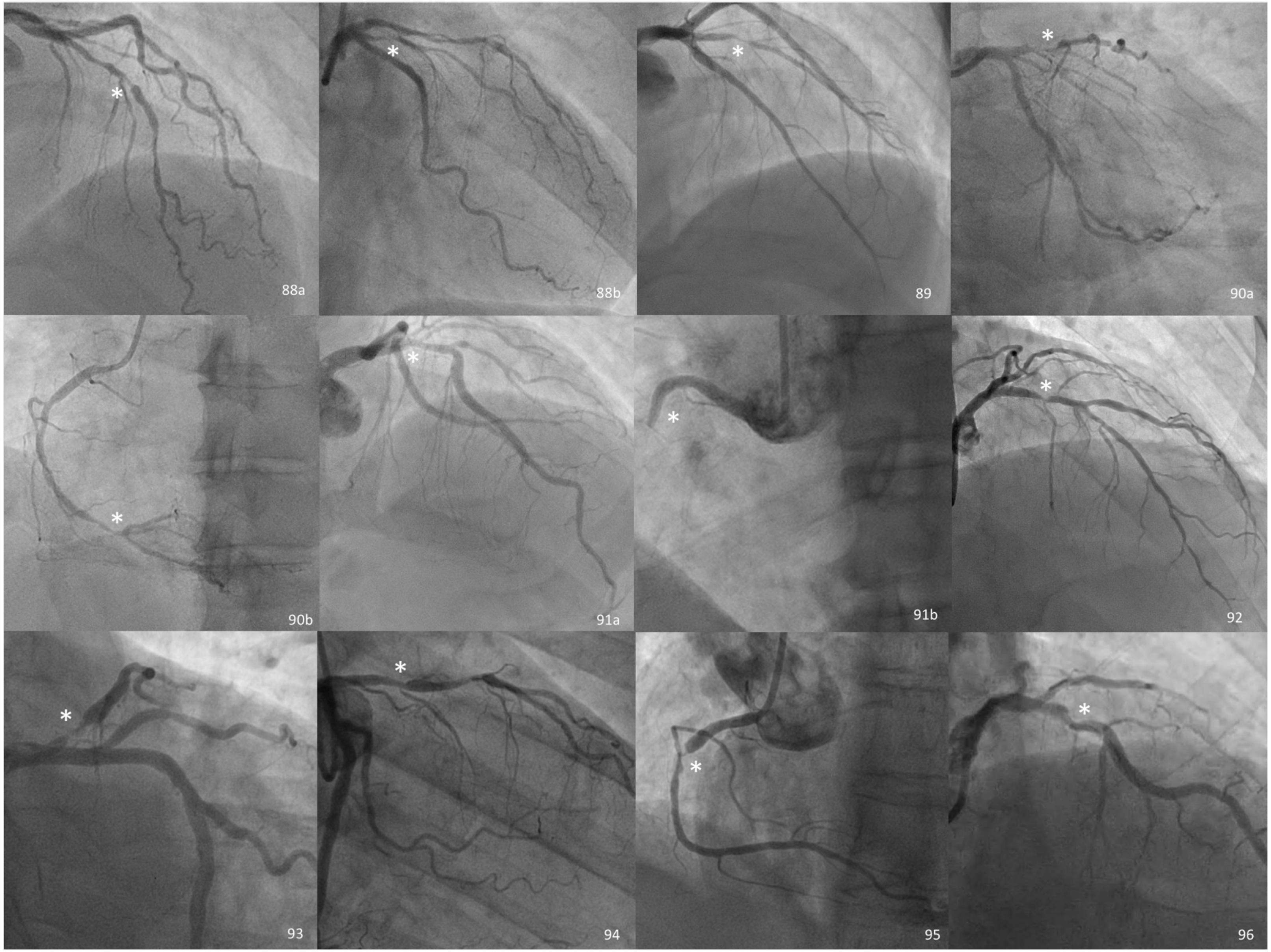
88a
88b
89
90a
90b
91a
91b
92
93
94
95
96

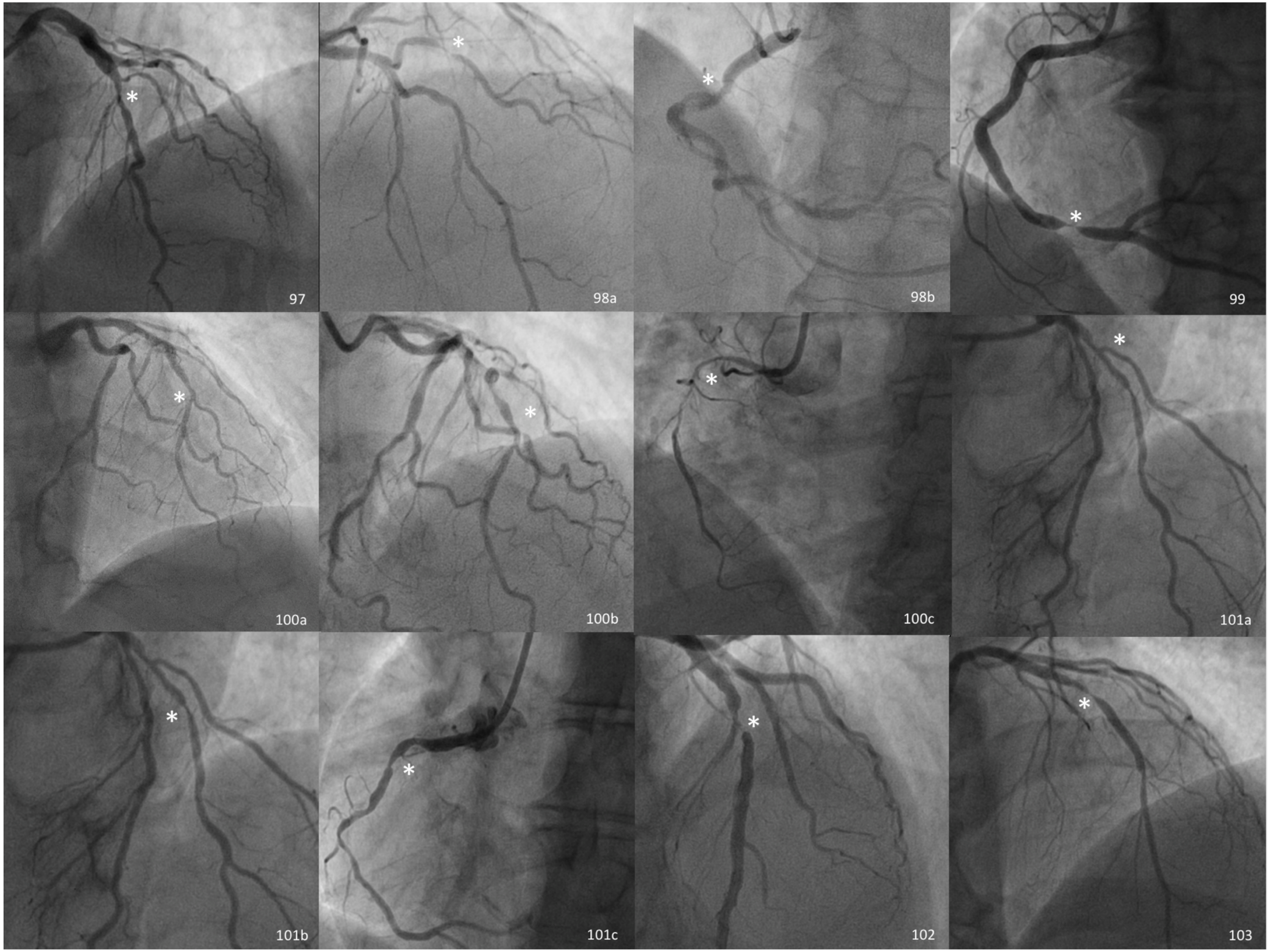
97
98a
98b
99
100a
100b
100c
101a
101b
101c
102
103

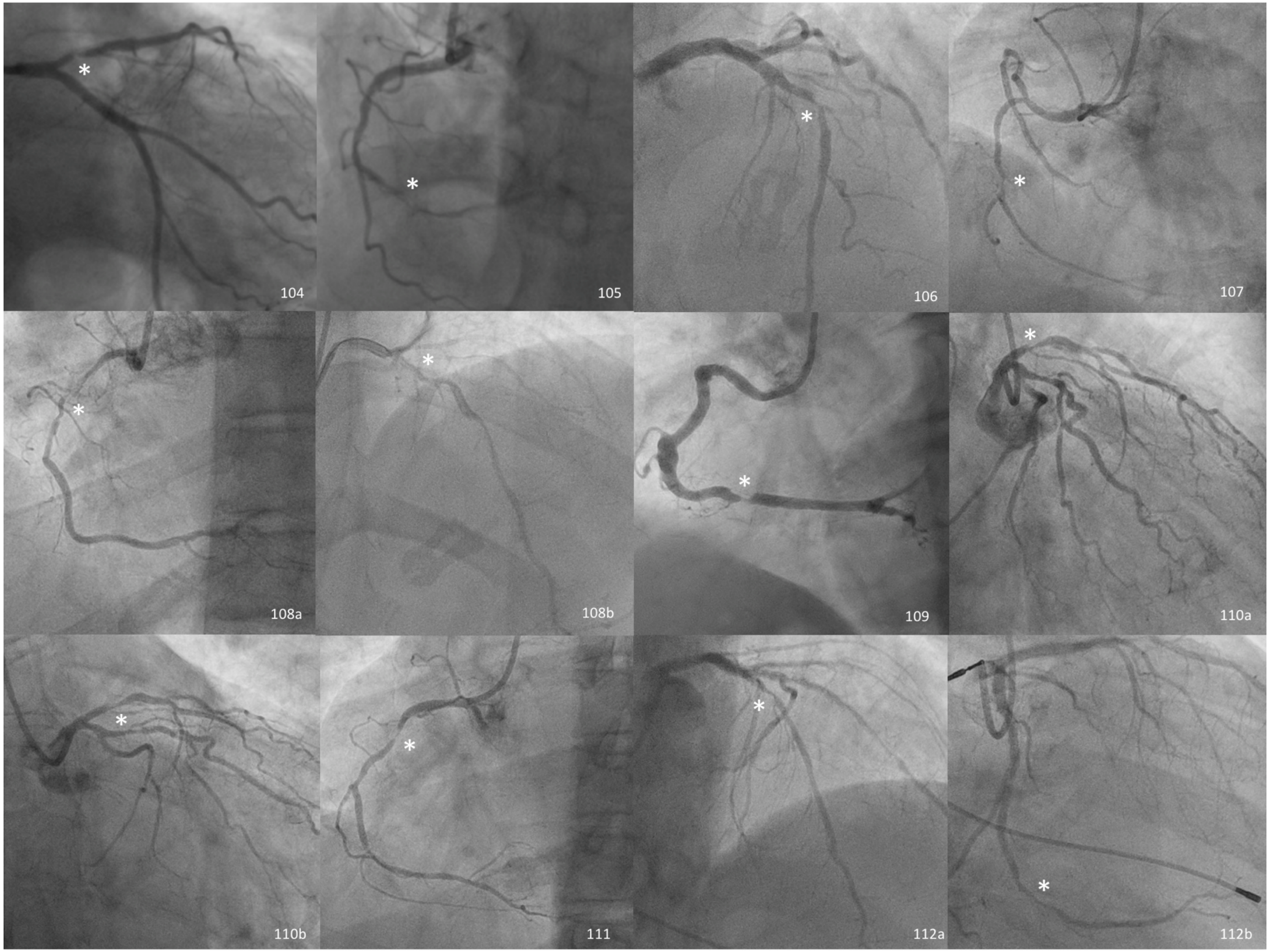
104
105
106
107
108a
108b
109
110a
110b
111
112a
112b

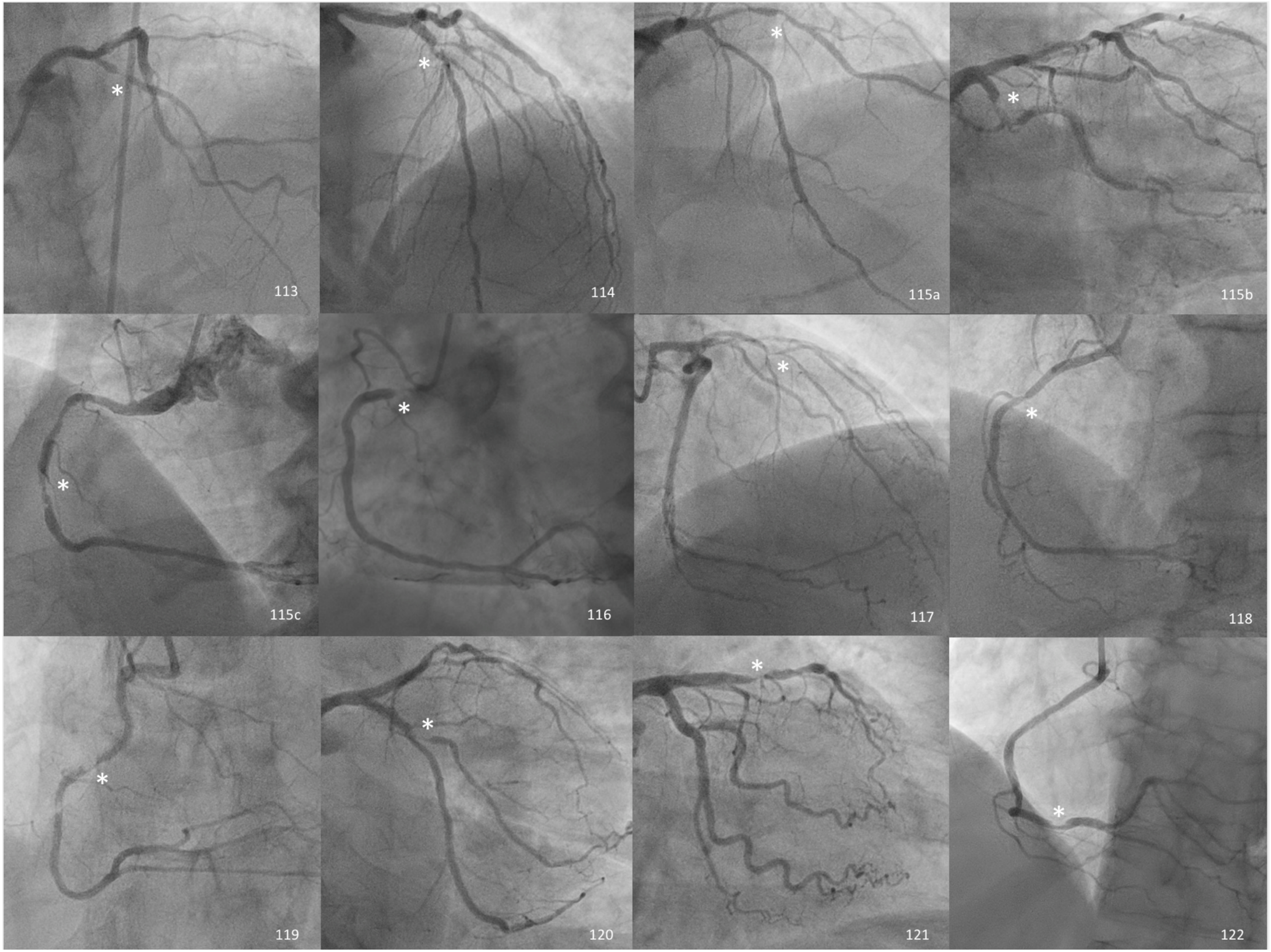
113
114
115a
115b
115c
116
117
118
119
120
121
122

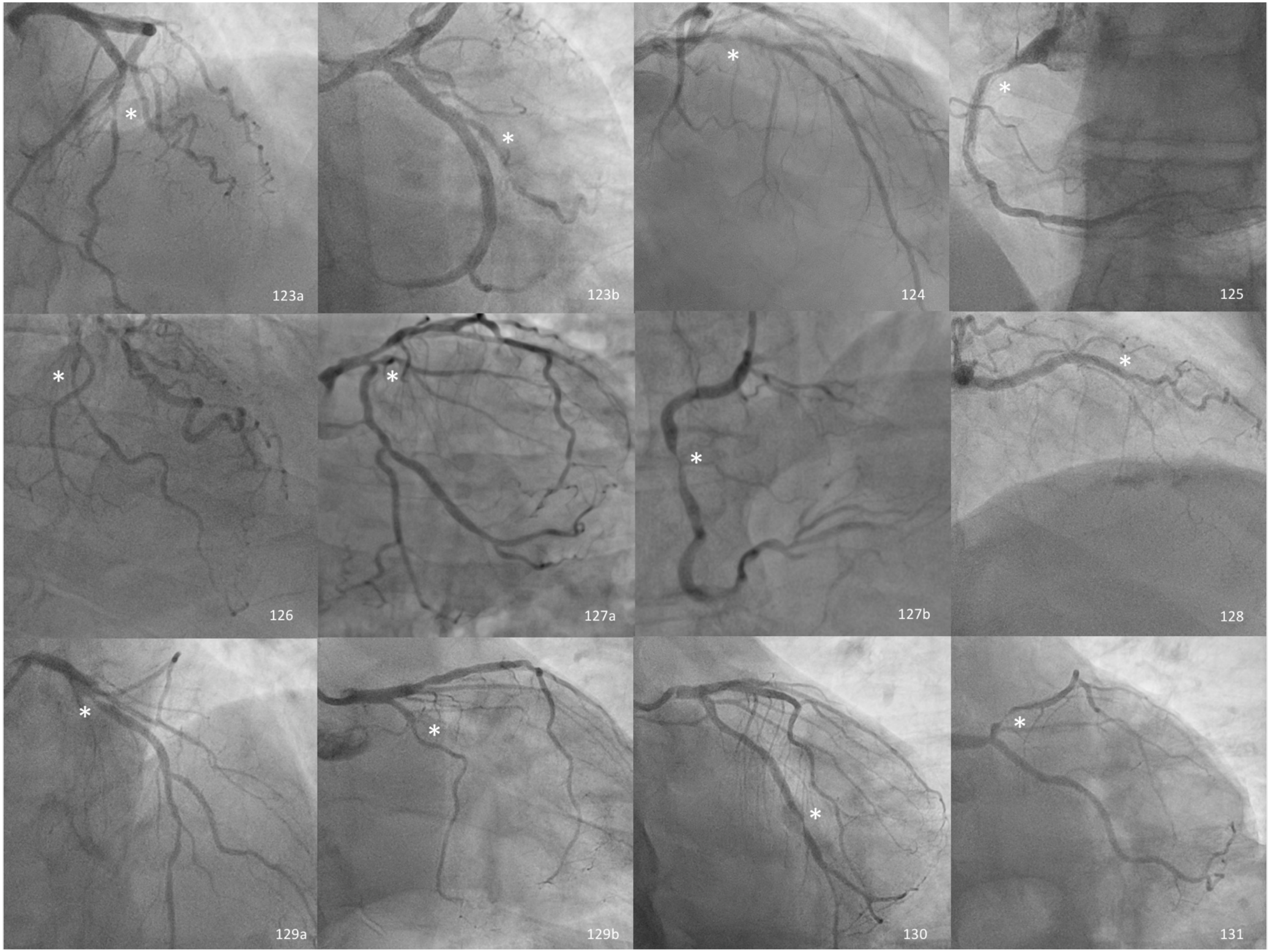
123a
123b
124
125
126
127a
127b
128
129a
129b
130
131

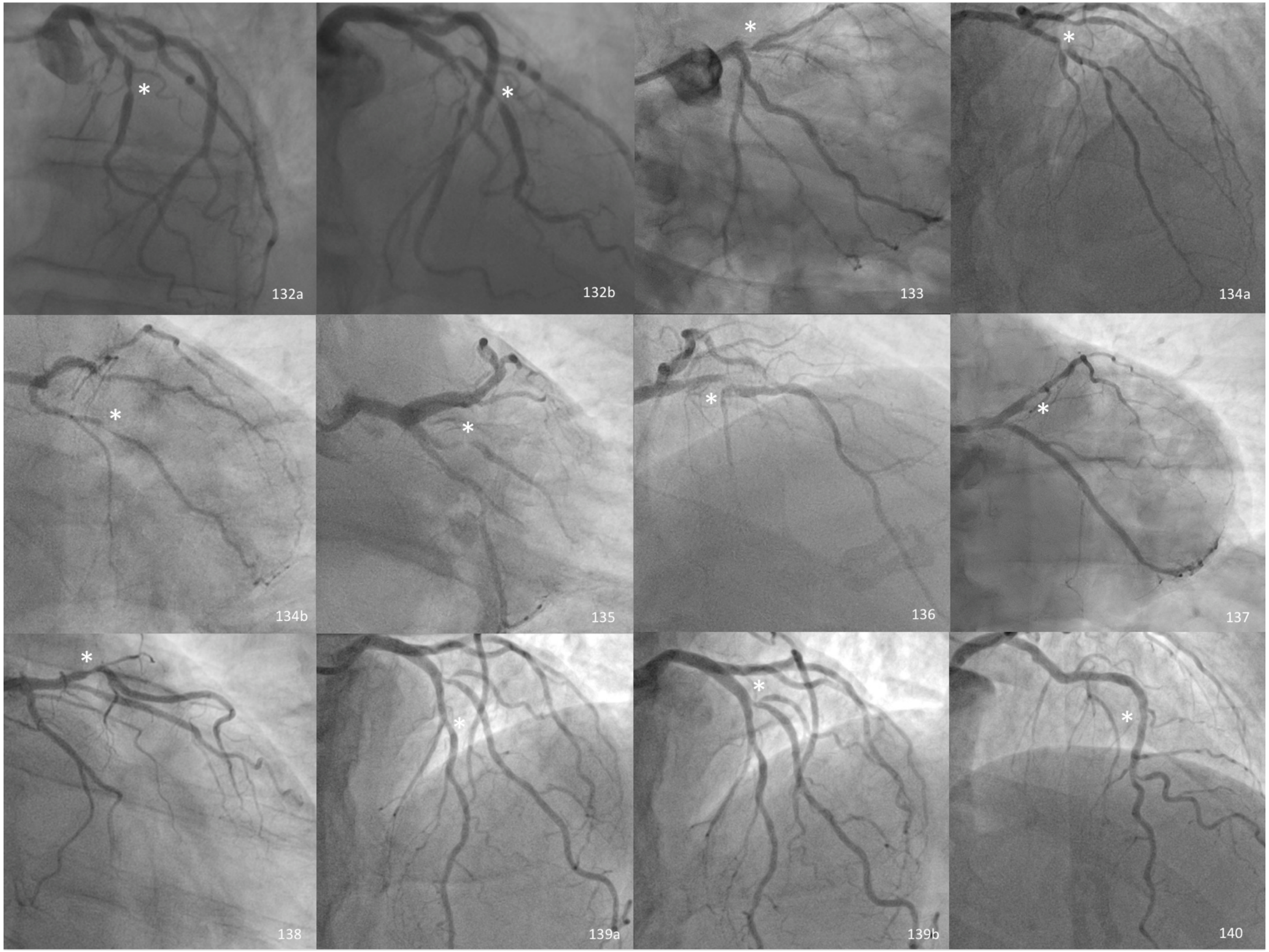
132a
132b
133
134a
134b
135
136
137
138
139a
139b
140

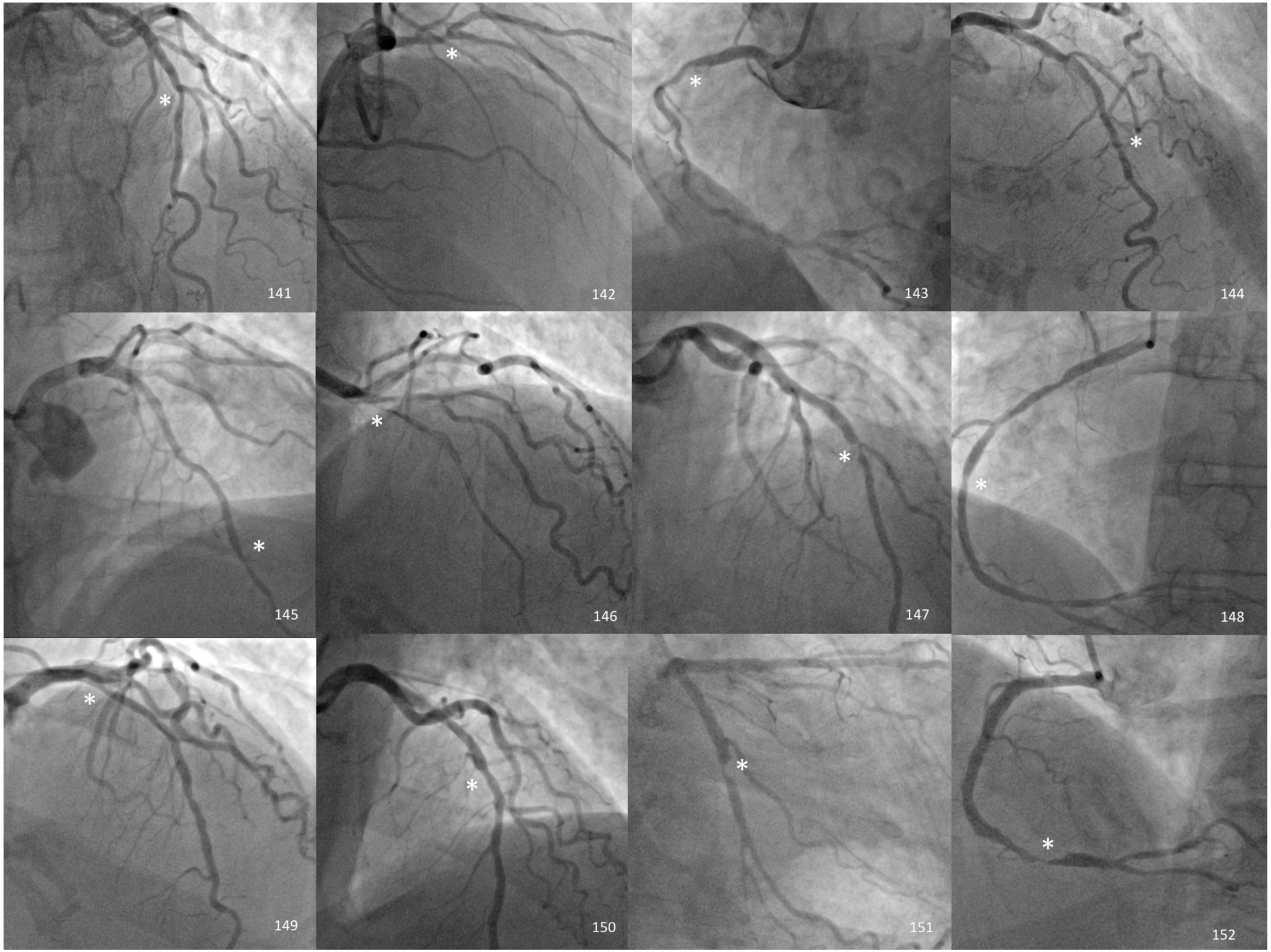
141
142
143
144
145
146
147
148
149
150
151
152

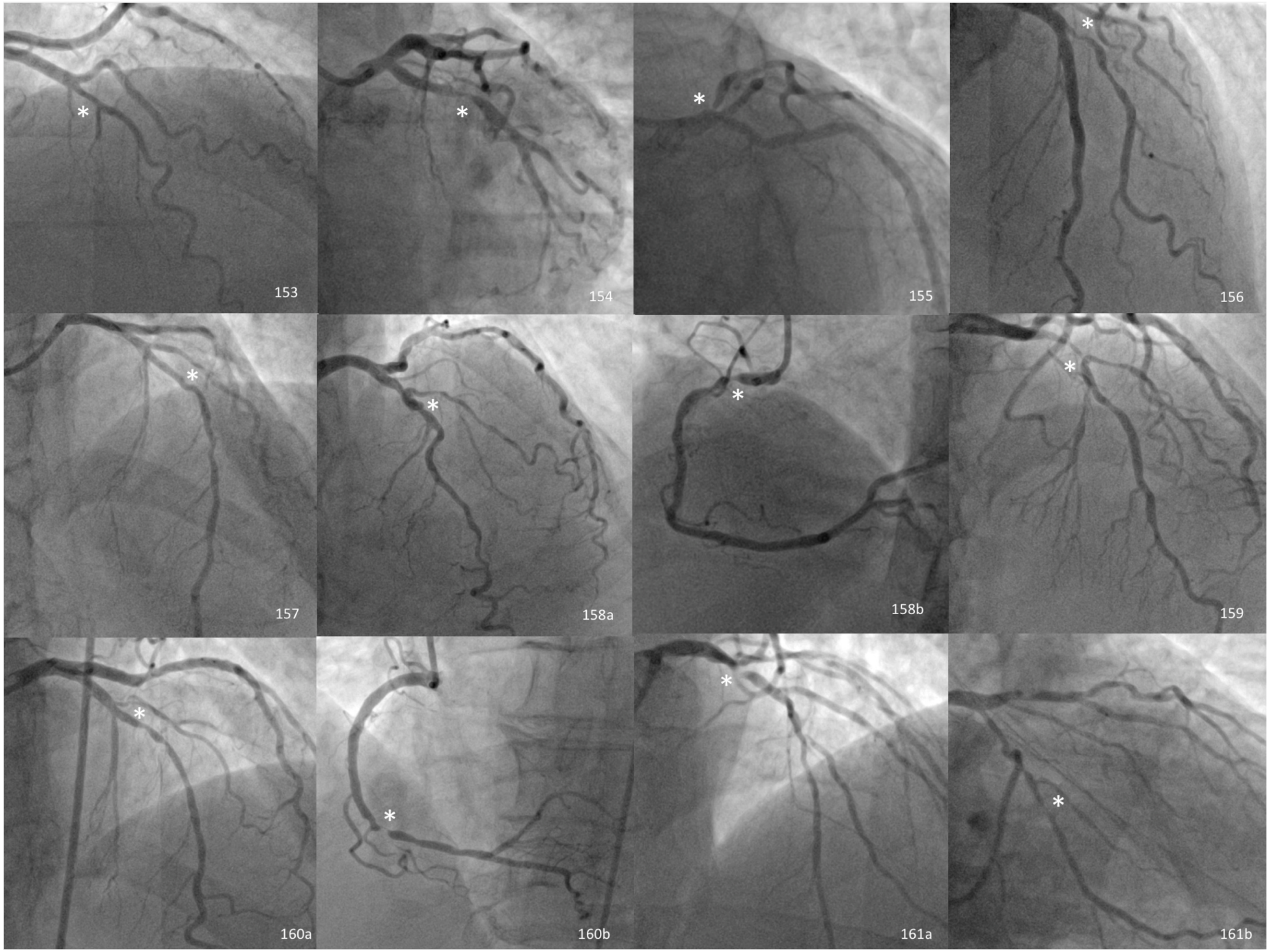
153
154
155
156
157
158a
158b
159
160a
160b
161a
161b

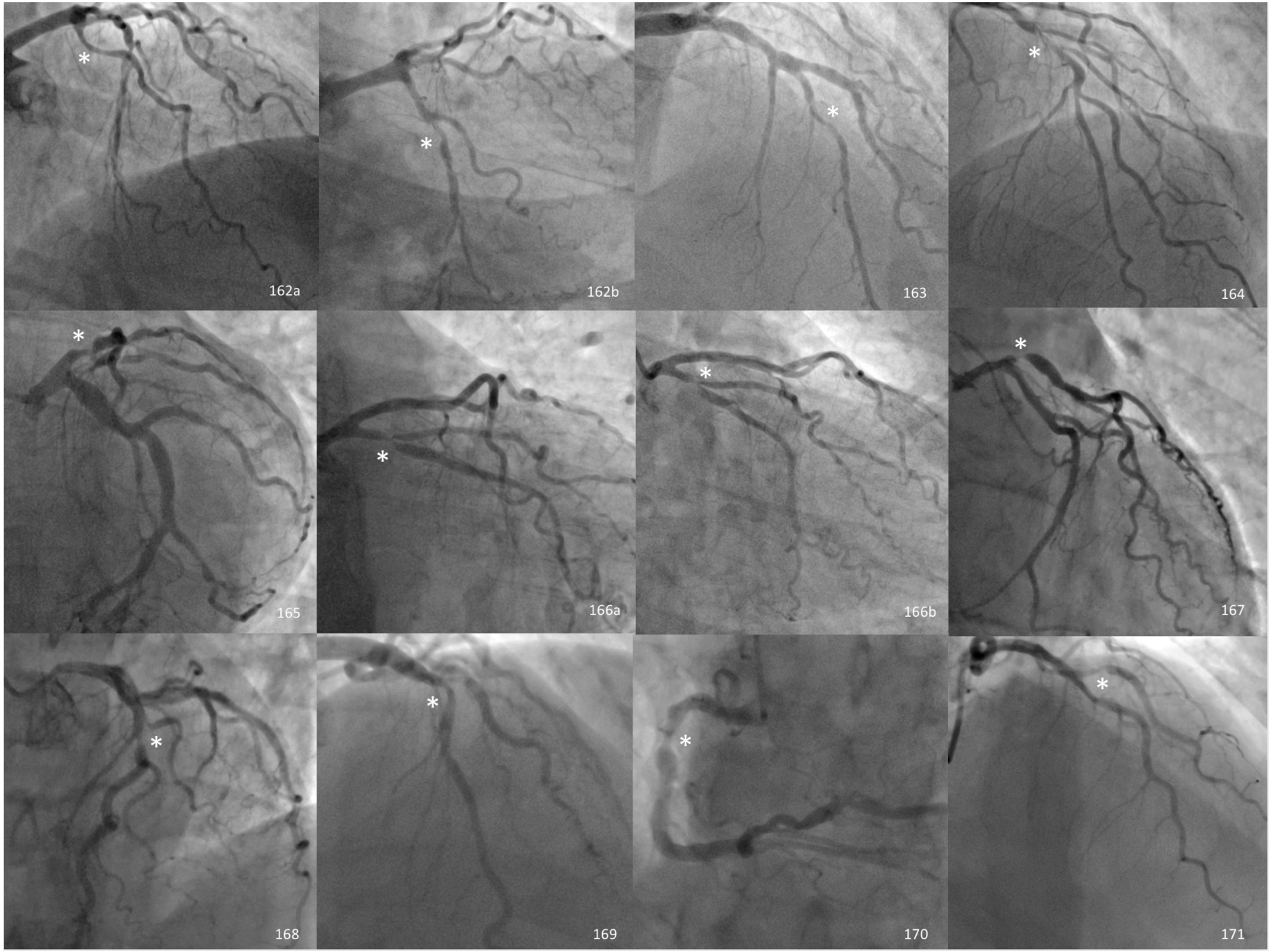
162a
162b
163
164
165
166a
166b
167
168
169
170
171

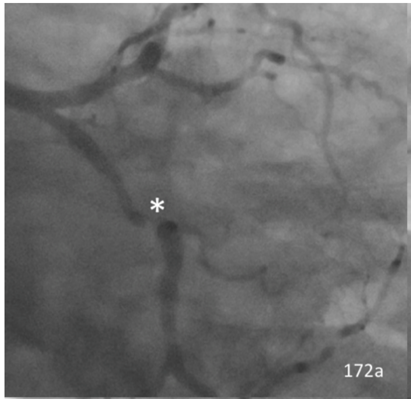

172a

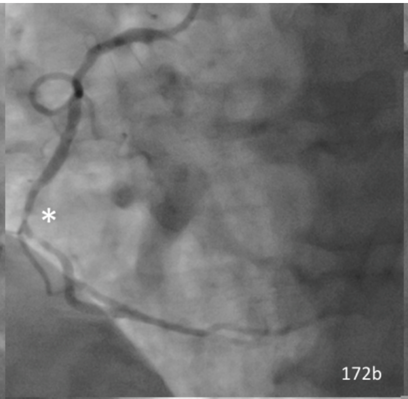

172b

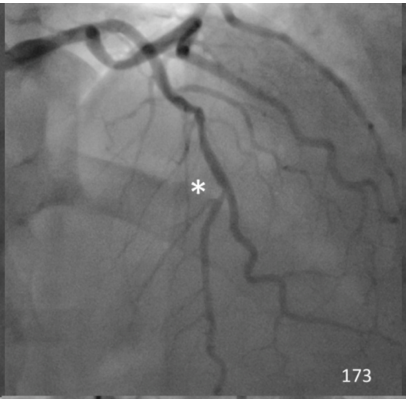

173

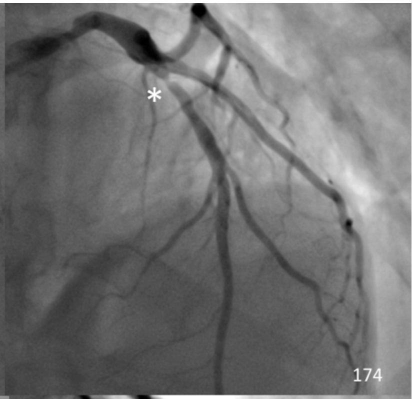

174

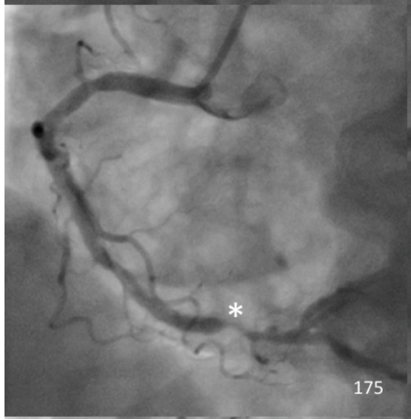

175

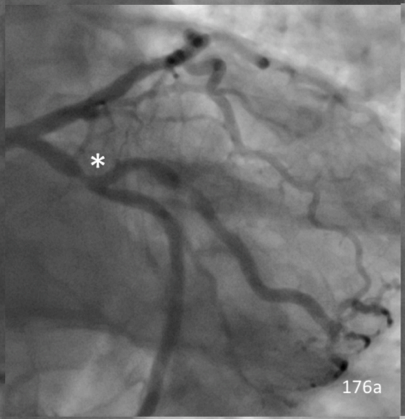

176a

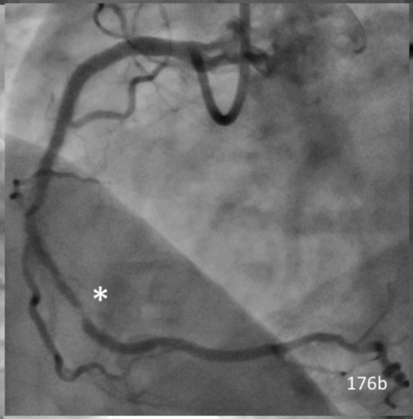

176b

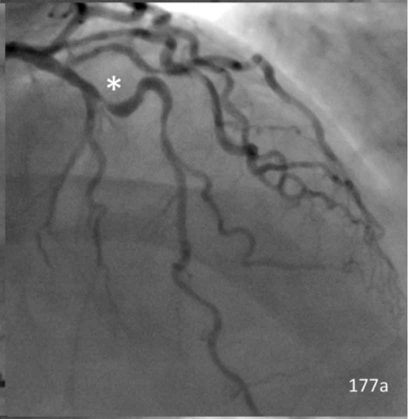

177a

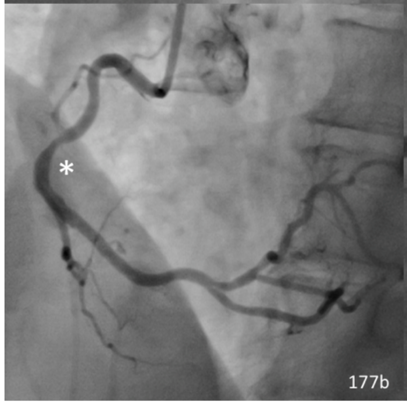

177b

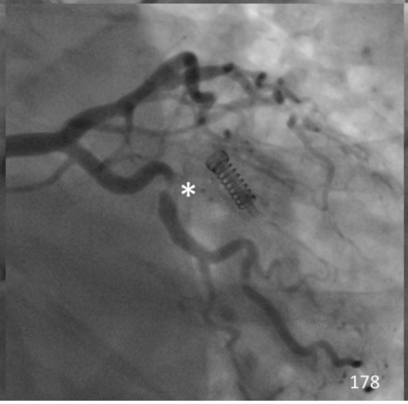

178

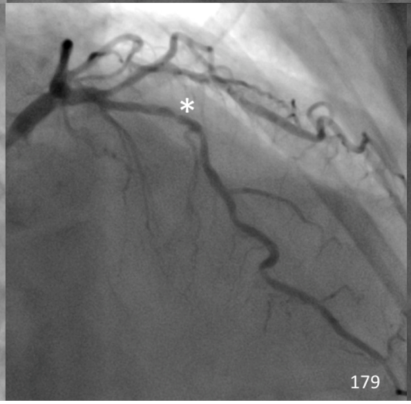

179

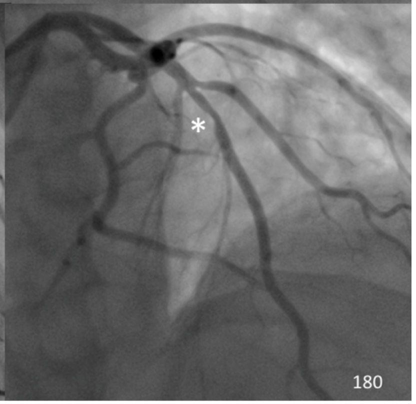

180

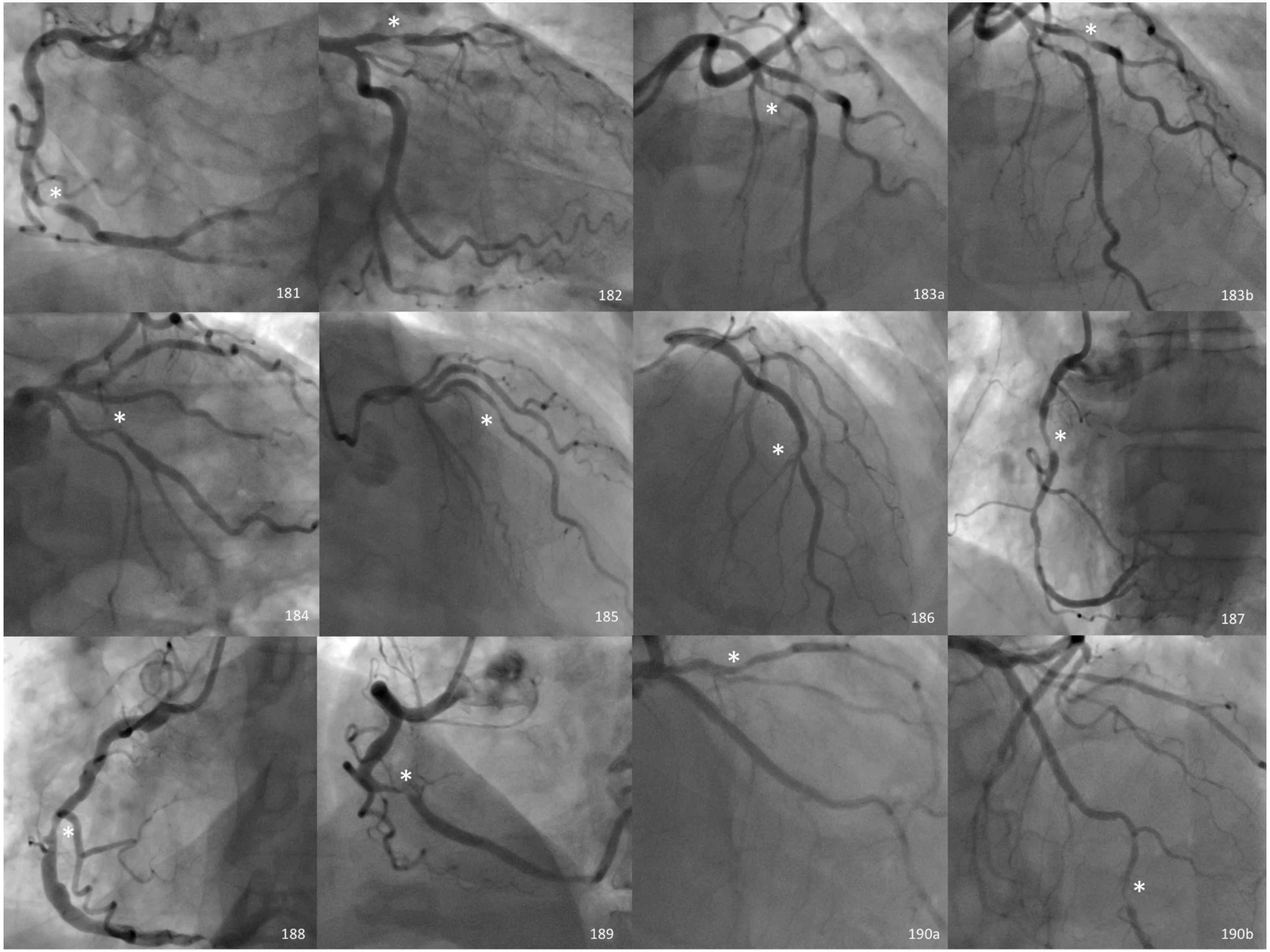
181
182
183a
183b
184
185
186
187
188
189
190a
190b

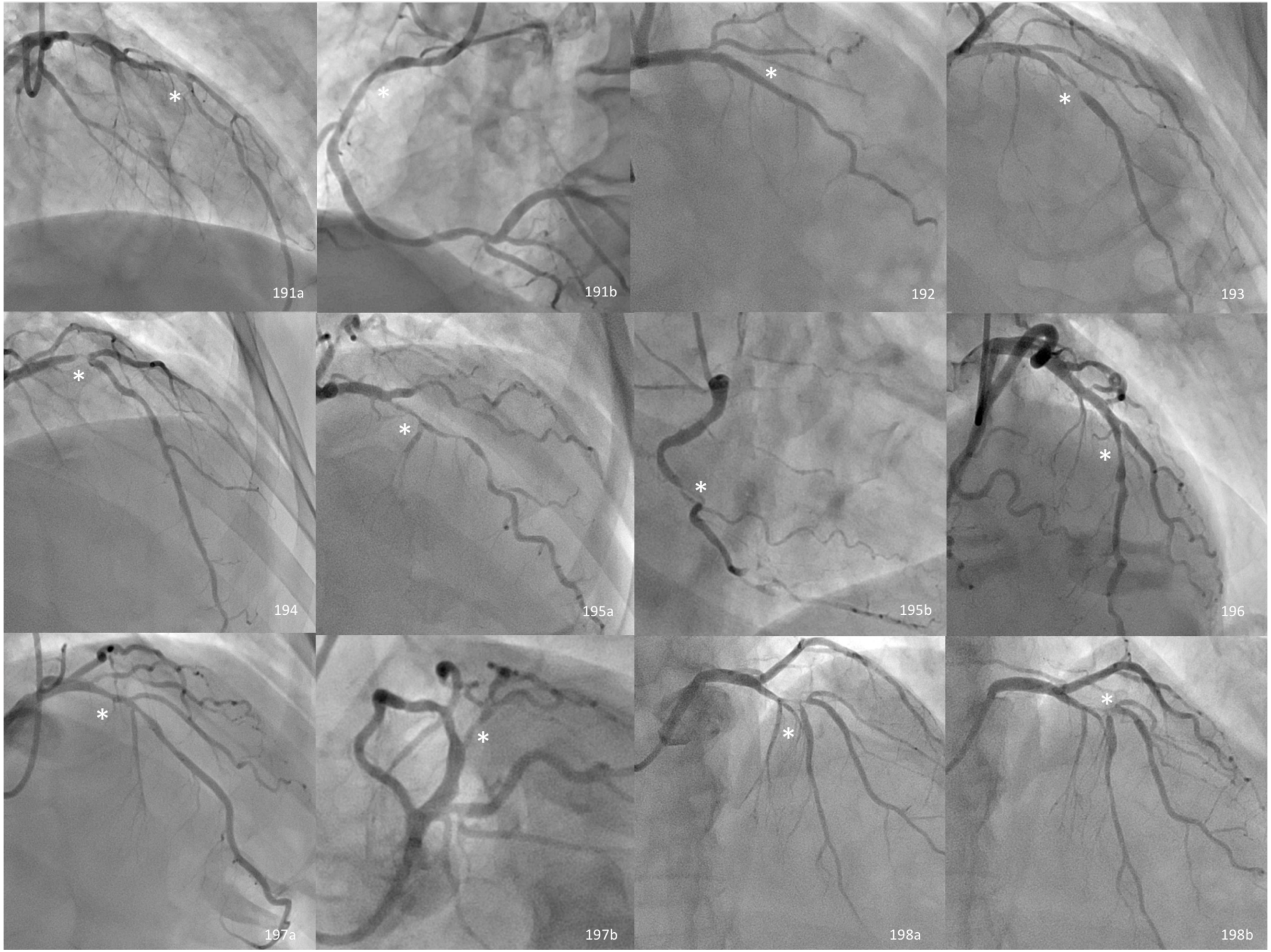
191a
191b
192
193
194
195a
195b
196
197a
197b
198a
198b

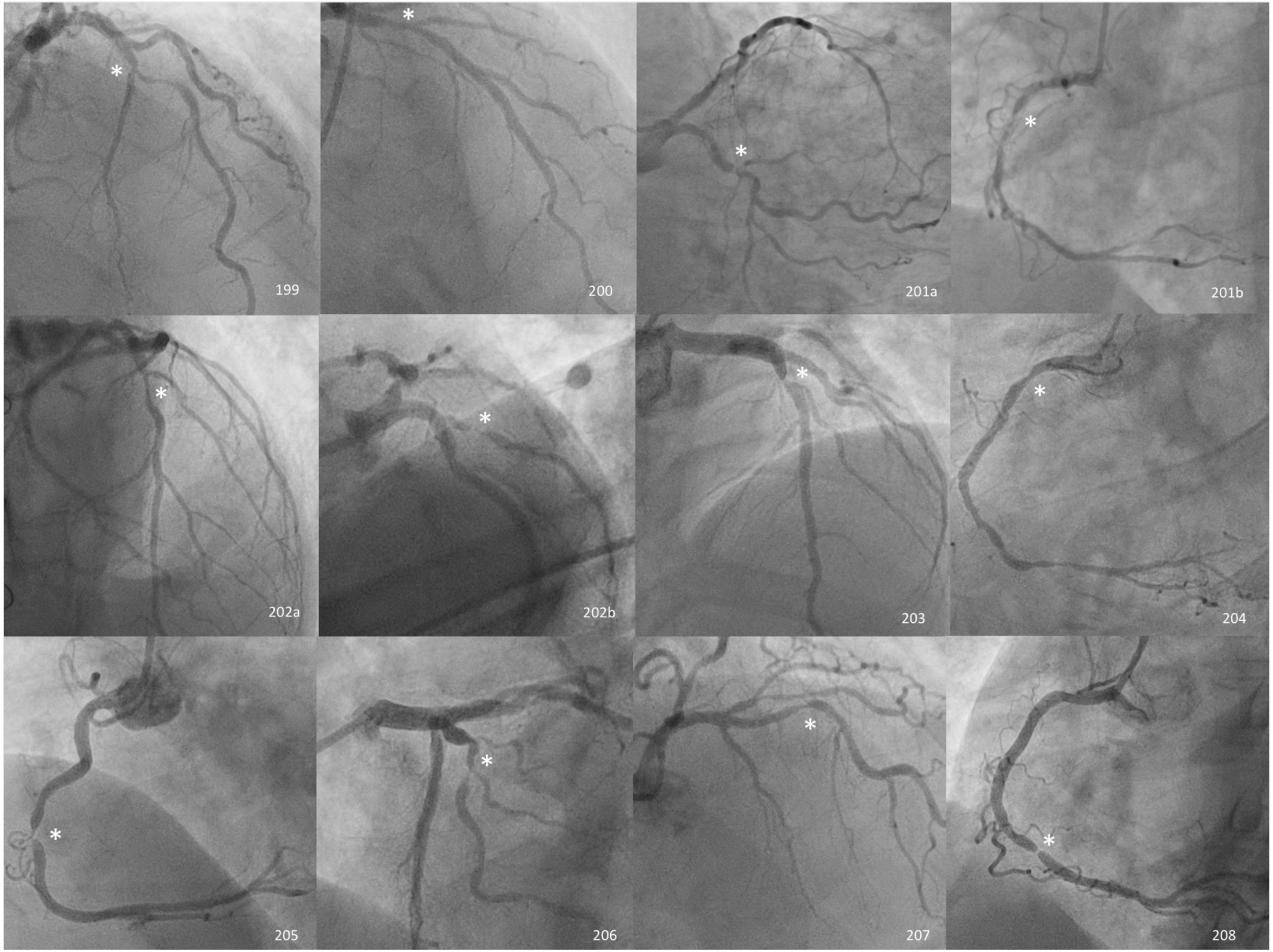
199
200
201a
201b
202a
202b
203
204
205
206
207
208

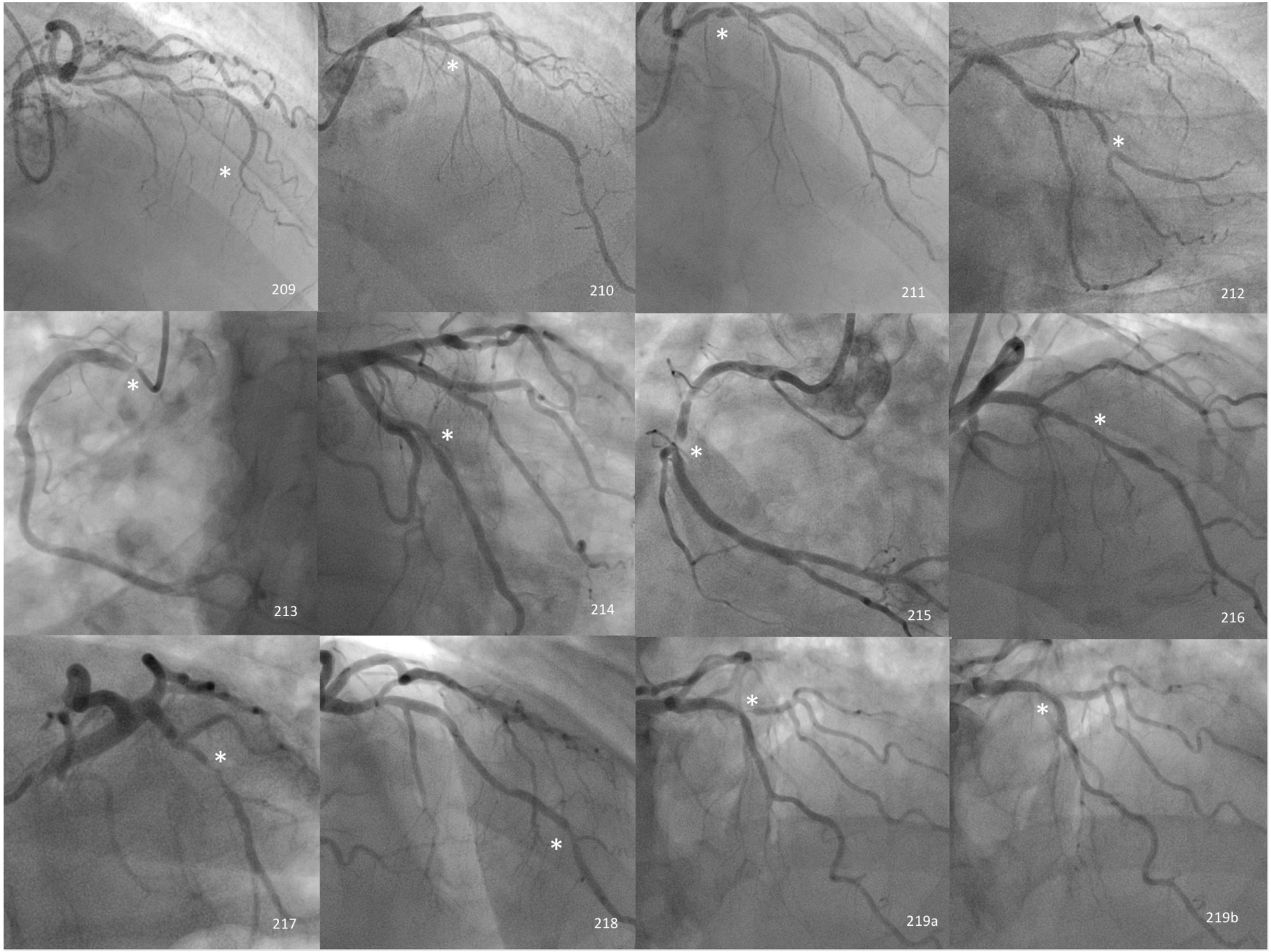
209
210
211
212
213
214
215
216
217
218
219a
219b

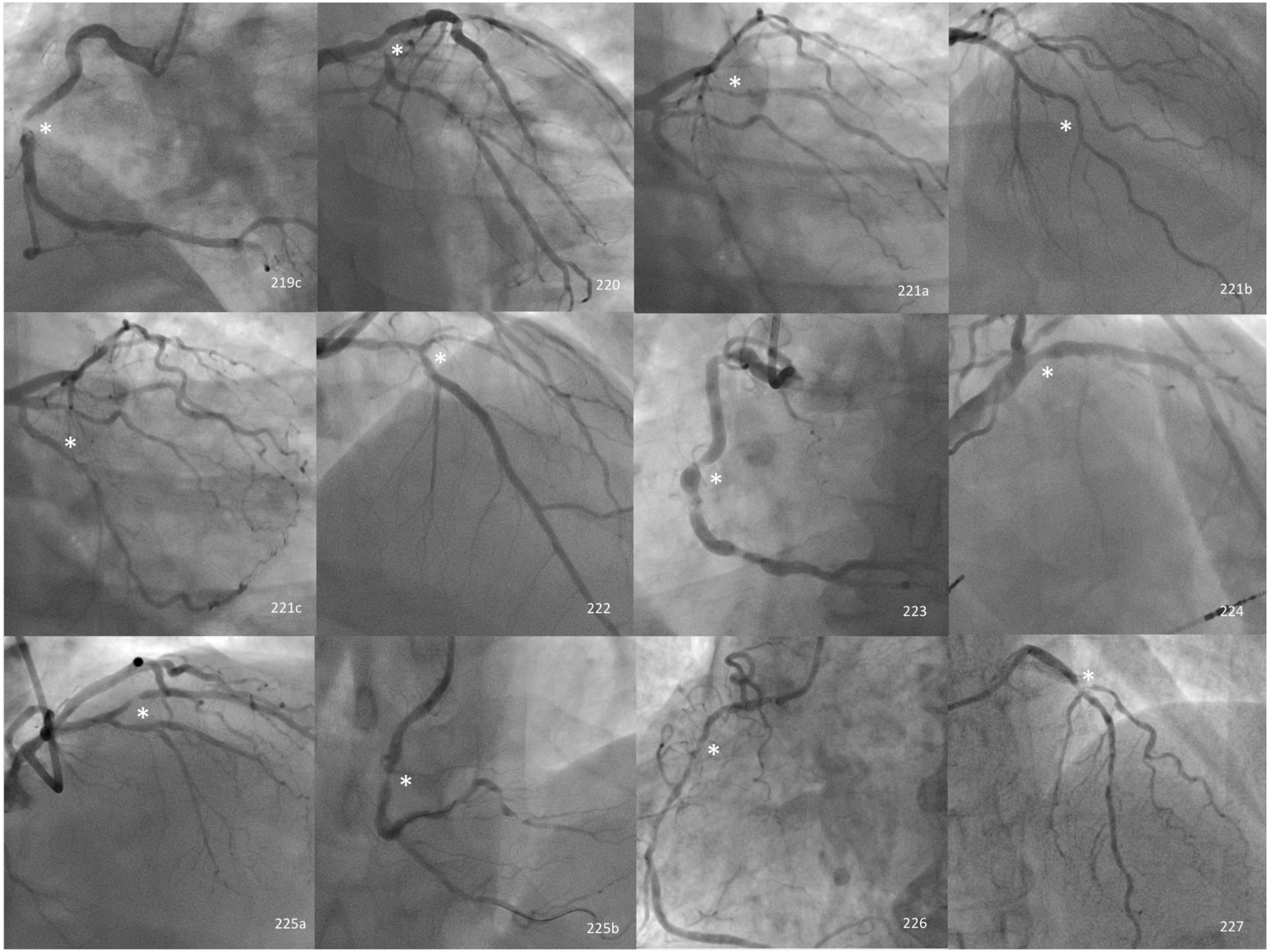
219c
220
221a
221b
221c
222
223
224
225a
225b
226
227

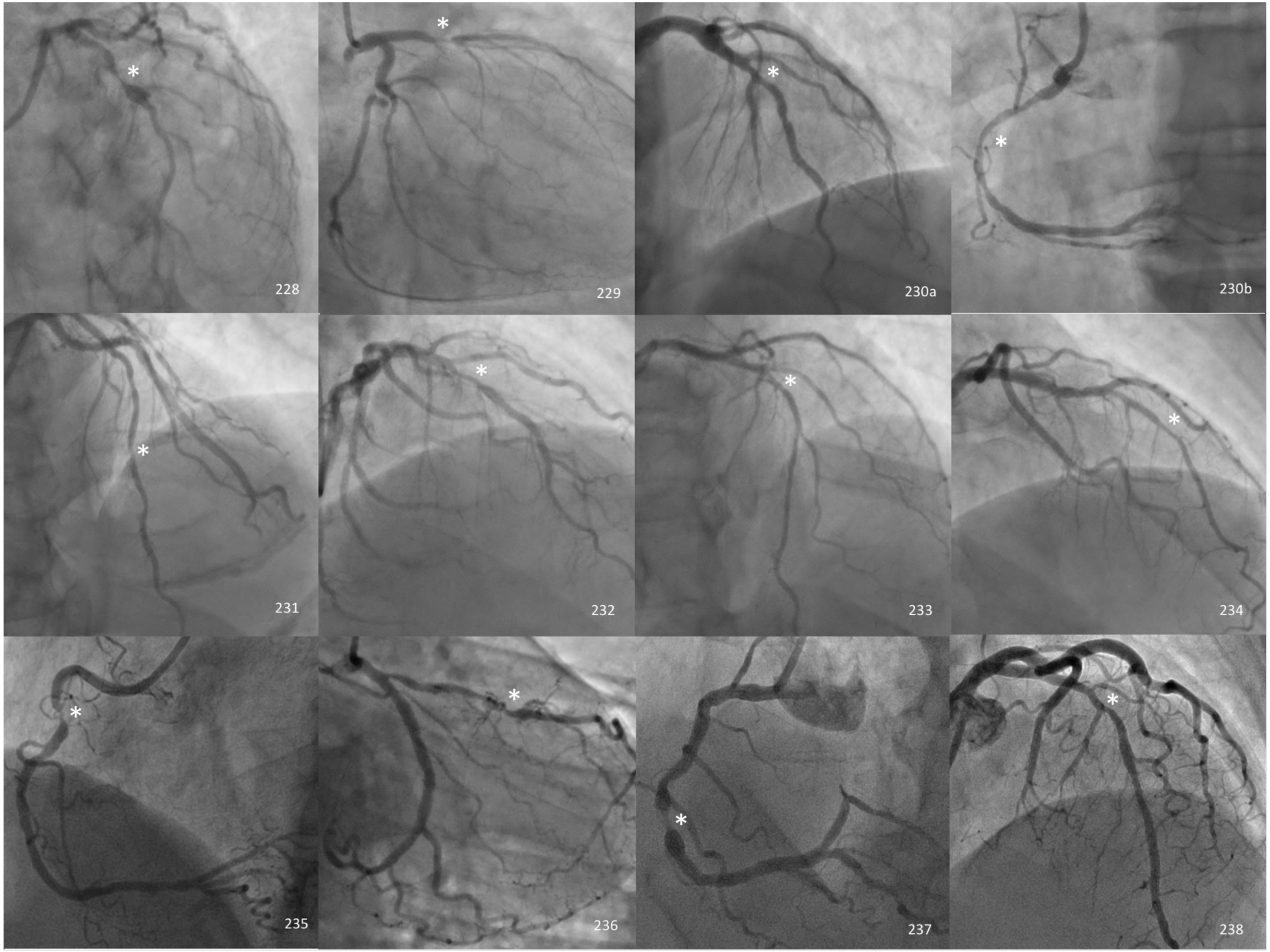
228
229
230a
230b
231
232
233
234
235
236
237
238

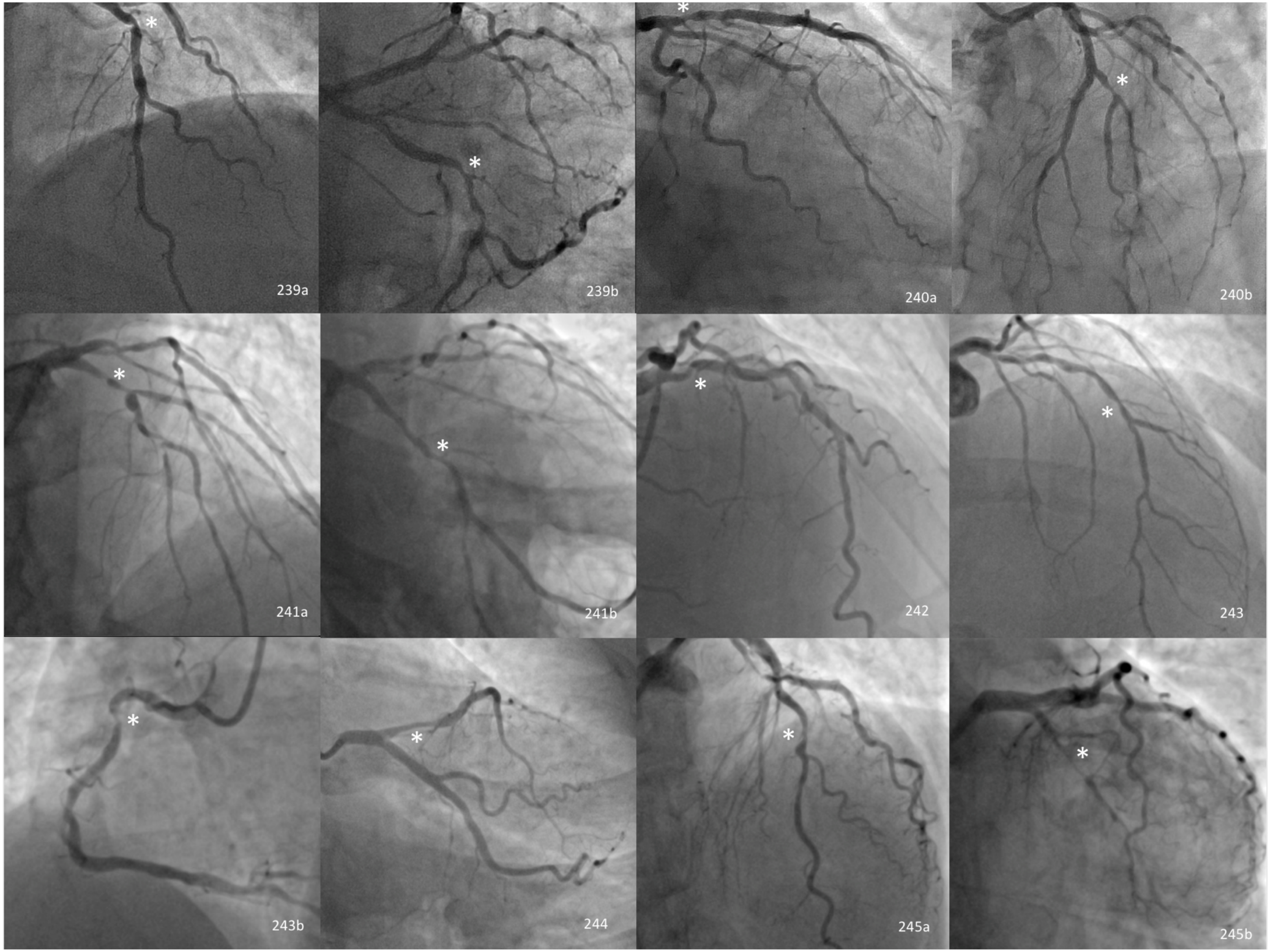
239a
239b
240a
240b
241a
241b
242
243
243b
244
245a
245b

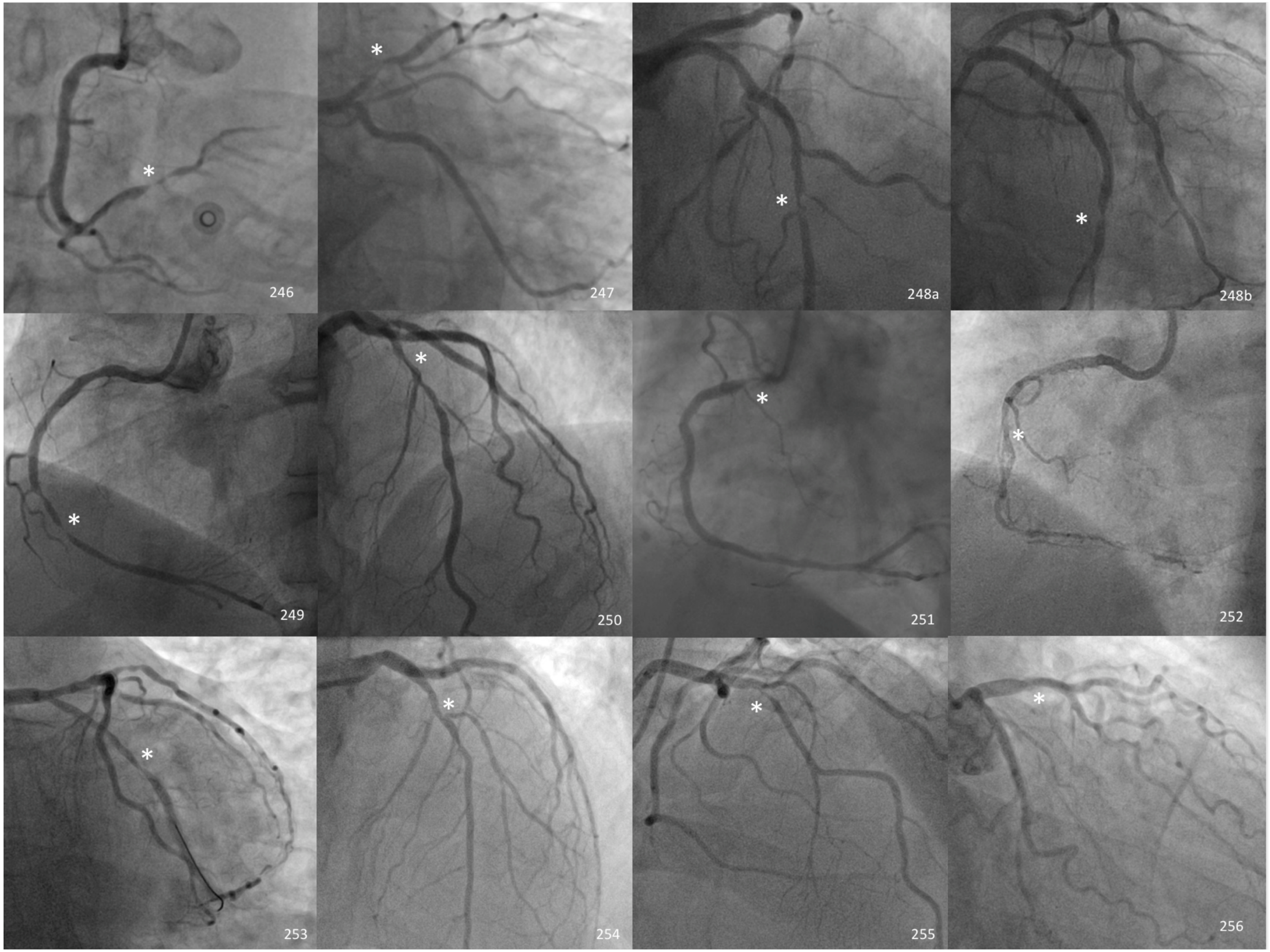
246
247
248a
248b
249
250
251
252
253
254
255
256

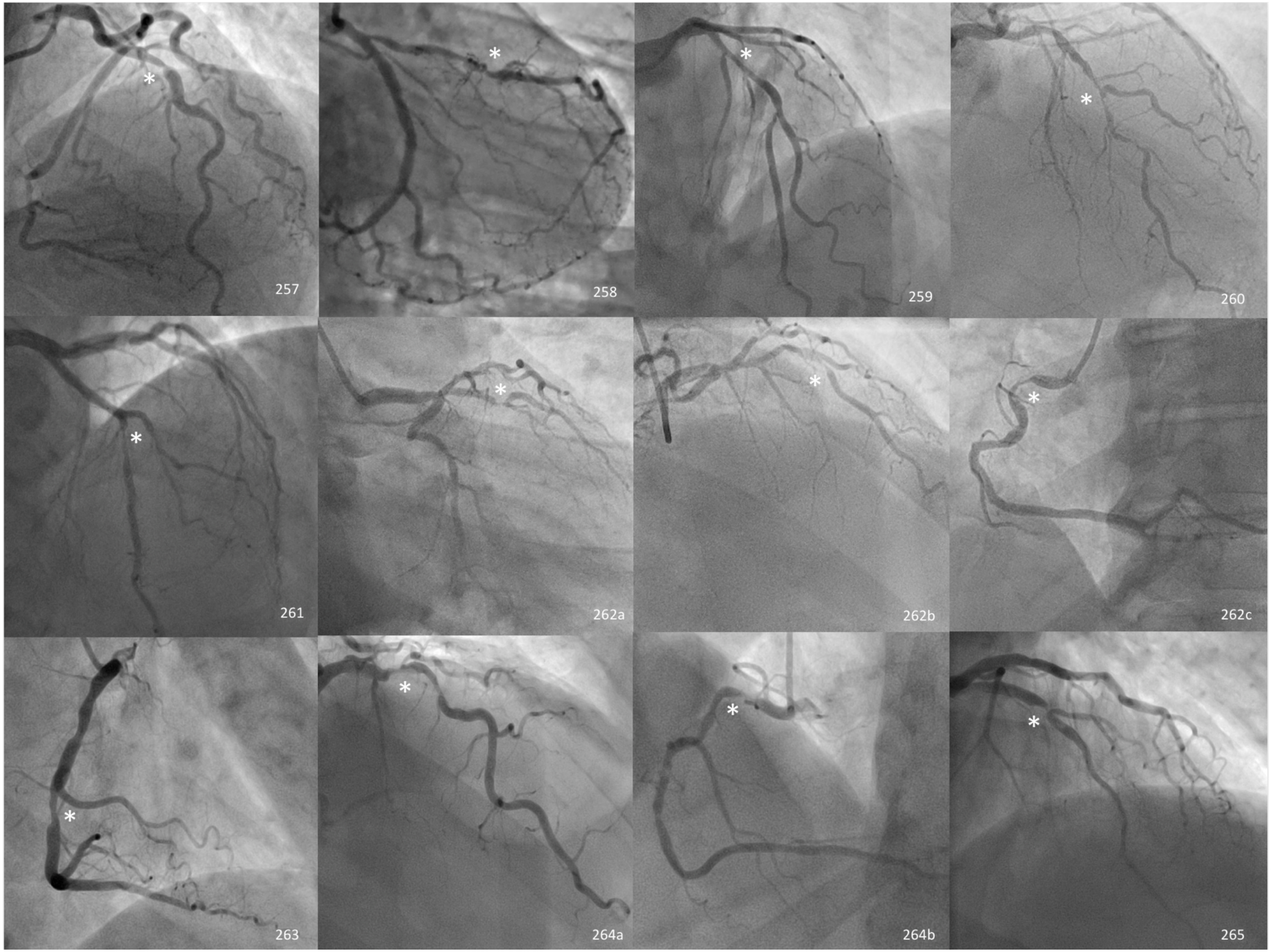
257
258
259
260
261
262a
262b
262c
263
264a
264b
265

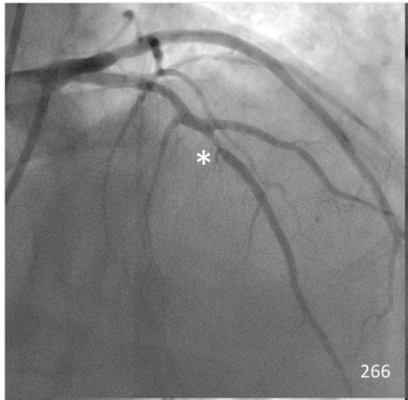
266

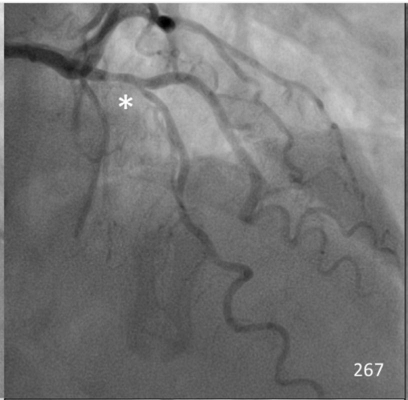
267

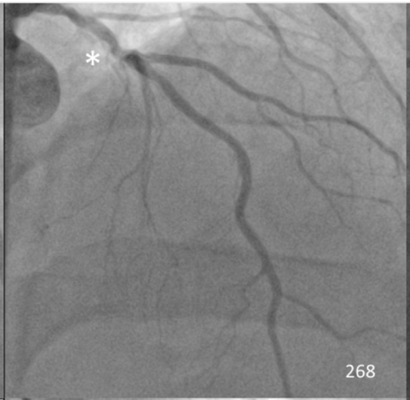
268

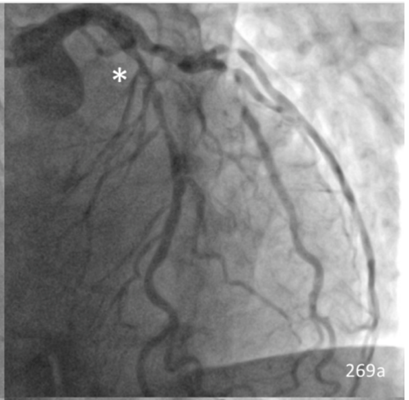
269a

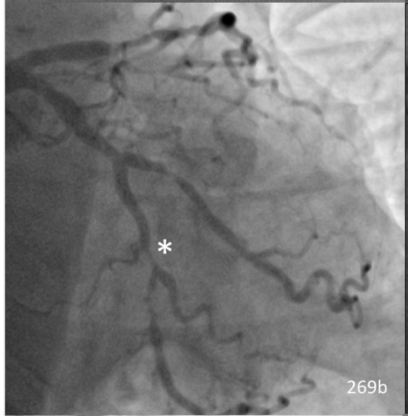
269b

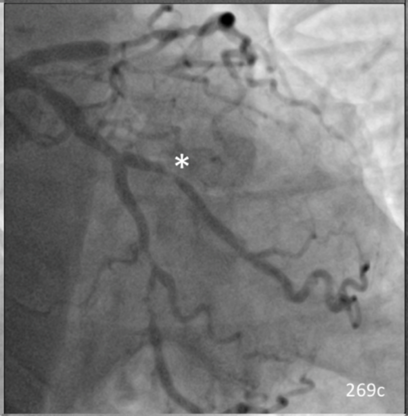
269c

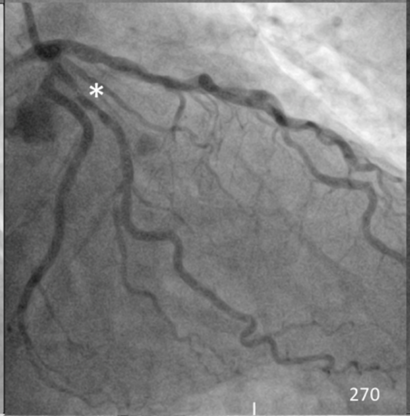
270

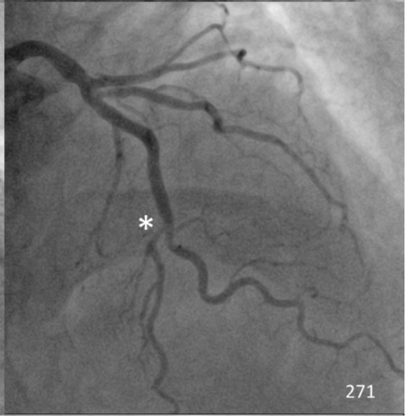
271

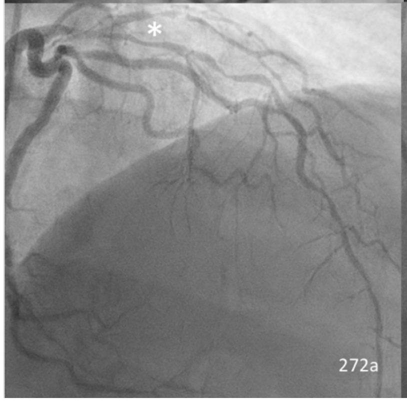
272a

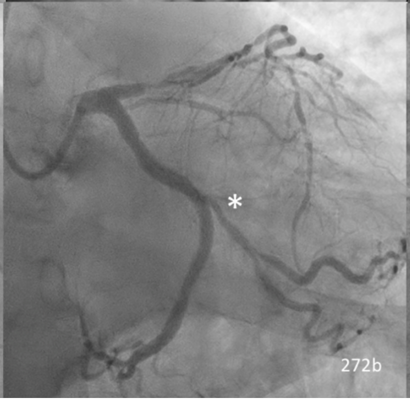
272b

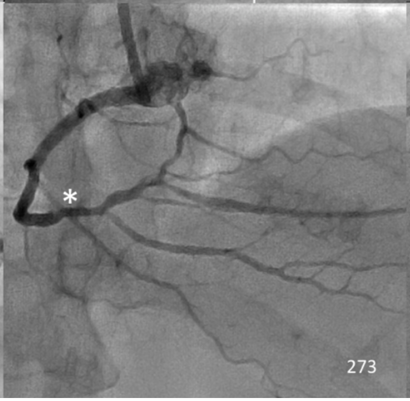
273

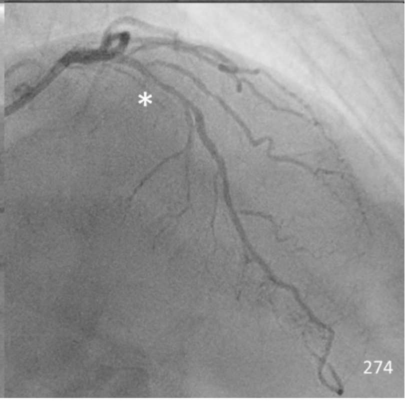
274

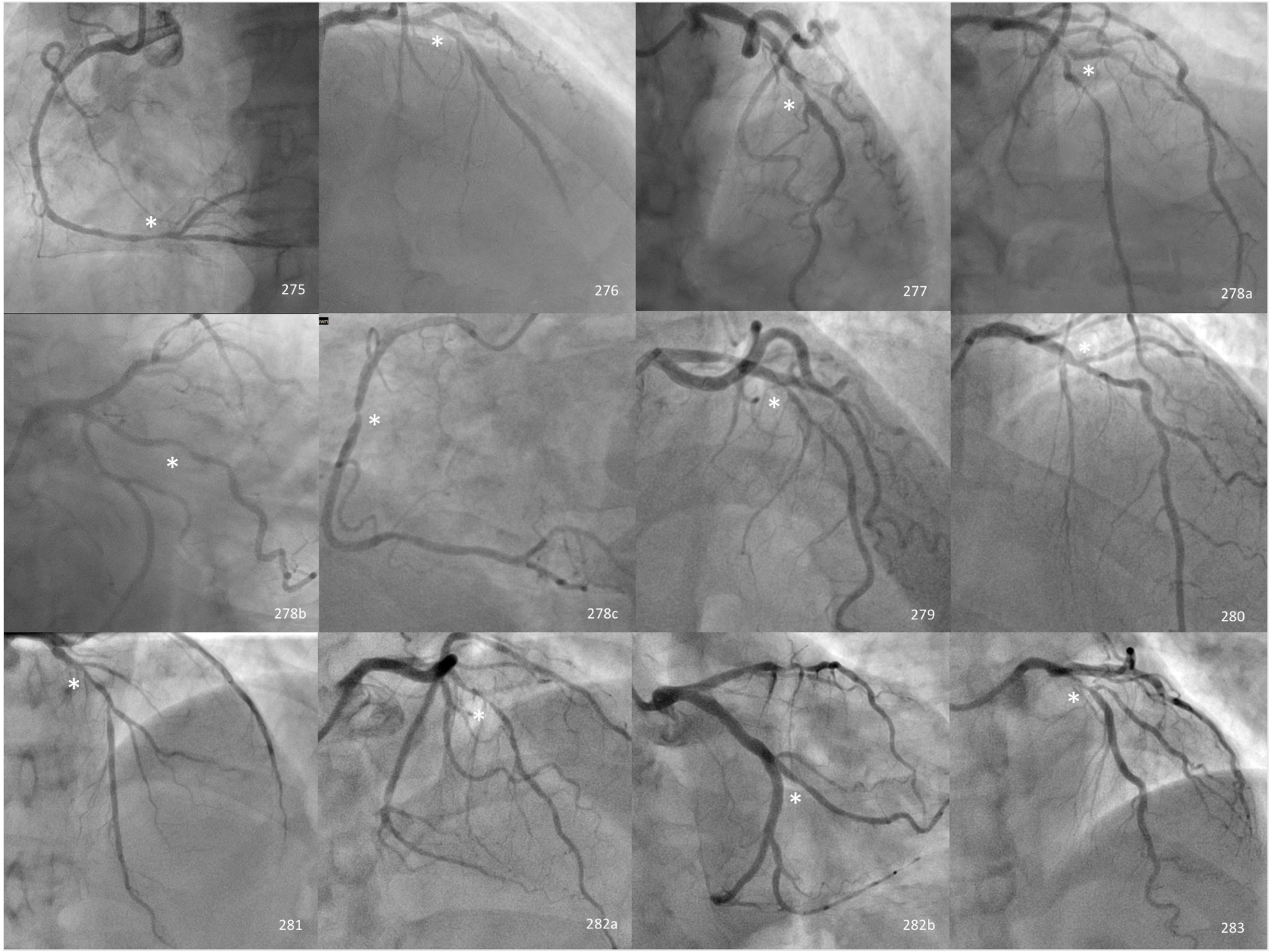
275
276
277
278a
278b
278c
279
280
281
282a
282b
283

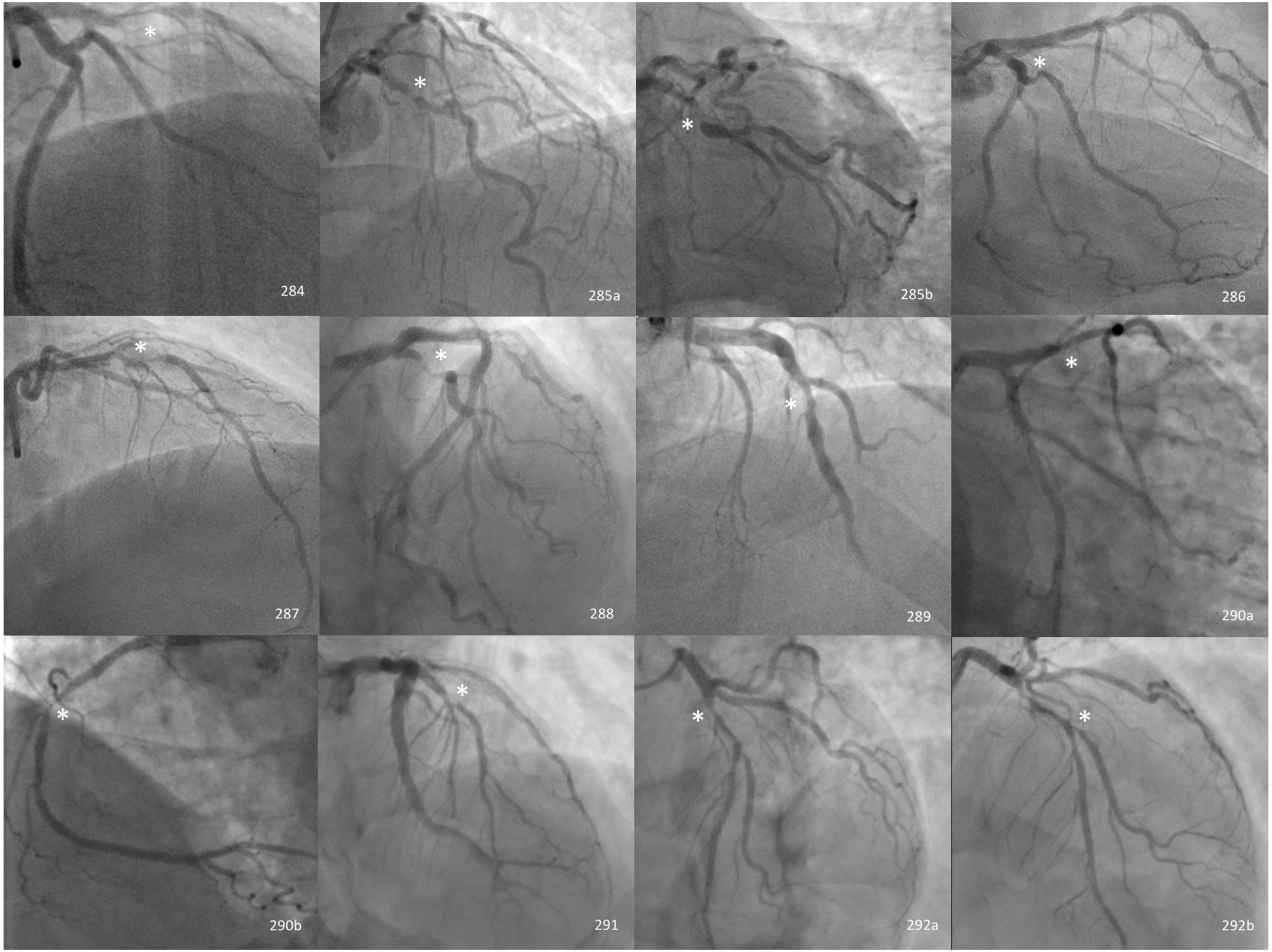
284
285a
285b
286
287
288
289
290a
290b
291
292a
292b

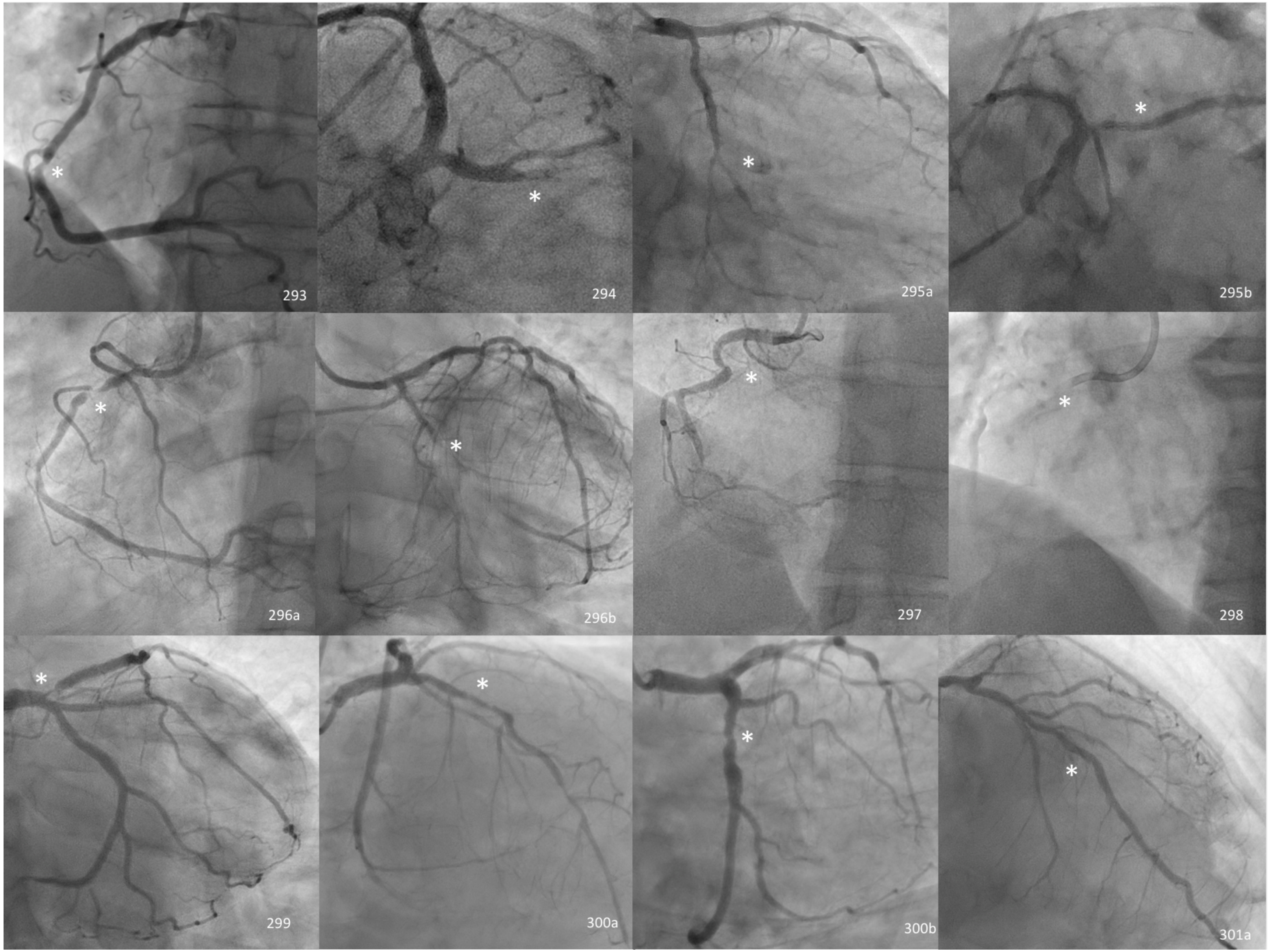
293
294
295a
295b
296a
296b
297
298
299
300a
300b
301a

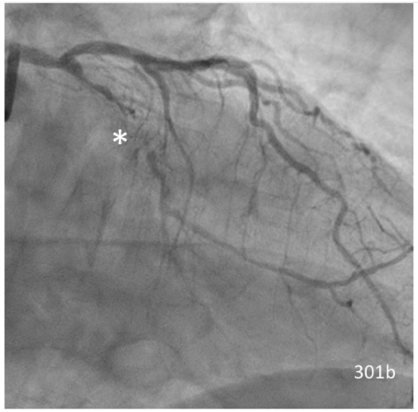
*
301b

Primary End Point: Angina Symptom Score

Supplementary Figure S4: Daily transition odds ratios for angina symptom score

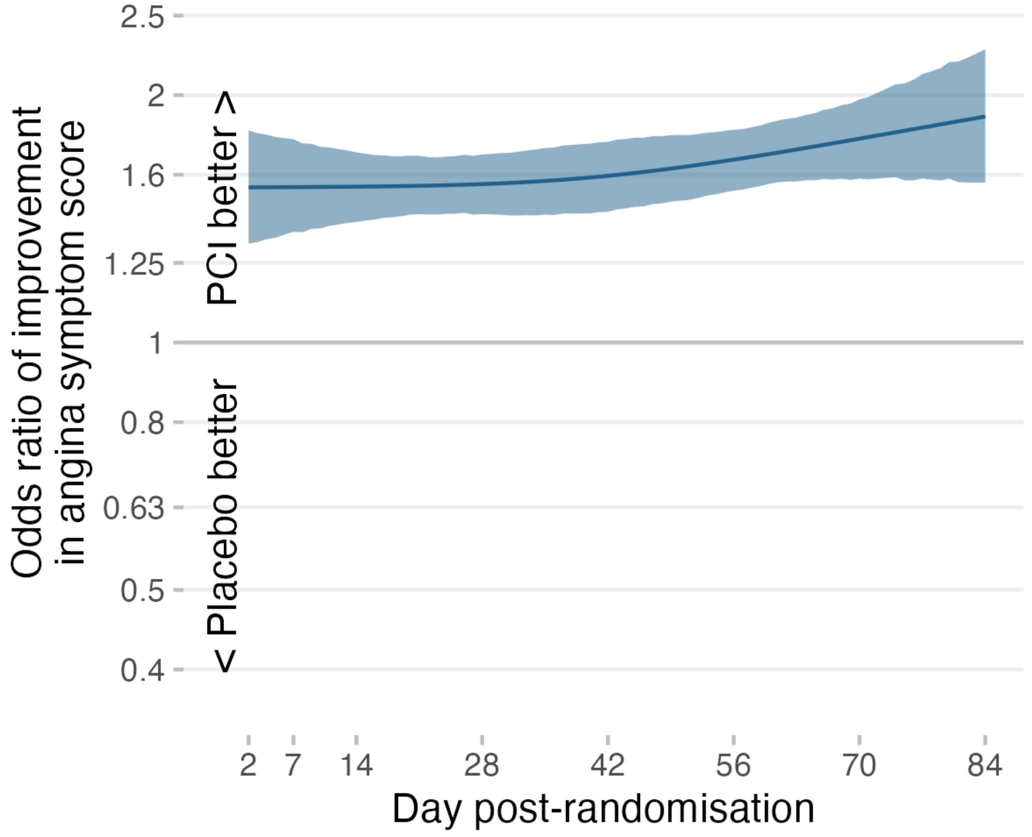

Supplementary Figure S5: Regression model and coefficients for angina symptom score

For the purposes of statistical coding the angina symptoms score is referred to as "orbita_score"

Bayesian Constrained Partial Proportional Odds Ordinal Logistic Model Dirichlet Priors With Concentration Parameter 0.055 for Intercepts

```
blrm(formula = orbita_score_num ~ rcs(orbita_score_num_lag1,
    4) + rcs(day_num_m2, 3) * Treatment + rcs(orbita_score_num_pre_mean,
    4), ppo = ~day_num_m2 + Treatment, cppo = function(y) y,
    keepsep = "^Treatment=PCI$", data = main_analysis_d, priorsd = c(rep(100,
        3), sap_treatment_sd_prior, rep(100, 4)), iter = 4000,
    chains = 8, refresh = 100, progress = "./output/orbita_score_res24.refresh.txt",
    loo = FALSE, ppairs = NULL, file = "./output/orbita_score_res24.blrm.rds")
```

	Mixed Calibration/ Discrimination Indexes	Discrimination Indexes	Rank Discrim. Indexes
Obs 22759	B 0.113 [0.113, 0.113]	g 7.489 [7.35, 7.624]	C 0.916 [0.916, 0.917]
Draws 16000		g_p 0.409 [0.406, 0.412]	D_{xy} 0.832 [0.831, 0.833]
Chains 8		EV 0.567 [0.559, 0.575]	
p 10		v 86.789 [82.069, 90.755]	
		vp 0.141 [0.139, 0.143]	

	Mean β	Median β	S.E.	Lower	Upper	Pr(β>0)	Symmetry
orbita_score_num_lag1	1.5384	1.5384	0.0210	1.4992	1.5811	1.0000	1.00
orbita_score_num_lag1'	-2.7020	-2.7028	0.1779	-3.0599	-2.3656	0.0000	1.01
day_num_m2	-0.0022	-0.0022	0.0019	-0.0059	0.0016	0.1308	0.99
day_num_m2'	0.0010	0.0010	0.0023	-0.0035	0.0057	0.6629	1.00
Treatment=PCI	-0.3693	-0.3688	0.0805	-0.5322	-0.2182	0.0000	0.99
orbita_score_num_pre_mean	1.1674	1.1673	0.0630	1.0445	1.2899	1.0000	1.02
orbita_score_num_pre_mean'	-101.1559	-101.1041	5.8381	-112.4133	-89.5851	0.0000	0.99
orbita_score_num_pre_mean''	132.4975	132.4231	7.6675	117.3645	147.3590	1.0000	1.01
day_num_m2 × Treatment=PCI	-0.0002	-0.0002	0.0035	-0.0073	0.0066	0.4831	1.02
day_num_m2' × Treatment=PCI	-0.0029	-0.0029	0.0045	-0.0119	0.0055	0.2511	0.99
day_num_m2 x f(y)	0.0000	0.0000	0.0006	-0.0012	0.0012	0.4968	0.99
Treatment=PCI x f(y)	0.1991	0.1991	0.0288	0.1435	0.2564	1.0000	1.01

The following parameters remained separate (where not orthogonalized) during model fitting so that prior distributions could be focused explicitly on them: Treatment=PCI

Supplementary Figure S6: Assessment of model fit by predicted state occupancy for angina symptom score.

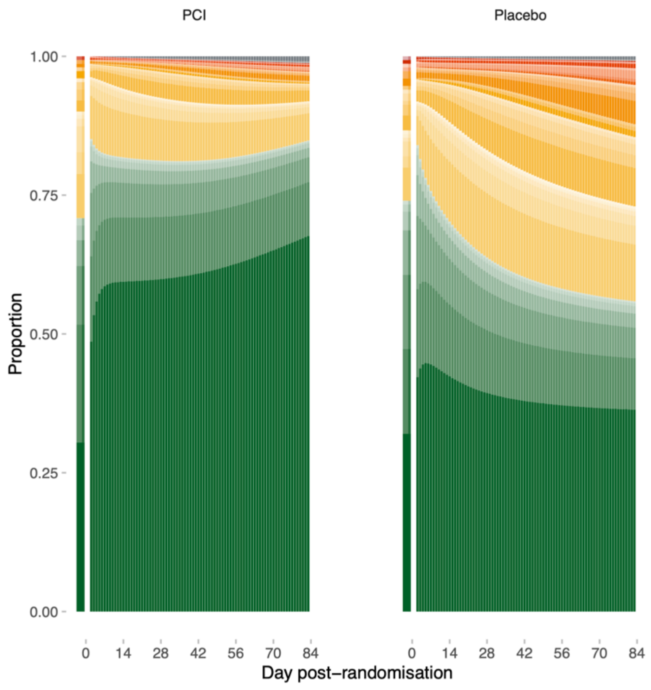

Supplementary Figure S7: Coefficient density plots: angina symptom score

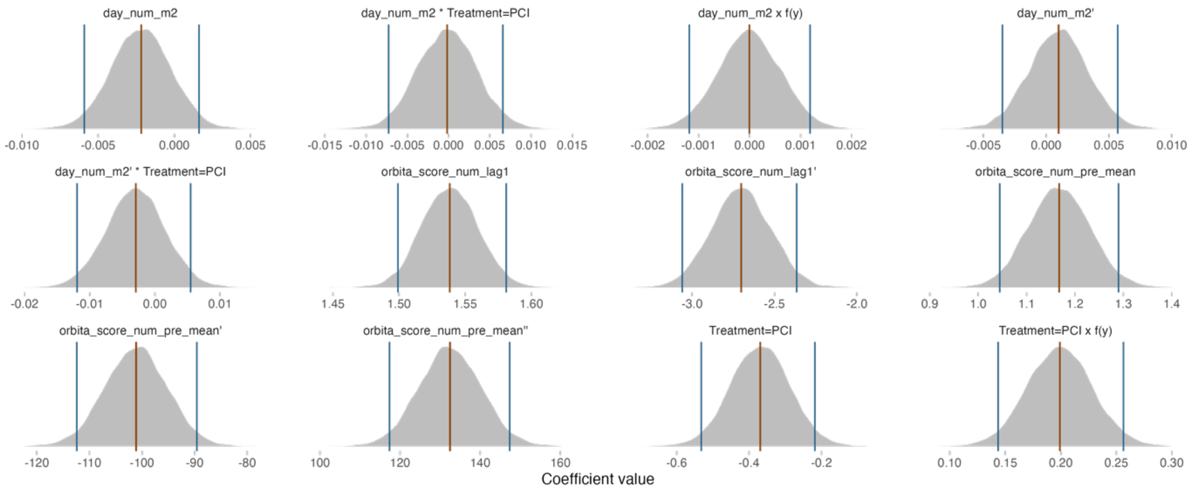

The red line is the posterior median value and the blue lines are the posterior 5th and 95th quantiles.

Supplementary Figure S8: Chain plot of Markov chain Monte Carlo draws for angina symptom score

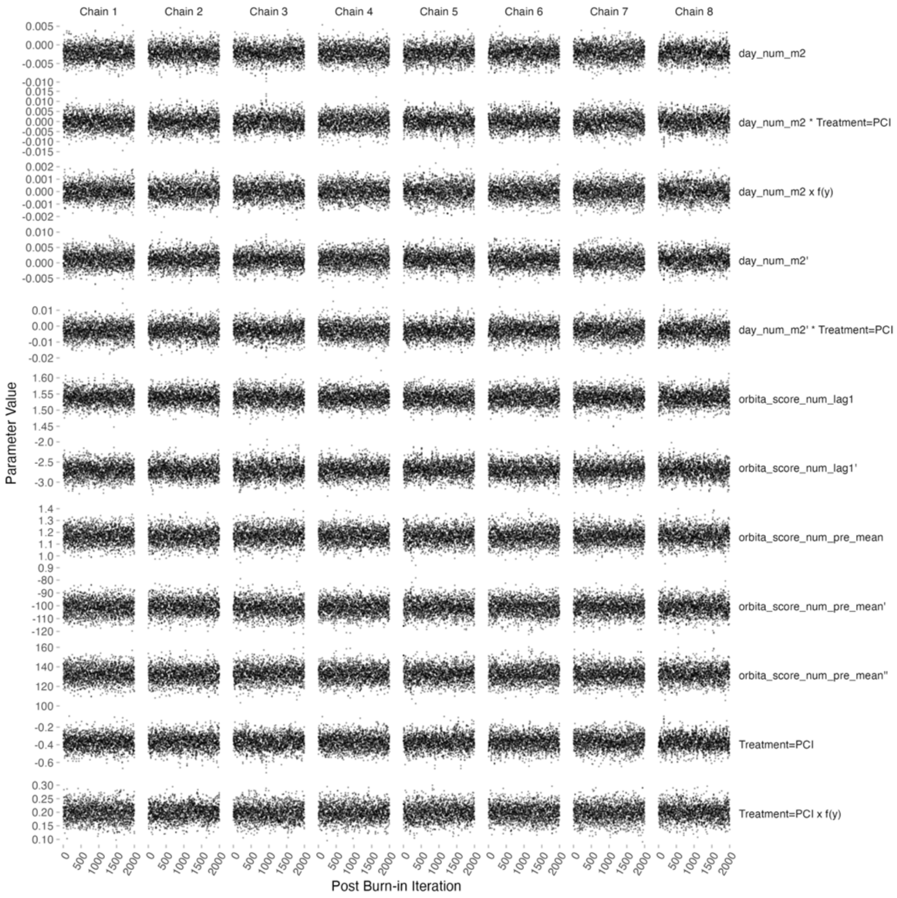

Components of Primary End Point: Daily angina episodes

Supplementary Figure S9: Daily transition odds ratios for daily angina episodes

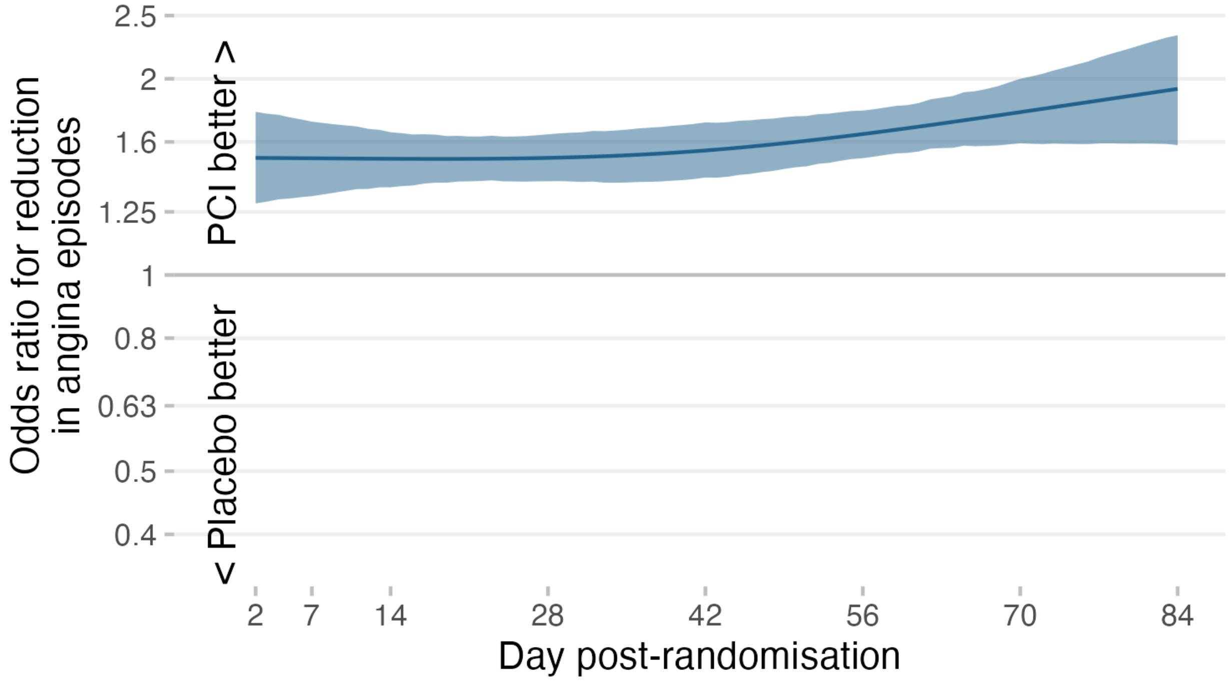

Supplementary Figure S10: Regression model and coefficients for daily angina episodes

Bayesian Constrained Partial Proportional Odds Ordinal Logistic Model Dirichlet Priors With Concentration Parameter 0.308 for Intercepts

```
blrm(formula = symptom_frequency_num ~ rcs(symptom_frequency_num_lag1,
    c(1, 3, 5)) + rcs(day_num_m2, 3) * Treatment + rcs(symptom_frequency_num_pre_mean,
    4), ppo = ~day_num_m2, cppo = function(y) y, keepsep = "^Treatment=PCI$",
    data = main_analysis_d, priorsd = c(rep(100, 4), sap_treatment_sd_prior,
        rep(100, 5)), iter = 5000, chains = 8, refresh = 100,
    progress = "./output/symptom_frequency_res40.refresh.txt",
    loo = FALSE, ppairs = NULL, file = "./output/symptom_frequency_res40.blrm.rds")
```

Frequencies of Responses

0	1	2	3	4	5	6
15092	3815	2274	933	439	135	71

	Mixed Calibration/ Discrimination Indexes	Discrimination Indexes	Rank Discrim. Indexes
Obs 22759	B 0.147 [0.147, 0.147]	g 1.777 [1.737, 1.826]	C 0.827 [0.826, 0.828]
Draws 20000		g_p 0.3 [0.297, 0.305]	D_{xy} 0.654 [0.652, 0.656]
Chains 8		EV 0.362 [0.353, 0.371]	
p 10		v 2.809 [2.69, 2.933]	
		vp 0.081 [0.079, 0.083]	

	Mode β	Mean β	Median β	S.E.	Lower	Upper	Pr(β>0)	Symmetry
y≥1	-2.2566	-2.2678	-2.2682	0.0866	-2.4430	-2.1007	0.0000	0.98
y≥2	-3.7381	-3.7496	-3.7496	0.0890	-3.9183	-3.5680	0.0000	0.98
y≥3	-5.3702	-5.3821	-5.3820	0.0981	-5.5798	-5.1957	0.0000	0.98
y≥4	-6.7293	-6.7417	-6.7405	0.1127	-6.9600	-6.5203	0.0000	0.97
y≥5	-8.1609	-8.1746	-8.1734	0.1382	-8.4466	-7.9080	0.0000	0.98
y≥6	-9.3371	-9.3546	-9.3527	0.1792	-9.7108	-9.0151	0.0000	0.96
symptom_frequency_num_lag1	1.3849	1.3849	1.3848	0.0216	1.3437	1.4285	1.0000	1.02
symptom_frequency_num_lag1'	-0.5097	-0.5086	-0.5087	0.0497	-0.6040	-0.4103	0.0000	1.01
day_num_m2	-0.0042	-0.0038	-0.0038	0.0025	-0.0087	0.0011	0.0622	1.00
day_num_m2'	0.0023	0.0020	0.0020	0.0031	-0.0041	0.0079	0.7431	1.00
Treatment=PCI	-0.4319	-0.4135	-0.4131	0.0830	-0.5762	-0.2529	0.0000	0.99
symptom_frequency_num_pre_mean	1.5375	1.5424	1.5423	0.1493	1.2507	1.8342	1.0000	1.00
symptom_frequency_num_pre_mean'	-5.2880	-5.3156	-5.3203	1.1692	-7.6285	-3.0296	0.0000	1.01
symptom_frequency_num_pre_mean''	8.0892	8.1359	8.1386	2.1274	3.7991	12.1698	1.0000	0.99
day_num_m2 × Treatment=PCI	0.0011	0.0003	0.0003	0.0037	-0.0073	0.0072	0.5312	1.02
day_num_m2' × Treatment=PCI	-0.0050	-0.0042	-0.0042	0.0047	-0.0134	0.0048	0.1842	0.99
day_num_m2 x f(y)	0.0005	0.0005	0.0005	0.0006	-0.0006	0.0016	0.8021	0.99

The following parameters remained separate (where not orthogonalized) during model fitting so that prior distributions could be focused explicitly on them: Treatment=PCI

Supplementary Figure S11: Assessment of model fit by predicted state occupancy for daily angina episodes

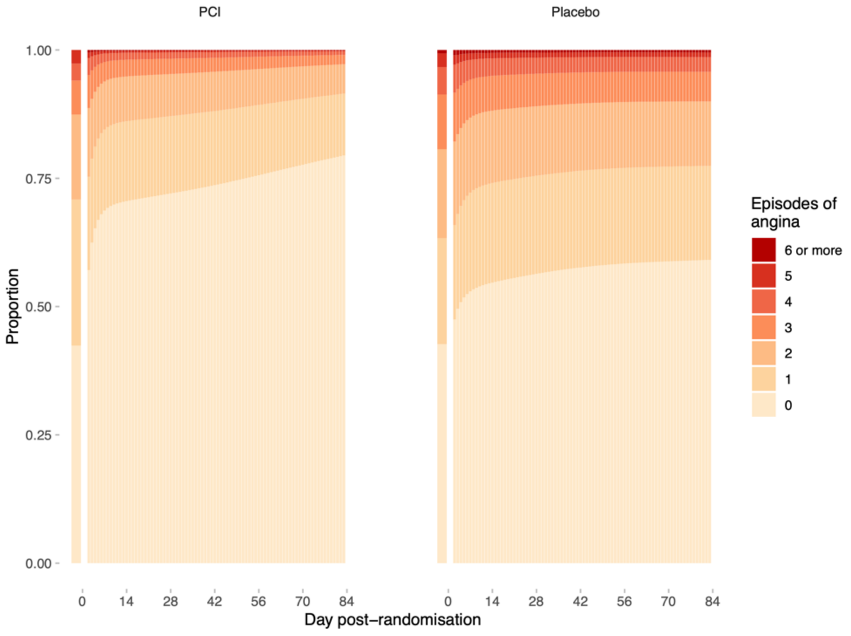

Supplementary Figure S12: Coefficient density plots: daily angina episodes

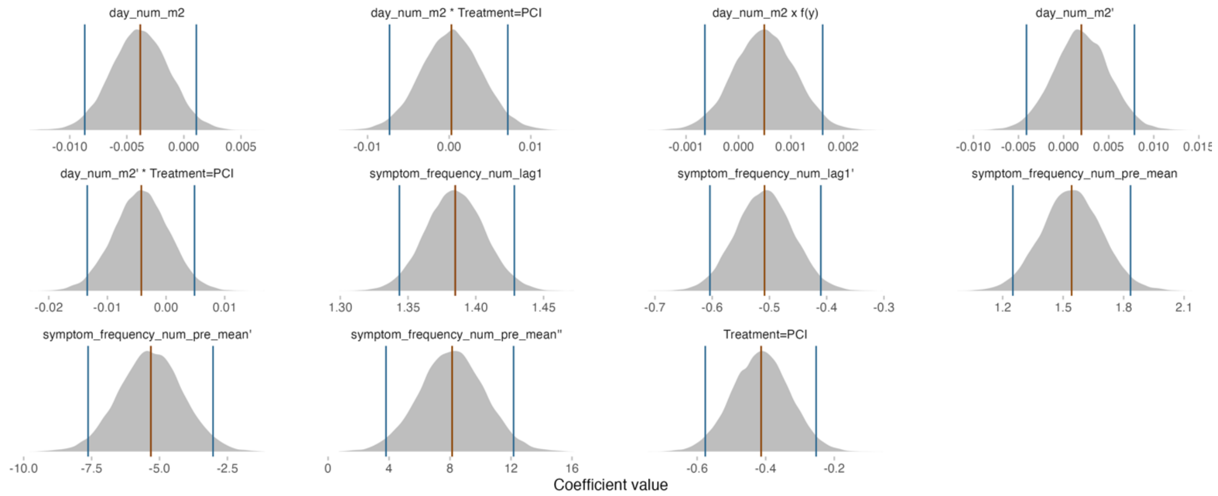

The red line is the posterior median value and the blue lines are the posterior 5th and 95th quantiles.

Supplementary Figure S13: Chain plot of Markov chain Monte Carlo draws for daily angina episodes

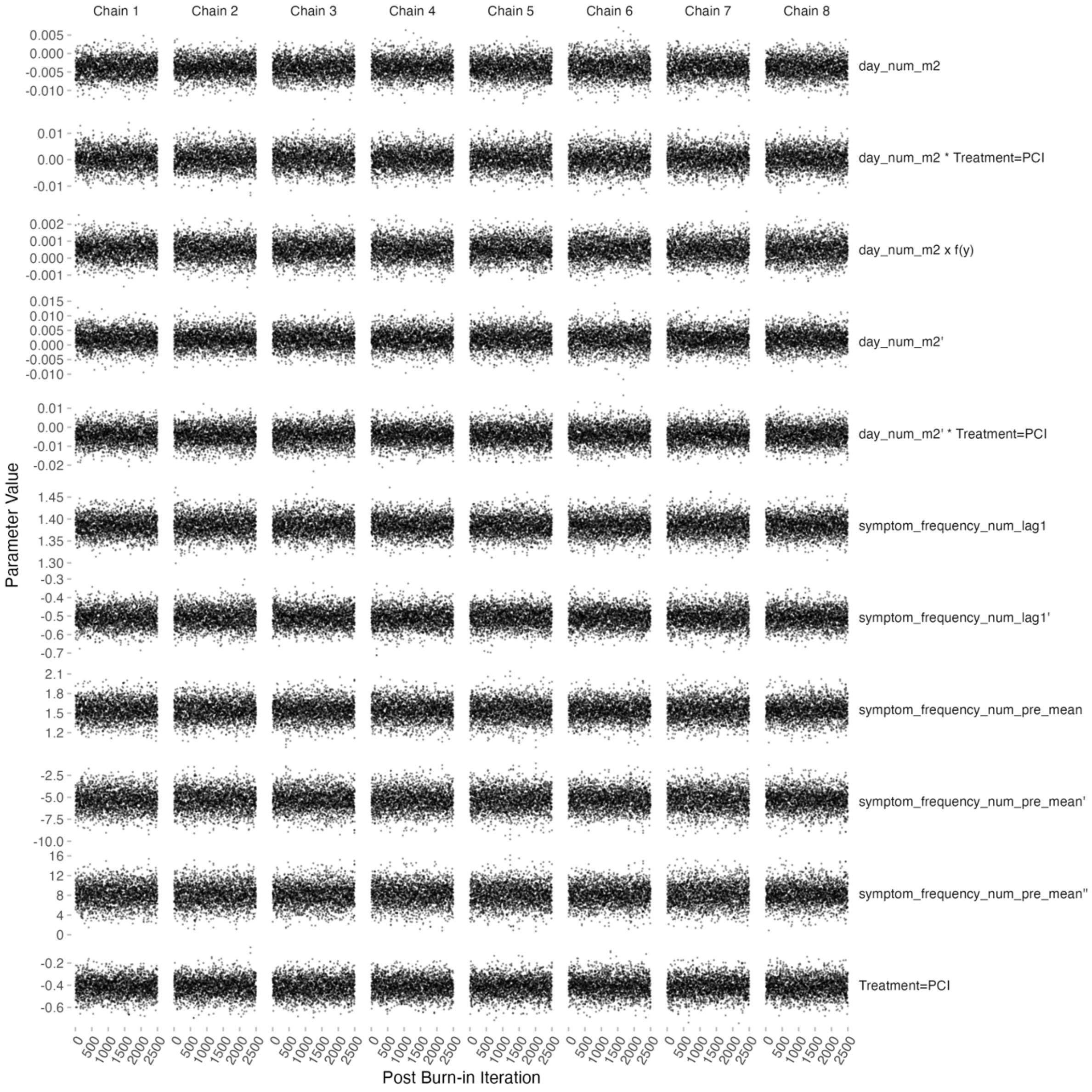

Components of Primary End Point: Daily antianginal medication units

Supplementary Figure S14: Daily transition odds ratios for daily antianginal medication units

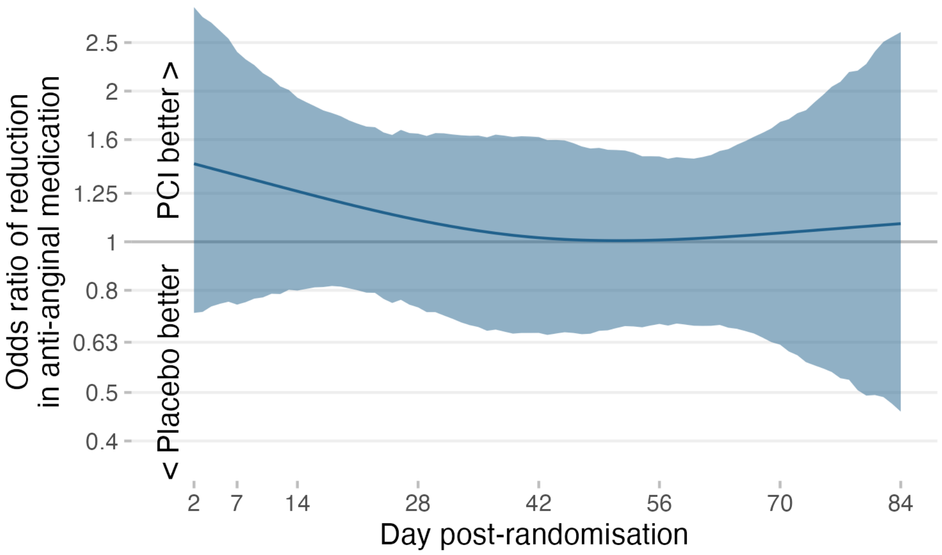

Supplementary Figure S15: Regression model and coefficients for daily antianginal medication units

Bayesian Constrained Partial Proportional Odds Ordinal Logistic Model Dirichlet Priors With Concentration Parameter 0.278 for Intercepts

```
blrm(formula = aa_unit_dose_clip_num ~ rcs(aa_unit_dose_clip_num_lag1,
    c(1, 3, 5)) + rcs(day_num_m2, 3) * Treatment + rcs(aa_unit_dose_clip_num_pre_mean,
    4), ppo = ~day_num_m2, cppo = function(y) y, keepsep = "^Treatment=PCI$",
    data = main_analysis_d, priorsd = c(rep(100, 4), sap_treatment_sd_prior,
        rep(100, 5)), iter = 5000, chains = 8, refresh = 100,
    progress = "./output/orbita_aa_res40.refresh.txt", loo = FALSE,
    ppairs = NULL, file = "./output/orbita_aa_res40.blrm.rds")
```

Frequencies of Responses

```
    0     1     2     3     4     5     6     7
17830  2872  1075   109   333   189   214   137
```

	Mixed Calibration/ Discrimination Indexes	Discrimination Indexes	Rank Discrim. Indexes
Obs 22759	B 0.002 [0.002, 0.002]	g 7.106 [6.872, 7.365]	C 0.996 [0.995, 0.997]
Draws 20000		g_p 0.335 [0.334, 0.335]	D_{xy} 0.992 [0.991, 0.993]
Chains 8		EV 0.973 [0.969, 0.978]	
p 9		v 98.448 [91.696, 104.646]	
		vp 0.165 [0.164, 0.166]	

	Mode β	Mean β	Median β	S.E.	Lower	Upper	Pr(β>0)	Symmetry
y≥1	-5.2133	-5.2282	-5.2236	0.2828	-5.7981	-4.6870	0.0000	0.95
y≥2	-14.6095	-14.5563	-14.5545	0.3785	-15.3041	-13.8247	0.0000	0.94
y≥3	-24.5018	-24.3780	-24.3710	0.5360	-25.4219	-23.3309	0.0000	0.95
y≥4	-30.9050	-30.7222	-30.7078	0.6724	-32.0382	-29.4237	0.0000	0.95
y≥5	-40.1126	-39.8405	-39.8240	0.7630	-41.3305	-38.3448	0.0000	0.96
y≥6	-46.9136	-46.5781	-46.5543	0.9769	-48.5773	-44.7422	0.0000	0.95
y≥7	-54.3764	-53.9855	-53.9453	1.2637	-56.4968	-51.5626	0.0000	0.93
aa_unit_dose_clip_num_lag1	9.9658	9.8916	9.8883	0.1890	9.5208	10.2609	1.0000	1.06
aa_unit_dose_clip_num_lag1'	-1.9137	-1.8991	-1.9018	0.2675	-2.4223	-1.3762	0.0000	1.05
day_num_m2	-0.0135	-0.0123	-0.0124	0.0117	-0.0343	0.0112	0.1478	1.00
day_num_m2'	0.0061	0.0053	0.0053	0.0143	-0.0224	0.0333	0.6440	0.99
Treatment=PCI	-0.3764	-0.3588	-0.3589	0.3602	-1.0791	0.3271	0.1603	1.01
aa_unit_dose_clip_num_pre_mean	0.3875	0.3926	0.3937	0.2051	-0.0105	0.7988	0.9705	0.98
aa_unit_dose_clip_num_pre_mean'	-0.3307	-0.3362	-0.3384	0.1969	-0.7158	0.0577	0.0443	1.00
day_num_m2 × Treatment=PCI	0.0109	0.0106	0.0107	0.0159	-0.0202	0.0414	0.7458	0.99
day_num_m2' × Treatment=PCI	-0.0097	-0.0094	-0.0094	0.0206	-0.0489	0.0313	0.3258	1.00
day_num_m2 x f(y)	-0.0006	-0.0006	-0.0006	0.0019	-0.0045	0.0031	0.3748	0.99

The following parameters remained separate (where not orthogonalized) during model fitting so that prior distributions could be focused explicitly on them: Treatment=PCI

Supplementary Figure S16: Assessment of model fit by predicted state occupancy for daily antianginal medication units

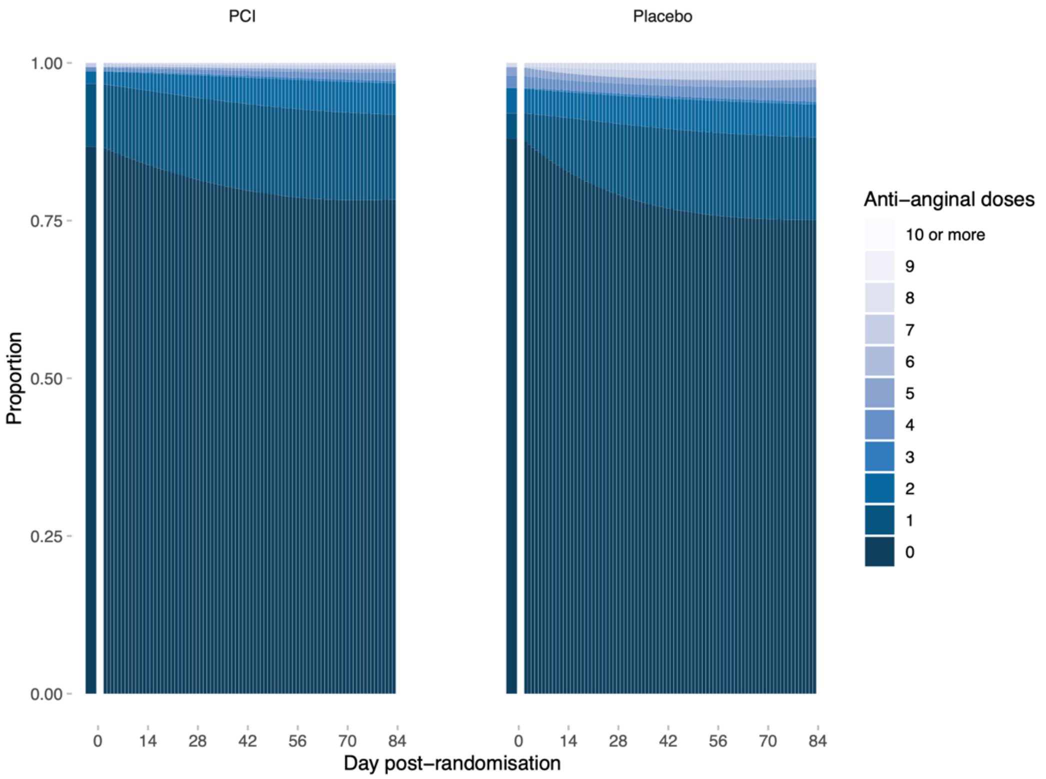

Supplementary Figure S17: Coefficient density plots: daily antianginal medication units

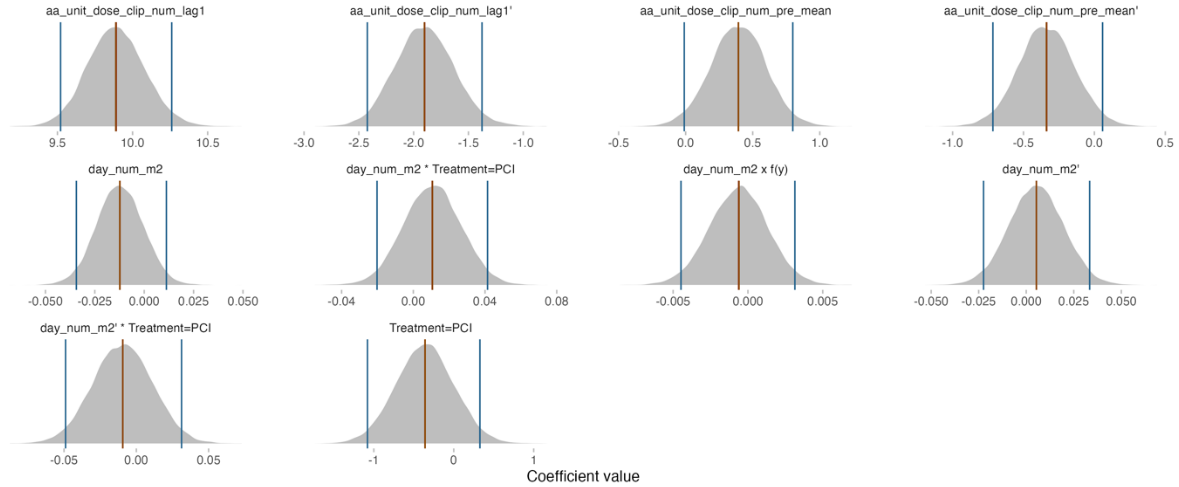

The red line is the posterior median value and the blue lines are the posterior 5th and 95th quantiles.

Supplementary Figure S18: Chain plot of Markov chain Monte Carlo draws for daily antianginal medication units

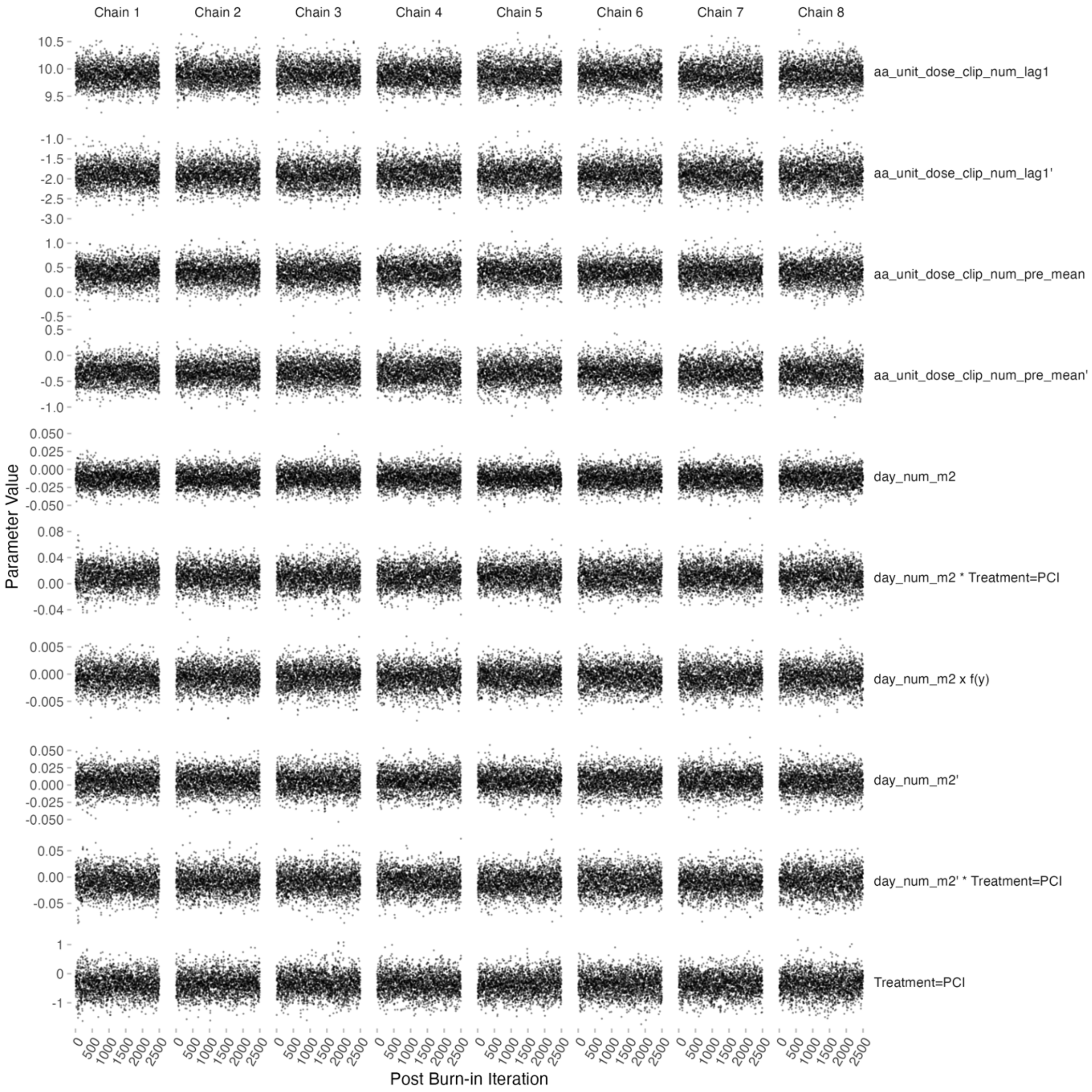

Secondary End Points - Treadmill exercise time

Supplementary Figure S19: Regression model and coefficients for treadmill exercise time

Bayesian Proportional Odds Ordinal Logistic Model Dirichlet Priors With Concentration Parameter 0.014 for Intercepts

```
blrm(formula = fu_ett_seconds ~ rcs(baseline_ett_seconds, 3) +
    Treatment, keepsep = "^Treatment=PCI$", data = ett_res1_d,
    priorsd = c(rep(100, 2), sap_treatment_sd_prior), iter = 20000,
    chains = 4, refresh = 100, ppairs = NULL, file = "./output/ett_res1.blrm.rds")
```

	Mixed Calibration/ Discrimination Indexes	Discrimination Indexes	Rank Discrim. Indexes
Obs 235	LOO log L -1601.42±19.04	g 2.479 [2.055, 2.814]	C 0.777 [0.775, 0.779]
Draws 40000	LOO IC 3202.84±38.08	g_p 0.376 [0.35, 0.399]	D_{xy} 0.554 [0.549, 0.558]
Chains 4	Effective p 460.13±11.04	EV 0.432 [0.371, 0.487]	
p 3	B 0.152 [0.149, 0.154]	v 4.863 [3.459, 6.414]	
		vp 0.108 [0.093, 0.122]	

	Mode β	Mean β	Median β	S.E.	Lower	Upper	Pr(β>0)	Symmetry
baseline_ett_seconds	0.0095	0.0096	0.0096	0.0014	0.0068	0.0123	1.0000	1.04
baseline_ett_seconds'	-0.0007	-0.0008	-0.0008	0.0013	-0.0033	0.0018	0.2762	1.00
Treatment=PCI	0.5628	0.5687	0.5676	0.2202	0.1406	1.0009	0.9954	0.99

The following parameters remained separate (where not orthogonalized) during model fitting so that prior distributions could be focused explicitly on them: Treatment=PCI

Supplementary Figure S20: Coefficient density plots: treadmill exercise time

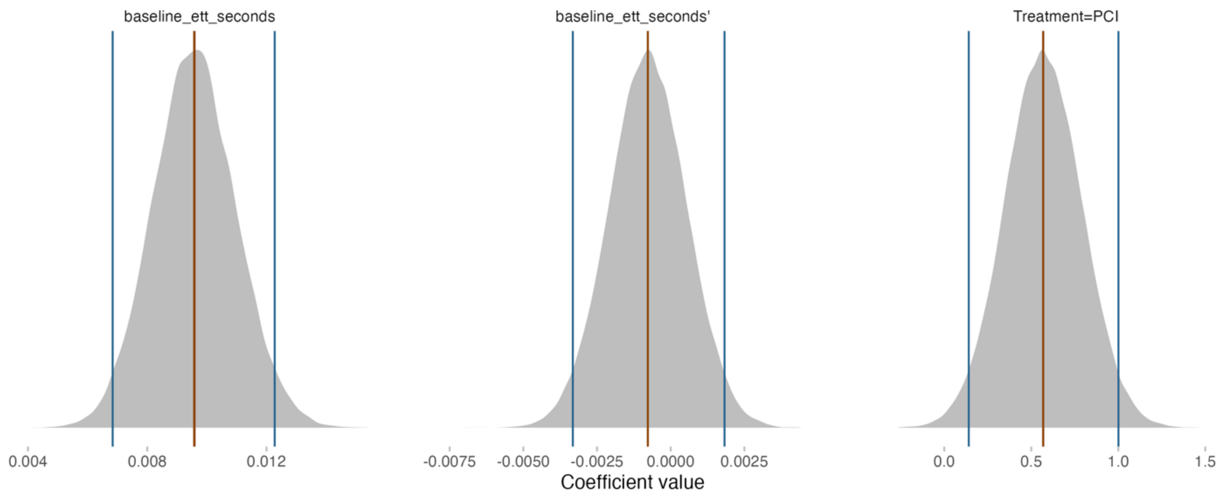

The red line is the posterior median value and the blue lines are the posterior 5th and 95th quantiles.

Supplementary Figure S21: Chain plot of Markov chain Monte Carlo draws for treadmill exercise time

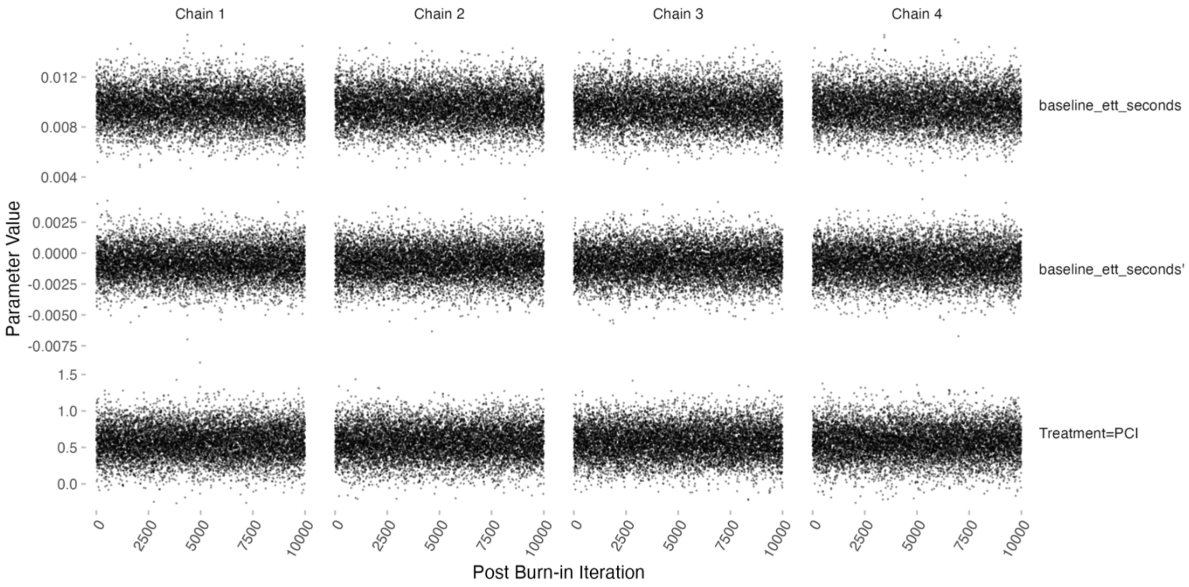

Secondary End Points - Canadian Cardiovascular Society (CCS) Class

Supplementary Figure S22: Regression model and coefficients for CCS Class

Bayesian Proportional Odds Ordinal Logistic Model Dirichlet Priors With Concentration Parameter 0.392 for Intercepts

```
blrm(formula = num_ccs_fu ~ num_ccs_rand + Treatment, keepsep = "^Treatment=PCI$",
    data = ccs_res1_d, priorsd = c(100, sap_treatment_sd_prior),
    iter = 20000, chains = 4, refresh = 100, file = "./output/ccs_res1.blrm.rds")
```

Frequencies of Responses

```
 0  1  2  3  4
82 64 89 51  7
```

	Mixed Calibration/ Discrimination Indexes	Discrimination Indexes	Rank Discrim. Indexes
Obs 293	LOO log L -402.61±9.16	g 0.84 [0.564, 1.062]	C 0.719 [0.721, 0.721]
Draws 40000	LOO IC 805.21±18.33	g_p 0.191 [0.147, 0.244]	D_{xy} 0.438 [0.441, 0.441]
Chains 4	Effective p 5.98±0.39	EV 0.121 [0.06, 0.177]	
p 2	B 0.214 [0.212, 0.218]	v 0.592 [0.262, 0.911]	
		vp 0.03 [0.015, 0.044]	

	Mode β	Mean β	Median β	S.E.	Lower	Upper	Pr(β>0)	Symmetry
y≥1	-0.0641	-0.0652	-0.0641	0.4724	-1.0122	0.8385	0.4454	0.99
y≥2	-1.1286	-1.1348	-1.1325	0.4730	-2.0593	-0.2029	0.0080	0.98
y≥3	-2.7234	-2.7322	-2.7278	0.4986	-3.7097	-1.7553	0.0000	0.97
y≥4	-5.1467	-5.1770	-5.1633	0.6240	-6.4024	-3.9596	0.0000	0.95
num_ccs_rand	0.7529	0.7574	0.7569	0.1918	0.3760	1.1273	1.0000	1.02
Treatment=PCI	-1.2373	-1.2400	-1.2406	0.2147	-1.6529	-0.8090	0.0000	0.99

The following parameters remained separate (where not orthogonalized) during model fitting so that prior distributions could be focused explicitly on them: Treatment=PCI

Supplementary Figure S23: Coefficient density plots: CCS Class

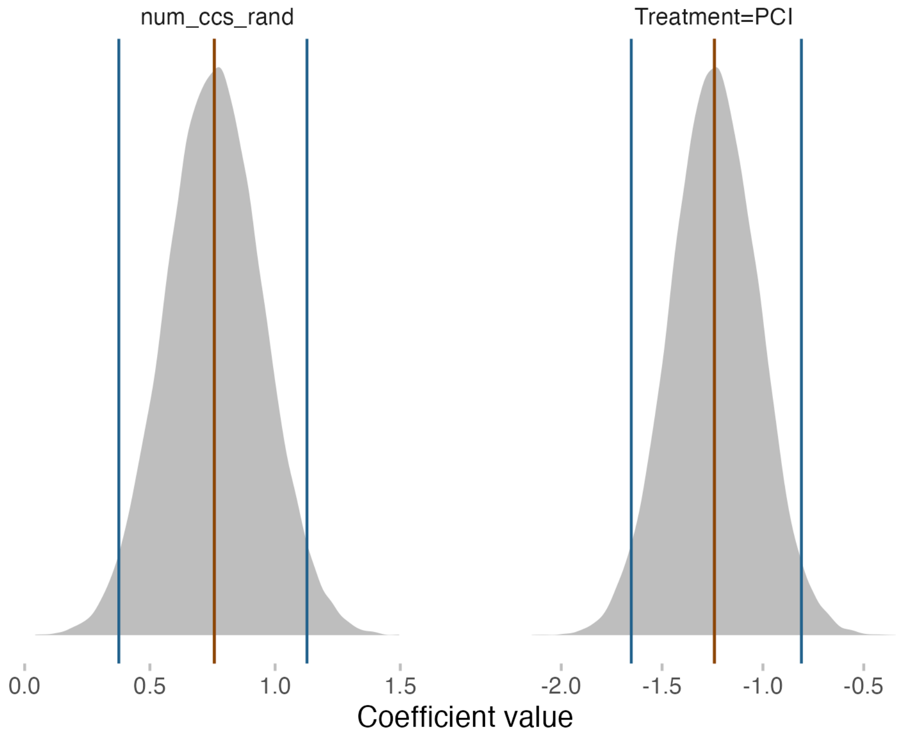

The red line is the posterior median value and the blue lines are the posterior 5th and 95th quantiles.

Supplementary Figure S24: Chain plot of Markov chain Monte Carlo draws for CCS Class

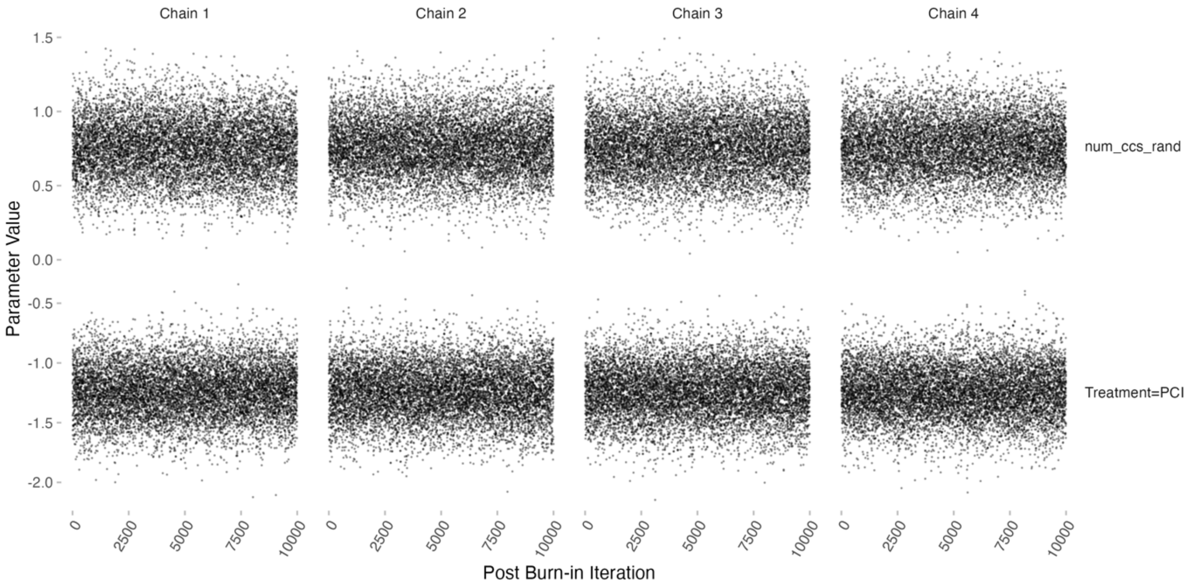

Secondary End Points - Seattle Angina Questionnaire (SAQ) angina frequency

Supplementary Figure S25: Regression model and coefficients for SAQ angina frequency

Bayesian Proportional Odds Ordinal Logistic Model Dirichlet Priors With Concentration Parameter 0.215 for Intercepts

```
blrm(formula = outcome_saq_angina_freq_post ~ rcs(outcome_saq_angina_freq_pre,
    3) + Treatment, keepsep = "^Treatment=PCI$", data = saq_freq_res1_d,
    priorsd = c(rep(100, 2), sap_treatment_sd_prior), iter = 20000,
    chains = 4, refresh = 100, file = "./output/saq_freq_res1.blrm.rds")
```

	Mixed Calibration/ Discrimination Indexes	Discrimination Indexes	Rank Discrim. Indexes
Obs 291	LOO log L -564.98±13.39	g 1.133 [0.862, 1.38]	C 0.693 [0.682, 0.702]
Draws 40000	LOO IC 1129.97±26.79	g_p 0.239 [0.194, 0.277]	D_{xy} 0.385 [0.365, 0.404]
Chains 4	Effective p 15.45±2.47	EV 0.177 [0.119, 0.237]	
p 3	B 0.208 [0.204, 0.214]	v 1.02 [0.572, 1.495]	
		vp 0.044 [0.029, 0.058]	

	Mode β	Mean β	Median β	S.E.	Lower	Upper	Pr(β>0)	Symmetry
outcome_saq_angina_freq_pre	0.0214	0.0213	0.0213	0.0130	-0.0044	0.0465	0.9498	1.01
outcome_saq_angina_freq_pre'	0.0160	0.0163	0.0162	0.0126	-0.0084	0.0408	0.9023	1.03
Treatment=PCI	1.1581	1.1588	1.1560	0.2130	0.7364	1.5744	1.0000	1.02

The following parameters remained separate (where not orthogonalized) during model fitting so that prior distributions could be focused explicitly on them: Treatment=PCI

Supplementary Figure S26: Coefficient density plots: SAQ angina frequency

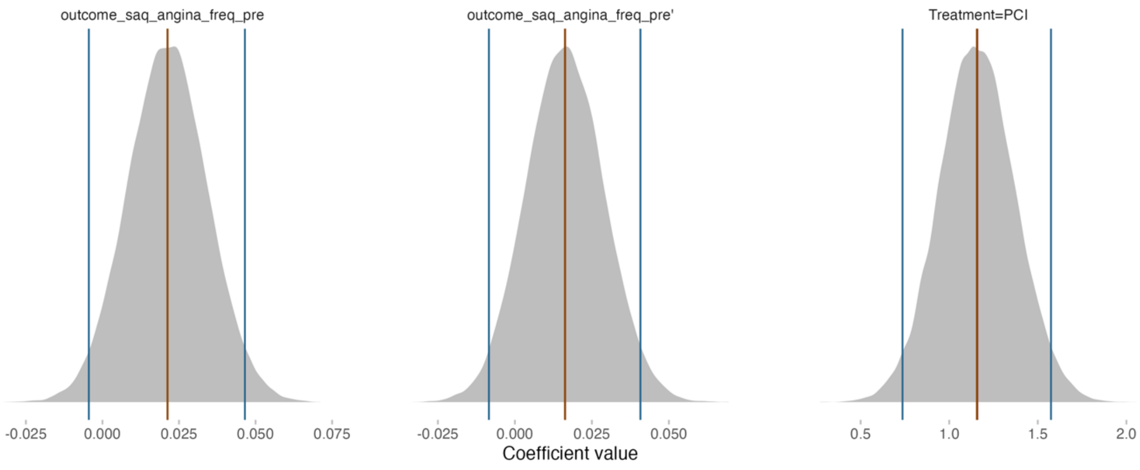

The red line is the posterior median value and the blue lines are the posterior 5th and 95th quantiles.

Supplementary Figure S27: Chain plot of Markov chain Monte Carlo draws for SAQ angina frequency

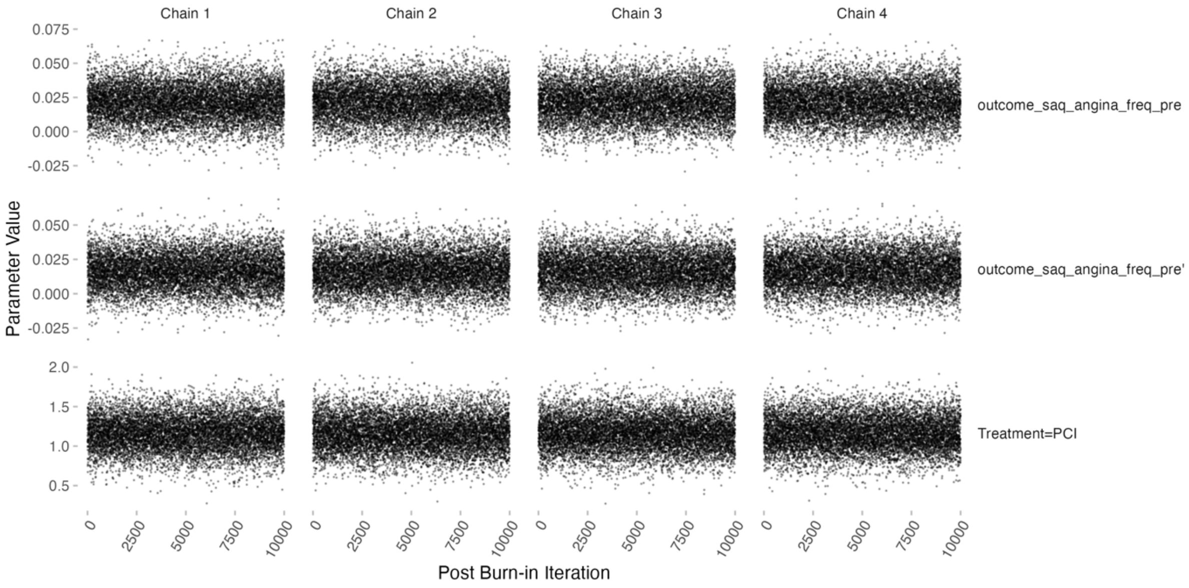

Secondary End Points - Seattle Angina Questionnaire (SAQ) physical limitation

Supplementary Figure S28: Regression model and coefficients for SAQ physical limitation

Bayesian Proportional Odds Ordinal Logistic Model Dirichlet Priors With Concentration Parameter 0.052 for Intercepts

```
blrm(formula = outcome_saq_pl_post ~ rcs(outcome_saq_pl_pre,
    3) + Treatment, keepsep = "^Treatment=PCI$", data = saq_pl_res1_d,
    priorsd = c(rep(100, 2), sap_treatment_sd_prior), iter = 20000,
    chains = 4, refresh = 100, file = "./output/saq_pl_res1.blrm.rds")
```

	**Mixed Calibration/ Discrimination Indexes**	**Discrimination Indexes**	**Rank Discrim. Indexes**
Obs 283	LOO log L -962.7±26.6	g 1.785 [1.514, 2.07]	C 0.736 [0.732, 0.739]
Draws 40000	LOO IC 1925.4±53.19	g_p 0.316 [0.284, 0.347]	D_{xy} 0.472 [0.465, 0.478]
Chains 4	Effective p 80.64±8.57	EV 0.308 [0.249, 0.369]	
p 3	B 0.177 [0.175, 0.18]	v 2.663 [1.882, 3.532]	
		vp 0.077 [0.062, 0.092]	

	**Mode β**	**Mean β**	**Median β**	**S.E.**	**Lower**	**Upper**	**Pr(β>0)**	**Symmetry**
outcome_saq_pl_pre	0.0942	0.0949	0.0949	0.0129	0.0697	0.1201	1.0000	1.02
outcome_saq_pl_pre'	-0.0358	-0.0362	-0.0362	0.0138	-0.0635	-0.0098	0.0039	0.99
Treatment=PCI	0.8872	0.8925	0.8918	0.2088	0.4923	1.3062	1.0000	1.00

The following parameters remained separate (where not orthogonalized) during model fitting so that prior distributions could be focused explicitly on them: Treatment=PCI

Supplementary Figure S29: Coefficient density plots: SAQ physical limitation

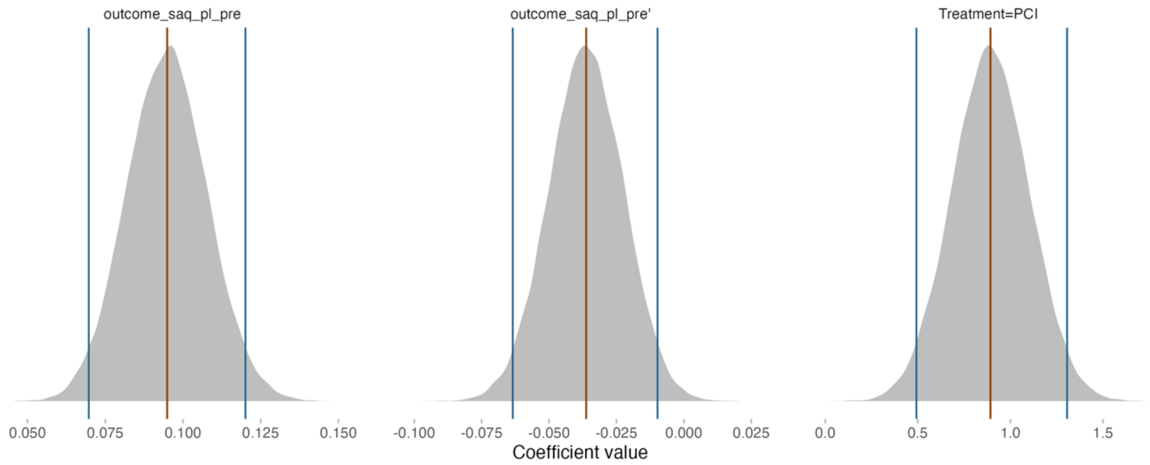

The red line is the posterior median value and the blue lines are the posterior 5th and 95th quantiles.

Supplementary Figure S30: Chain plot of Markov chain Monte Carlo draws for SAQ physical limitation

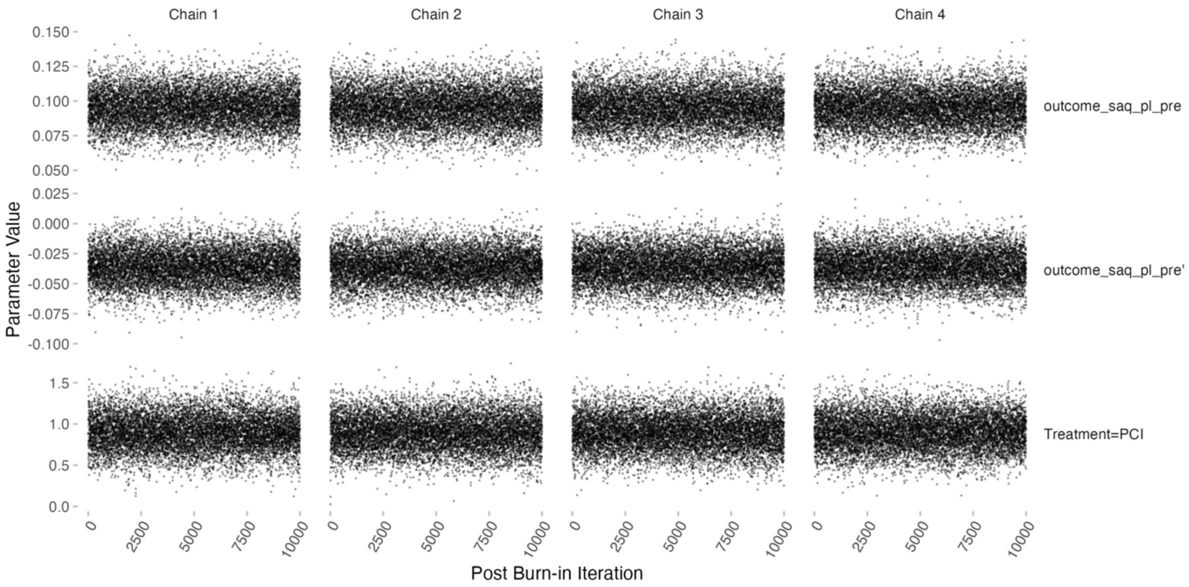

Secondary End Points - Seattle Angina Questionnaire (SAQ) angina stability

Supplementary Figure S31: Regression model and coefficients for SAQ angina stability

Bayesian Proportional Odds Ordinal Logistic Model Dirichlet Priors With Concentration Parameter 0.392 for Intercepts

```
blrm(formula = outcome_saq_stab_post ~ rcs(outcome_saq_stab_pre,
    3) + Treatment, keepsep = "^Treatment=PCI$", data = saq_stab_res1_d,
    priorsd = c(rep(100, 2), sap_treatment_sd_prior), iter = 20000,
    chains = 4, refresh = TRUE, file = "./output/saq_stab_res1.blrm.rds")
```

	Mixed Calibration/ Discrimination Indexes	Discrimination Indexes	Rank Discrim. Indexes
Obs 290	LOO log L -388.57±12.24	g 0.318 [0.127, 0.529]	C 0.559 [0.534, 0.578]
Draws 40000	LOO IC 777.14±24.48	g_p 0.041 [0.013, 0.07]	D_{xy} 0.119 [0.067, 0.157]
Chains 4	Effective p 7.75±0.56	EV 0.012 [0, 0.028]	
p 3	B 0.123 [0.119, 0.128]	v 0.094 [0.007, 0.214]	
		vp 0.002 [0, 0.004]	

	Mode β	Mean β	Median β	S.E.	Lower	Upper	Pr(β>0)	Symmetry
y≥25	2.6933	2.7085	2.7021	0.4239	1.8579	3.5176	1.0000	1.04
y≥50	1.3656	1.3653	1.3633	0.3660	0.6475	2.0810	1.0000	1.01
y≥75	-1.0571	-1.0636	-1.0617	0.3616	-1.7777	-0.3630	0.0012	0.98
y≥100	-1.8918	-1.9058	-1.9036	0.3710	-2.6303	-1.1781	0.0000	0.98
outcome_saq_stab_pre	0.0036	0.0037	0.0037	0.0090	-0.0138	0.0213	0.6612	1.00
outcome_saq_stab_pre'	0.0004	0.0003	0.0003	0.0104	-0.0201	0.0207	0.5117	1.00
Treatment=PCI	0.4410	0.4424	0.4421	0.2164	0.0172	0.8656	0.9803	1.01

The following parameters remained separate (where not orthogonalized) during model fitting so that prior distributions could be focused explicitly on them: Treatment=PCI

Supplementary Figure S32: Coefficient density plots: SAQ angina stability

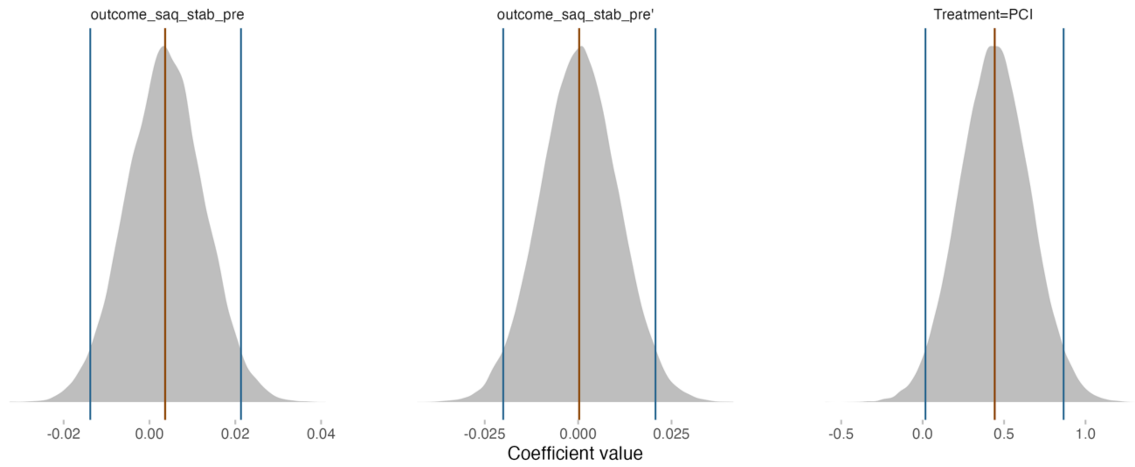

The red line is the posterior median value and the blue lines are the posterior 5th and 95th quantiles.

Supplementary Figure S33: Chain plot of Markov chain Monte Carlo draws for SAQ angina stability

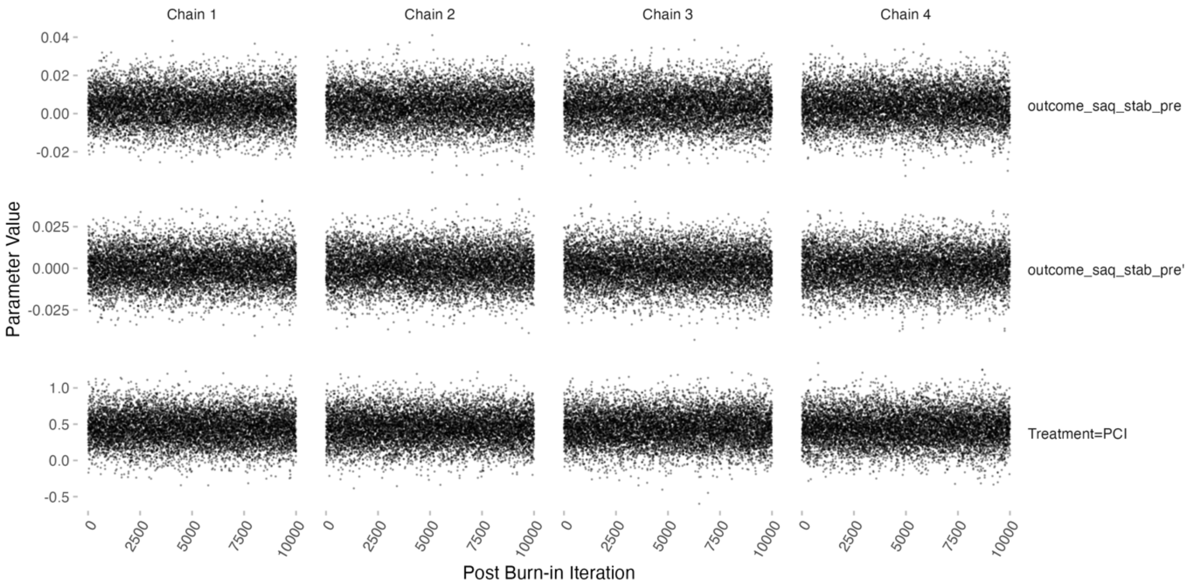

Secondary End Points - Seattle Angina Questionnaire (SAQ) quality of life

Supplementary Figure S34: Regression model and coefficients for SAQ quality of life

Bayesian Proportional Odds Ordinal Logistic Model Dirichlet Priors With Concentration Parameter 0.175 for Intercepts

```
blrm(formula = outcome_saq_qol_post ~ rcs(outcome_saq_qol_pre,
    3) + Treatment, keepsep = "^Treatment=PCI$", data = saq_qol_res1_d,
    priorsd = c(rep(100, 2), sap_treatment_sd_prior), iter = 20000,
    chains = 4, refresh = TRUE, file = "./output/saq_qol_res1.blrm.rds")
```

	Mixed Calibration/ Discrimination Indexes	Discrimination Indexes	Rank Discrim. Indexes
Obs 290	LOO log L -679.03±11.9	g 1.293 [1.022, 1.54]	C 0.712 [0.707, 0.717]
Draws 40000	LOO IC 1358.06±23.8	g_p 0.262 [0.226, 0.298]	D_{xy} 0.423 [0.414, 0.434]
Chains 4	Effective p 17.25±1.82	EV 0.214 [0.156, 0.269]	
p 3	B 0.206 [0.204, 0.209]	v 1.343 [0.836, 1.845]	
		vp 0.053 [0.039, 0.067]	

	Mode β	Mean β	Median β	S.E.	Lower	Upper	Pr(β>0)	Symmetry
outcome_saq_qol_pre	0.0644	0.0645	0.0644	0.0135	0.0386	0.0912	1.0000	1.01
outcome_saq_qol_pre'	-0.0154	-0.0153	-0.0154	0.0152	-0.0452	0.0145	0.1562	1.00
Treatment=PCI	0.7999	0.8054	0.8048	0.2046	0.4147	1.2082	1.0000	1.02

The following parameters remained separate (where not orthogonalized) during model fitting so that prior distributions could be focused explicitly on them: Treatment=PCI

Supplementary Figure S35: Coefficient density plots: SAQ quality of life

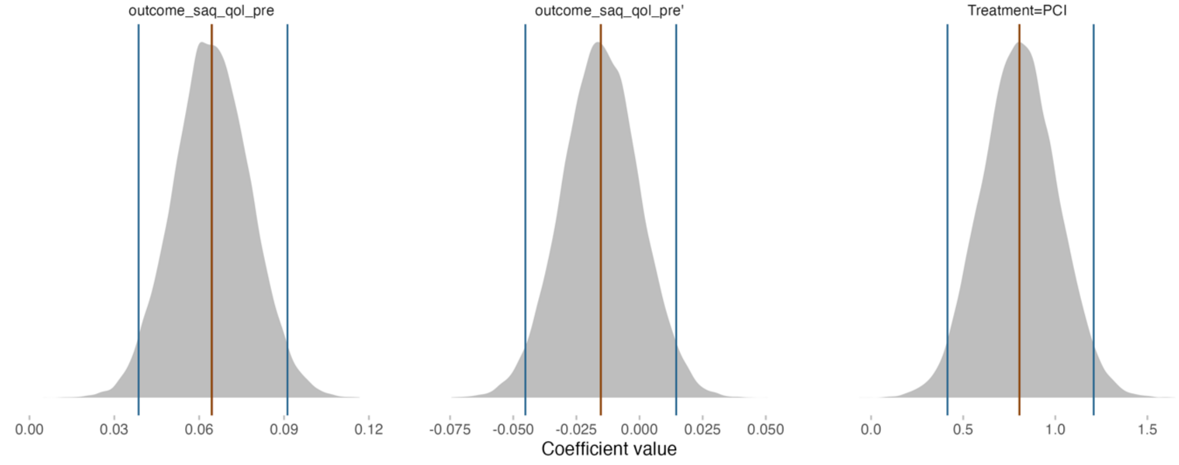

The red line is the posterior median value and the blue lines are the posterior 5th and 95th quantiles.

Supplementary Figure S36: Chain plot of Markov chain Monte Carlo draws for SAQ quality of life

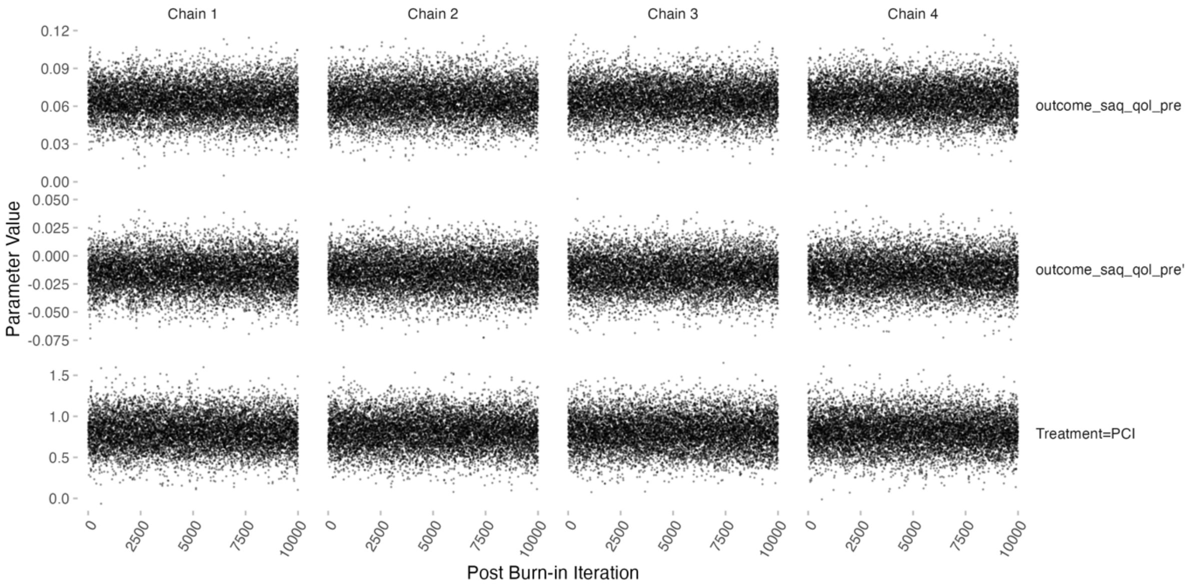

Secondary End Points - EQ-5D descriptive system

Supplementary Figure S37: Regression model and coefficients for EQ-5D descriptive system

Bayesian Proportional Odds Ordinal Logistic Model Dirichlet Priors With Concentration Parameter 0.027 for Intercepts

```
blrm(formula = eq5d_value_fu ~ rcs(eq5d_value_random, 3) + Treatment,
    keepsep = "^Treatment=PCI$", data = eq5d_res1_d, priorsd = c(rep(100,
        2), sap_treatment_sd_prior), iter = 20000, chains = 4,
    refresh = 200, file = "./output/eq5d_res1.blrm.rds")
```

	Mixed Calibration/ Discrimination Indexes	Discrimination Indexes	Rank Discrim. Indexes
Obs 289	LOO log L -1209.62±40.52	g 1.476 [1.186, 1.771]	C 0.72 [0.713, 0.725]
Draws 40000	LOO IC 2419.24±81.03	g_p 0.275 [0.237, 0.31]	D_{xy} 0.44 [0.427, 0.449]
Chains 4	Effective p 199.65±15.54	EV 0.232 [0.173, 0.29]	
p 3	B 0.204 [0.201, 0.208]	v 1.868 [1.232, 2.63]	
		vp 0.058 [0.043, 0.073]	

	Mode β	Mean β	Median β	S.E.	Lower	Upper	Pr(β>0)	Symmetry
eq5d_value_random	4.8045	4.8761	4.8680	0.8764	3.1429	6.5880	1.0000	1.03
eq5d_value_random'	2.0482	2.0209	2.0224	1.2584	-0.4816	4.4384	0.9478	1.02
Treatment=PCI	0.7727	0.7748	0.7732	0.2028	0.3840	1.1787	1.0000	1.01

The following parameters remained separate (where not orthogonalized) during model fitting so that prior distributions could be focused explicitly on them: Treatment=PCI

Supplementary Figure S38: Coefficient density plots: EQ-5D descriptive system.

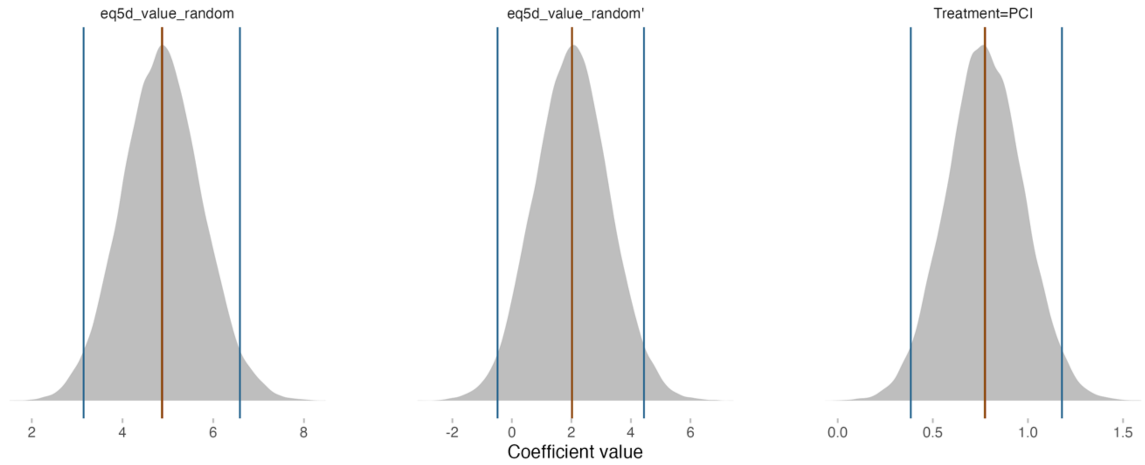

The red line is the posterior median value and the blue lines are the posterior 5th and 95th quantiles.

Supplementary Figure S39: Chain plot of Markov chain Monte Carlo draws for EQ-5D descriptive system

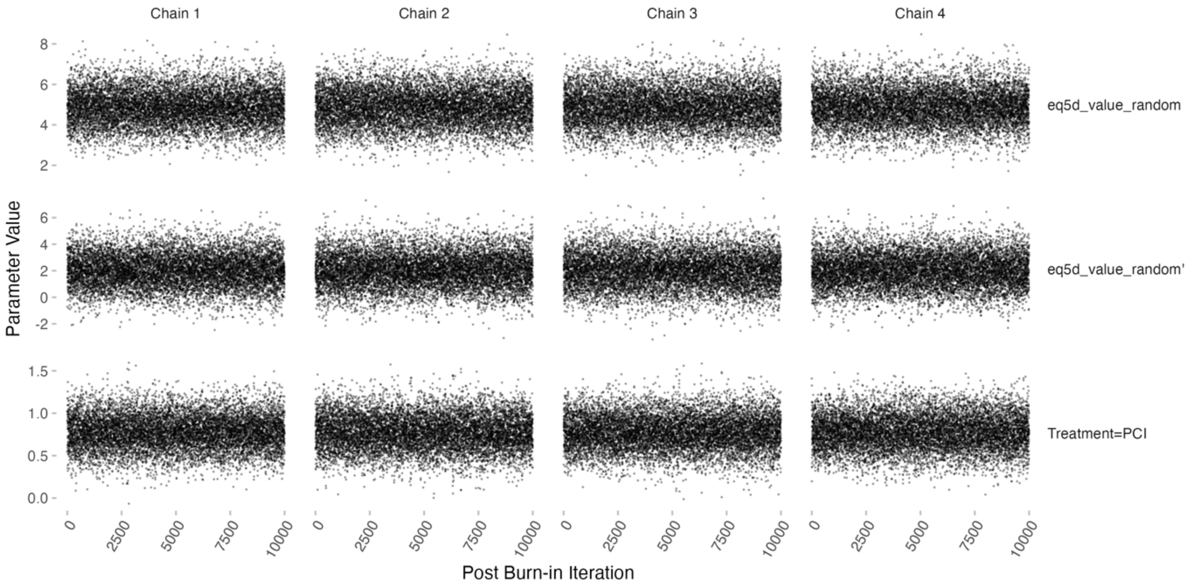

Secondary Endpoints - EQ-VAS

Supplementary Figure S40: Regression model and coefficients for EQ-VAS

Bayesian Proportional Odds Ordinal Logistic Model Dirichlet Priors With Concentration Parameter 0.081 for Intercepts

```
blrm(formula = eq5d_qol_post ~ rcs(eq5d_qol_pre, 3) + Treatment,
    keepsep = "^Treatment=PCI$", data = eq5d_qol_res1_d, priorsd = c(rep(100,
        2), sap_treatment_sd_prior), iter = 20000, chains = 4,
    refresh = TRUE, file = "./output/eq5d_qol_res1.blrm.rds")
```

Frequencies of Responses

```
  5   6  15  20  25  30  35  38  40  45  50  55  56  60  63  64  65  70  73  74  75  78  80  82  85  86
  1   1   1   2   5   5   4   1  11   7  23   6   1  22   1   1  15  28   1   1  23   1  34   2  31   2
 87  88  90  95  98  99 100
  1   2  34  14   2   4   2
```

	Mixed Calibration/ Discrimination Indexes	Discrimination Indexes	Rank Discrim. Indexes
Obs 289	LOO log L -820.2±21.41	g 1.311 [1.032, 1.572]	C 0.711 [0.705, 0.713]
Draws 40000	LOO IC 1640.4±42.82	g_p 0.264 [0.227, 0.303]	D_{xy} 0.421 [0.411, 0.426]
Chains 4	Effective p 48.51±6.59	EV 0.213 [0.149, 0.269]	
p 3	B 0.198 [0.196, 0.201]	v 1.384 [0.841, 1.961]	
		vp 0.053 [0.037, 0.067]	

	Mode β	Mean β	Median β	S.E.	Lower	Upper	Pr(β>0)	Symmetry
eq5d_qol_pre	0.0496	0.0496	0.0496	0.0115	0.0272	0.0724	1.0000	1.01
eq5d_qol_pre'	0.0226	0.0227	0.0226	0.0165	-0.0097	0.0547	0.9163	1.02
Treatment=PCI	0.6468	0.6482	0.6475	0.2010	0.2574	1.0442	0.9994	1.01

The following parameters remained separate (where not orthogonalized) during model fitting so that prior distributions could be focused explicitly on them: Treatment=PCI

Supplementary Figure S41: Coefficient density plots: EQ-5D descriptive system

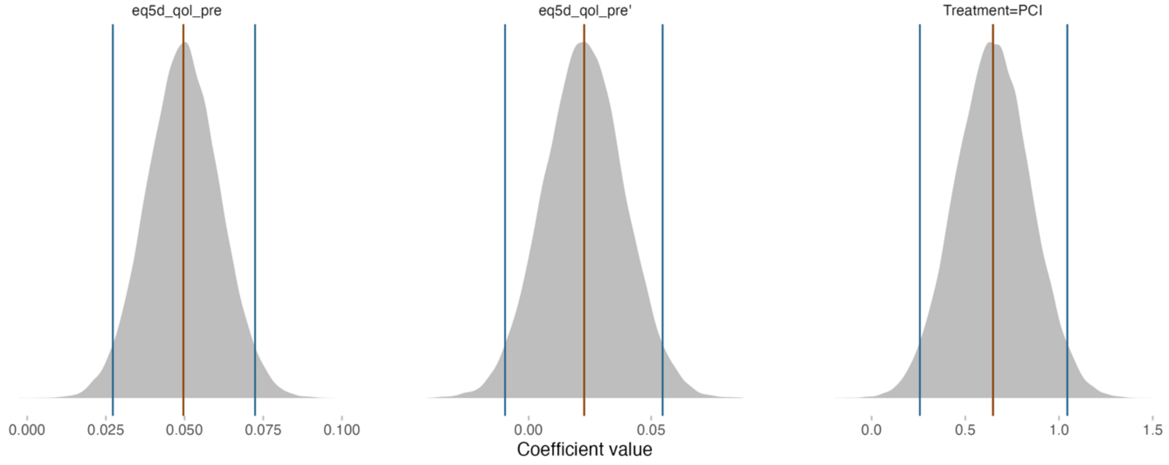

The red line is the posterior median value and the blue lines are the posterior 5th and 95th quantiles.

Supplementary Figure S42: Chain plot of Markov chain Monte Carlo draws for EQ-5D descriptive system

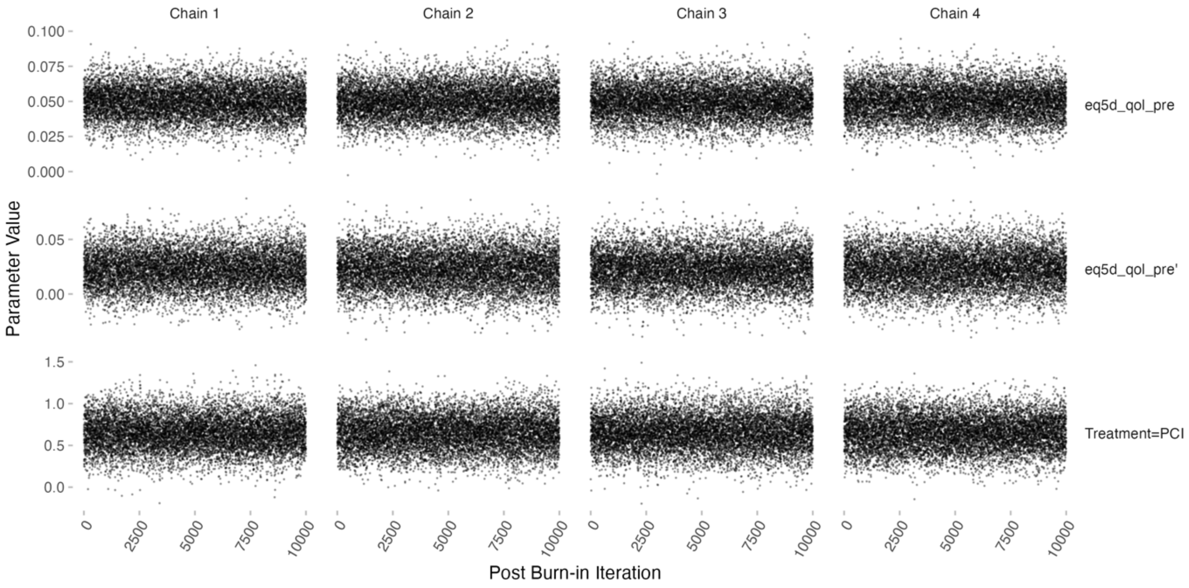

Secondary End Points - Stress echocardiography score

Supplementary Figure S43: Regression model and coefficients for stress echocardiography score

Bayesian Proportional Odds Ordinal Logistic Model Dirichlet Priors With Concentration Parameter 0.063 for Intercepts

```
blrm(formula = orbita_dse_score_fu ~ rcs(orbita_dse_score_rand,
    3) + Treatment, keepsep = "^Treatment=PCI$", data = dse_res1_d,
    priorsd = c(rep(100, 2), sap_treatment_sd_prior, rep(100,
        2)), iter = 20000, chains = 4, refresh = 100, file = "./output/dse_res1.blrm.rds")
```

	Mixed Calibration/ Discrimination Indexes	Discrimination Indexes	Rank Discrim. Indexes
Obs 230	LOO log L -745.17±22.65	g 1.084 [0.835, 1.383]	C 0.671 [0.663, 0.676]
Draws 40000	LOO IC 1490.34±45.3	g_p 0.223 [0.18, 0.267]	D_{xy} 0.341 [0.325, 0.352]
Chains 4	Effective p 64.84±7.49	EV 0.158 [0.1, 0.218]	
p 3	B 0.226 [0.22, 0.233]	v 0.975 [0.542, 1.48]	
		vp 0.039 [0.024, 0.053]	

	Mode β	Mean β	Median β	S.E.	Lower	Upper	Pr(β>0)	Symmetry
orbita_dse_score_rand	0.5851	0.5792	0.5790	0.1940	0.1909	0.9519	0.9986	1.01
orbita_dse_score_rand'	-0.5795	-0.5548	-0.5580	0.4559	-1.4576	0.3316	0.1127	1.03
Treatment=PCI	-1.1843	-1.1823	-1.1802	0.2358	-1.6443	-0.7225	0.0000	0.98

The following parameters remained separate (where not orthogonalized) during model fitting so that prior distributions could be focused explicitly on them: Treatment=PCI

Supplementary Figure S44: Coefficient density plots: stress echocardiography score

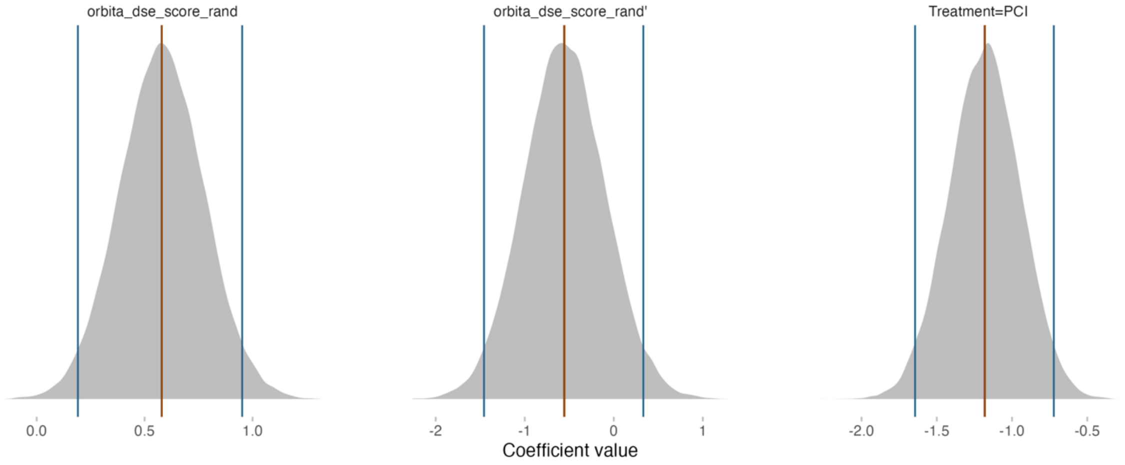

The red line is the posterior median value and the blue lines are the posterior 5th and 95th quantiles.

Supplementary Figure S45: Chain plot of Markov chain Monte Carlo draws for stress echocardiography score

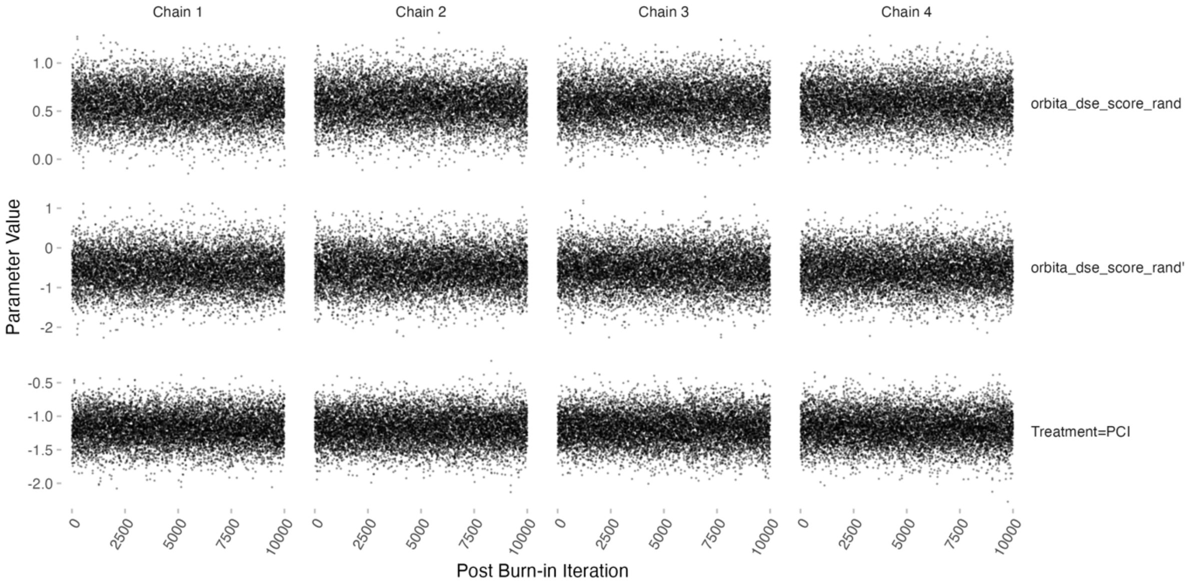

Supplementary Tables

Supplementary Table S1: Trial sites

Centre	Principal Investigator	Coinvestigators	Support team	Patients enrolled
Hammersmith Hospital (Imperial College Healthcare NHS Trust)	Dr Rasha Al-Lamee	Professor Darrel Francis<br>Dr Sayan Sen<br>Dr Sukhjinder Nijjer<br>Dr Punit Ramrakha<br>Dr Raffi Kaprielian<br>Dr Iqbal Malik<br>Dr Amarjit Sethi<br>Dr Masood Khan<br>Dr Ramzi Khamis<br>Dr Rodney Foale<br>Dr Christopher Rajkumar<br>Dr Michael Foley<br>Dr Fiyyaz Ahmed-Jushuf<br>Dr Henry Seligman	Denise Rouse<br>Hawa Amadu	163
Essex Cardiothoracic Centre (Mid and South Essex NHS Foundation Trust)	Dr Thomas Keeble	Dr John Davies<br>Dr Gerald Clesham<br>Dr Reto Gamma<br>Dr Jason Dungu<br>Dr Kare Tang<br>Dr Shah Modh Nazri<br>Dr Alamgir Kabir	Raiji Koothoor<br>Michael Galinato<br>Craig Robertson<br>Joanne Turton<br>Ellie Gudde<br>Joanne Hall<br>Karen Lyons	70
Royal Bournemouth Hospital (University Hospitals of Dorset NHS Foundation Trust)	Dr Peter O'Kane	Dr Jehangir Din<br>Dr Jonathan Hinton	Stephanie Horler<br>Annette Fraine<br>Tanith Changuion	55
Queen Alexandra Hospital (Portsmouth Hospitals University NHS Trust)	Dr Peter Haworth	-	Charlotte Turner	32
St George's Hospital (St George's University Hospitals NHS Foundation Trust)	Professor James Spratt	Dr Rupert Williams<br>Dr Claudia Cosgrove<br>Dr Pitt Lim	Stavroula Kazagli<br>Giovanna Bonato	25
Worcestershire Royal Hospital (Worcestershire Acute Hospitals NHS Trust)	Dr Helen Routledge	Dr Lal Mughal<br>Dr Jasper Trevelyan	Angela Doughty	23
Royal Free Hospital (Royal Free London NHS Foundation Trust)	Dr Tushar Kotecha	-	Nina Arnold<br>Felicity Picton<br>Tarik Mustafa<br>Leoni Bryan<br>Alejandra Perez Rodriguez<br>Valene Cadden	21
Southampton General Hospital (University Hospital Southampton NHS Foundation Trust)	Professor Nick Curzen	Dr James Wilkinson<br>Dr Alison Calver<br>Dr Rohit Sirohi<br>Dr John Rawlins<br>Dr Richard Jabbour	Karen Banks<br>Zoe Nicholas	15
Royal Berkshire Hospital (Royal Berkshire NHS Foundation Trust)	Associate Professor Neil Ruparelia	-	Mark Brunton	11
Salisbury District Hospital (Salisbury NHS Foundation Trust)	Dr Manas Sinha	-	Fiona Trim	10

University Hospital of Wales (Cardiff and Vale University Health Board)	Professor Tim Kinnaird	-	Elizabeth Hodges Elizabeth Thompson	7
Wycombe Hospital (Royal Berkshire NHS Foundation Trust)	Dr Ricardo Petraco	-	Mari Kononen Josephine Chaplin	6
Birmingham City Hospital (Sandwell and West Birmingham Hospitals NHS Trust)	Dr Fairoz Abdul	-	Sibet Joseph	1
Harefield Hospital (Royal Brompton and Harefield NHS Foundation Trust)	Dr Vasileios Panoulas	-	-	0

Supplementary Table S2: DITTO blinding framework for the catheterization laboratory

Domain	Placebo optimization strategy in ORBITA-2 Trial
**Sensory Manipulation**	Patients received incremental doses of intravenous opiate and benzodiazepine to achieve a deep level of conscious sedation such that the patient was unresponsive to verbal or tactile stimulus, with maintained airway, ventilation and cardiovascular function. Physiological support with oxygenation and intravenous fluids was administered as necessary. Additional steps for sensory manipulation are detailed below.
Visual Masking	Positioning of the patient meant that the operator screen was not visible to them.
Verbal cues	Patients were not able to hear any verbal cues due to sedation and auditory isolation. Treatment allocation was communicated from the research team to the operator away from the patient to prevent inadvertent leakage of information. During placebo procedures, catheter laboratory staff mimicked language used during PCI procedures.
Auditory cues	Auditory isolation and sedation minimised any possible auditory difference between PCI and placebo procedures.
Physical cues	Before the procedure began, patients were counselled that they may experience some pain or shortness of breath during the procedure.
Visual cues	Although subjects were sedated, the operator screen was also positioned so that it was not visible to the patient.
Auditory masking	Patients wore over-the-ear headphones playing music throughout the invasive procedure to provide auditory isolation. These were worn prior to sedation and randomization to prevent the patient hearing any communication between the clinical team.
Olfactory cues	No olfactory differences occurred between the treatment groups.
**Use of devices to optimise blinding**	In both the PCI and placebo groups, the catheterisation laboratory table and equipment table were set up for PCI. All patients underwent angiography and pressure studies as part of the randomization procedure; therefore, patients all underwent vascular access using devices which did not differ between treatment groups.
Mimicked Timings	The invasive procedure consisted of angiography and pre-randomization coronary physiological assessments. This meant that the procedure was significantly longer than a standard diagnostic coronary angiogram. Patients subsequently randomized to placebo remained on the catheter laboratory table for a minimum of 15 additional minutes following randomization to mimic the time required for PCI. Benzodiazepines utilised for sedated had a secondary effect of amnesia regarding the procedural duration.
**Restricting interaction between blinded and unblinded personnel**	The blinded ward staff managed all patients as if they had undergone PCI for post procedural monitoring and care. The catheter laboratory staff involved in the procedure were not

	permitted any contact or communication with the patient after handover.
**Omission of intervention details in trial paperwork**	The unblinded fellow entered the treatment allocation to a pre-allocated page of the online case reporting form to which none of the other members of the research team had access.<br><br>A blinded fellow performed all the communication with the patient after discharge and performed all the follow-up tests. At the 12-week point the blinded fellow contacted the unblinded fellow to confirm that all the assessments had been performed, only at that time did the unblinded fellow communicate the treatment allocation. From that time, the patient, the research team, and the clinical team became unblinded.
Intervention not specified in patient notes	A standardised protocol was used for the management of all documentation in the catheter laboratory in all centres. During the procedure, the nurses documented that the patient had participated in the ORBITA-2 trial. They did not document treatment allocation or any details of PCI in the medical notes. After the procedure, the handover between the catheter laboratory staff and ward nursing staff was carefully managed to include only location of access sites and medication given (which was identical for the two randomised arms, as all patients required heparin for physiological assessment and all patients received sedation). The handover did not indicate the treatment allocation and therefore did not indicate whether a PCI was performed. Additionally, during the handover process patients continued to have auditory isolation with music via headphones.<br><br>The unblinded fellow prepared a standardised discharge letter at the end of the procedure which informed the reader that a blinded procedure had taken place, that this procedure was a coronary angiogram +/- PCI, that all medications should remain unchanged, including continuation of dual antiplatelet therapy, until trial follow-up was complete. The letter stated that they should receive standard post-angioplasty care until full details of the procedure were provided after unblinding at 12 weeks. This standardised letter was approved by the local ethics committee and was given to all patients and their general practitioners on discharge.
Patient billing delayed or withheld	Not applicable in National Health Service (NHS) of United Kingdom
**Unblinded operator delivering component of intervention**	The unblinded operator who performed the procedure was not permitted to attend to the patient after completion of the interventional procedure. This meant that the unblinded operator was not able to review or have any communication with the patient in recovery. Furthermore, the unblinded operator was not permitted to have any contact with the patient during the 12-week blinded follow-up period, until the patient had completed the trial and been unblinded to treatment allocation.

Supplementary Table S3: Antianginal medication quantification

Common antianginal medications were classified as 1 unit based on the following total daily dosages:

Medication	Total daily dose in mg that constitutes 1 unit
Bisoprolol	5
Atenolol	25
Amlodipine	2.5
Nifedipine	20
Isosorbide mononitrate MR	30
Isosorbide mononitrate SR	25
Diltiazem	120
Nicorandil	20
Ranolazine	750
Ivabradine	5

All antianginal medication changes, including cases when it was clinically necessary to prescribe an alternate medication to the above list, were adjudicated by the Data Safety Monitoring Board.

Supplementary Table S4: Derivation of the ordinal scale primary endpoint

Grade	Number of angina episodes in a day	Units of antianginal medication	Unblinding due to intolerable angina	Acute coronary syndrome	Death
0	0	0	No	No	No
1	1	0	No	No	No
2	2	0	No	No	No
3	3	0	No	No	No
4	4	0	No	No	No
5	5	0	No	No	No
6	6 or more	0	No	No	No
7	0	1	No	No	No
8	1	1	No	No	No
9	2	1	No	No	No
10	3	1	No	No	No
11	4	1	No	No	No
12	5	1	No	No	No
13	6 or more	1	No	No	No
14	0	2	No	No	No
15	1	2	No	No	No
16	2	2	No	No	No
17	3	2	No	No	No
18	4	2	No	No	No
19	5	2	No	No	No
20	6 or more	2	No	No	No
21	0	3	No	No	No
22	1	3	No	No	No
23	2	3	No	No	No
24	3	3	No	No	No
25	4	3	No	No	No
26	5	3	No	No	No
27	6 or more	3	No	No	No
28	0	4	No	No	No
29	1	4	No	No	No

30	2	4	No	No	No
31	3	4	No	No	No
32	4	4	No	No	No
33	5	4	No	No	No
34	6 or more	4	No	No	No
35	0	5	No	No	No
36	1	5	No	No	No
37	2	5	No	No	No
38	3	5	No	No	No
39	4	5	No	No	No
40	5	5	No	No	No
41	6 or more	5	No	No	No
42	0	6	No	No	No
43	1	6	No	No	No
44	2	6	No	No	No
45	3	6	No	No	No
46	4	6	No	No	No
47	5	6	No	No	No
48	6 or more	6	No	No	No
49	0	7	No	No	No
50	1	7	No	No	No
51	2	7	No	No	No
52	3	7	No	No	No
53	4	7	No	No	No
54	5	7	No	No	No
55	6 or more	7	No	No	No
56	0	8	No	No	No
57	1	8	No	No	No
58	2	8	No	No	No
59	3	8	No	No	No
60	4	8	No	No	No
61	5	8	No	No	No
62	6 or more	8	No	No	No
63	0	9	No	No	No
64	1	9	No	No	No

65	2	9	No	No	No
66	3	9	No	No	No
67	4	9	No	No	No
68	5	9	No	No	No
69	6 or more	9	No	No	No
70	0	10	No	No	No
71	1	10	No	No	No
72	2	10	No	No	No
73	3	10	No	No	No
74	4	10	No	No	No
75	5	10	No	No	No
76	6 or more	10	No	No	No
77	N/A	N/A	Yes	No	No
78	N/A	N/A	N/A	Yes	No
79	N/A	N/A	N/A	N/A	Yes

Supplementary Table S5: Representativeness of Study Participants

Category	
Disease, problem, or condition under investigation	Stable coronary artery disease (CAD)
Special considerations related to:	
Sex and gender	Obstructive CAD affects more men than women.[5]
Age	The prevalence of CAD increases with age.[6]
Race or ethnic group	Asian individuals carry a higher risk of CAD than White individuals.[7] Black individuals have an equivalent risk of having fatal CAD as White individuals; however, they have a lower risk of suffering from non-fatal CAD.[8]
Geography	CAD is a worldwide healthcare concern with approximately 620 million patients affected globally.[9] CAD prevalence varies among regions in the world. Eastern Europe has the highest age-standardized prevalence, followed by Central Asia and Central Europe. Central sub-Saharan Africa, southern Latin America and high-income Asia Pacific have the lowest age-standardized prevalence.[10]
Other considerations	Socioeconomic status has also been shown to play a role in the development of CAD. Lower social classes are at an increased risk of developing CAD.[11]
Overall representativeness of this trial	The ORBITA-2 trial recruited a high proportion of male participants (79%). Female patients were relatively underrepresented, similar to previous trials of coronary intervention (12% in BCIS-REVIVED[12], 22% in FAME2[13], 15% in COURAGE[14]). Only biological sex was reported.<br><br>Most patients were White (76%). 23% of participants were Asian and 1% were Black. These proportions are broadly consistent with the population of England and Wales (81.7% White, 9.3% Asian and 2.5% Black in the UK 2021 Census), with a relative under-representation of Black patients and over-representation of Asian patients. This likely reflects the ethnicities of the populations served by the clinical trial sites. The smartphone application was only available in the English language. Where required, translation was provided. However, this may limit the generalizability of the smartphone application to a non-English speaking population.

Supplementary Table S6: Blood results for randomized patients

	**PCI**<br>**n=151**	**Placebo**<br>**n=150**
Hemoglobin (g/L)	143 (13)	142 (14)
HbA1c (mmol/mol)	45 (12)	44 (11)
Creatinine (μmol/L)	83 (21)	82 (25)
Triglycerides (mmol/L)	1.45 (1.01)	1.37 (0.80)
Total cholesterol (mmol/L)	3.95 (1.42)	3.81 (0.96)
HDL cholesterol (mmol/L)	1.19 (0.35)	1.21 (0.40)
LDL cholesterol (mmol/L)	1.99 (0.98)	2.02 (0.67)

Data are presented as mean (SD).

Supplementary Table S7: Post-PCI coronary physiology

	PCI (N=151)	Placebo (N=150)
Post-PCI FFR		
Mean	0.89 (0.07)	-
Median (IQR)	0.89 (0.85-0.93)	-
No. vessels assessed — no./total no.	161/193	-
Post-PCI iFR		
Mean	0.94 (0.05)	-
Median (IQR)	0.93 (0.91-0.97)	-
No. vessels assessed — no./total no.	168/193	-

Data are presented as mean (SD) and no. (%) unless otherwise stated.
PCI denotes percutaneous coronary intervention, FFR fractional flow reserve, iFR instantaneous wave-free ratio.
*Where iFR was not available, an alternative non-hyperemic pressure ratio was utilized.

Supplementary Table S8: Bayesian analysis of primary and secondary end points.

**Primary Endpoint**			
	**Odds ratio of transitioning to a better clinical state each day with PCI vs placebo**		**Probability of benefit with PCI vs placebo**
Angina symptom score<br>Follow-up (Day 84)<br>Follow-up (Day 2)	OR 1.88, 95% CrI 1.57 to 2.27<br>OR 1.54, 95% CrI 1.32 to 1.81		>99.9%<br>>99.9%
**Components of primary endpoint**			
Daily angina episodes<br>Follow-up (Day 84)<br>Follow-up (Day 2)	OR 1.93, 95% CrI 1.58 to 2.33<br>OR 1.51, 95% CrI 1.29 to 1.78		>99.9%<br>>99.9%
Daily antianginal medication units<br>Follow-up (Day 84)<br>Follow-up (Day 2)	OR 1.09, 95% CrI 0.46 to 2.62<br>OR 1.43, 95% CrI 0.72 to 2.94		57.5%<br>84.0%
**Secondary Endpoints**			
	**PCI**	**Placebo**	
**Treadmill exercise time (seconds)**			
n	123	112	
Baseline mean	619		
Follow-up	694<br>(658 to 729)	642<br>(603 to 680)	
Increment	76<br>(39 to 111)	23<br>(-16 to 62)	
Benefit of PCI over placebo	52<br>(12 to 92)		
**Canadian Cardiovascular Society class**			
n	147	146	
Baseline median	2		
Follow-up	0·94<br>(0·77 to 1.11)	1.65<br>(1·47 to 1·85)	
Increment	-1·06<br>(-1.23 to -0.89)	-0·34<br>(-0·53 to -0·15)	
Benefit of PCI over placebo	-0·72<br>(-0·95 to -0·49)		

**SAQ angina frequency**			
n	146	145	
Baseline median	60.0		
Follow-up	79.6 (75.5 to 83.6)	65.1 (60.3 to 69.8)	
Increment	19.6 (15.5 to 23.6)	5.1 (0.3 to 9.8)	
Benefit of PCI over placebo	14.6 (9.5 to 19.5)		
**SAQ physical limitation**			
n	139	144	
Baseline median	66.7		
Follow-up	82.7 (79.1 to 86.1)	73.7 (69.8 to 77.4)	
Increment	16.0 (12.4 to 19.5)	7.0 (3.1 to 10.7)	
Benefit of PCI over placebo	8.9 (4.9 to 12.9)		
**SAQ angina stability**			
n	145	145	
Baseline median	50.0		
Follow-up	61.4 (56.9 to 66.0)	55.4 (50.5 to 60.1)	
Increment	11.4 (6.9 to 16.0)	5.4 (0.5 to 10.1)	
Benefit of PCI over placebo	6.0 (0.3 to 11.6)		
**SAQ quality of life**			
n	145	145	
Baseline median	41.7		
Follow-up	61.9 (57.5 to 66.1)	51.9 (47.6 to 56.2)	
Increment	20.2 (15.8 to 24.4)	10.3 (6.0 to 14.5)	
Benefit of PCI over placebo	9.9 (5.2 to 14.8)		

**EQ-5D descriptive system**			
n	145	144	
Baseline median	0·75		
Follow-up	0·80 (0·77 to 0·83)	0·73 (0·70 to 0·76)	
Increment	0·05 (0·03 to 0·08)	-0·02 (-0·05 to 0·01)	
Benefit of PCI over placebo	0·07 (0·04 to 0·11)		
**EQ-VAS**			
n	146	143	
Baseline median	70.0		
Follow-up	72.9 (69.8 to 75.8)	66.8 (63.2 to 70.1)	
Increment	2.9 (-0.2 to 5.8)	-3.2 (-6.8 to 0.1)	
Benefit of PCI over placebo	6.1 (2.3 to 9.8)		
**Stress echocardiography score**			
n	119	111	
Baseline mean	1·81		
Follow-up	0.96 (0·72 to 1·22)	1·84 (1·45 to 2.27)	
Increment	-0·86 (-1.09 to -0.60)	0.03 (-0.37 to 0.45)	
Benefit of PCI over placebo	-0·88 (-1.26 to -0.52)		

PCI denotes percutaneous coronary intervention, SAQ denotes Seattle Angina Questionnaire, EQ-5D denotes EuroQOL 5 dimensions, and EQ-VAS denotes EuroQOL visual analogue scale. Treadmill exercise time and stress echocardiography score are presented for the patients who had both pre-randomization and follow-up scores.

The Canadian Cardiovascular Society class ranges from 0 to IV where class 0 denotes no angina and class IV denotes angina at rest. SAQ scores range from 0 to 100, with higher scores indicating better health status. On the European Quality of Life–5

Dimensions (EQ-5D) descriptive system values range from 0-1, and on the EQ-VAS values range from 0 to 100, with higher scores indicating better health status. The method for derivation of the stress echocardiography score has been previously published.[15]

*Calculated as SAQ angina frequency of 100.

Supplementary Table S9: Sensitivity analysis for priors on treatment effect.

Primary Endpoint		
	**Odds ratio of transitioning to a better clinical state each day with PCI vs placebo**	**Probability of benefit with PCI vs placebo**
**Diffuse prior**		
Angina symptom score		
Follow-up (Day 84)	OR 1.86, 95% CrI 1.55 to 2.25	>99.9%
Follow-up (Day 2)	OR 1.54, 95% CrI 1.31 to 1.81	>99.9%

A sensitivity analysis testing the effect of replacing the sceptical prior on the treatment effect with an essentially flat diffuse prior.

Supplementary Table S10: Antianginal medication use.

	Antianginal units prescribed	
	PCI	Placebo
**Pre-enrollment**		
Mean<br>Median (range)	2.23<br>2 (0 to 8)	2.31<br>2 (0 to 10)
**Pre-randomization**		
Mean<br>Median (range)	0.47<br>0 (0 to 7)	0.57<br>0 (0 to 9)
**Follow-up**		
Mean<br>Median (range)	0.46<br>0 (0 to 7)	0.61<br>0 (0 to 7)

Supplementary Table S8 describes the quantity of prescribed antianginal medication units stratified by timepoint of the trial and randomization group. Antianginal dosing is expressed in antianginal equivalent units for integration into the angina symptom score primary endpoint. Dose conversion into standardized units for common antianginal medications is described in Supplementary Table S3.

Supplementary Table S11: Sensitivity analysis with multiple imputation for missing data.

	PCI	Placebo	Difference (95% CI)	P Value
Treadmill exercise time — seconds	702.8	644.3	58.4 (15.3 to 101.5)	-
Stress echocardiography score	0.80	1.88	-1.09 (-1.48 to -0.70)	-

For missing data for the treadmill exercise time and stress echocardiography score outcomes, multiple imputation was performed using bootstrapping and predictive mean matching. This method fits a flexible additive regression model to a bootstrapped sample of the original data and uses this model to predict all of the original missing and non-missing values for the target variable, and then for each missing value, uses predictive mean matching to identify the value among the non-missing values that has the closest predictive value. The method for derivation of the stress echocardiography score has been previously published.[15]

Supplementary Table S12: Serious adverse events contributing to primary end point.

All serious adverse events and cross over events were reviewed and adjudicated by the independent DSMB.

Event	Arm	Event	Details
1	Placebo	Unblinding due to intolerable angina	Chest pain at rest 20 days following randomization to placebo arm. No ECG changes. No biomarker elevation. Further episode of chest pain at rest in hospital. Decision taken to unblind.
2	Placebo	Acute coronary syndrome	Chest pain at rest 1 day following randomization to placebo arm. Initial troponin elevated >ULN. Patient unblinded and underwent PCI.
3	Placebo	Acute coronary syndrome	At 12-week follow-up dobutamine stress echocardiography, sonographer identified extensive LAD territory infarct on resting images. This was not present on pre-randomization imaging. On direct questioning patient reported a severe episode of chest pain 2 weeks prior but did not see medical attention. Subsequently underwent PCI. Adjudicated as missed type 1 MI.
4	Placebo	Acute coronary syndrome	Fifteen days following randomization to placebo, experienced mild chest pain at rest worsening on minimal exertion. No ECG changes but troponin rise > ULN. Patient unblinded and underwent revascularization.
5	Placebo	Acute coronary syndrome	Episode of chest pain at rest 42 days following randomization to placebo. Found to have normal ECG but troponin > ULN. Unblinded and underwent PCI.
6	Placebo	Acute coronary syndrome	Chest pain on minimal exertion 30 days following randomization to placebo procedure. Dynamic ECG changes and troponin > ULN. Unblinded and underwent revascularization.
7	Placebo	Acute coronary syndrome	Episode of chest pain at rest. Ischaemic ECG on admission to emergency department with resting hypokinesia on echocardiography. Decision made to unblind. Underwent urgent revascularization. Subsequent troponin > ULN.
8	PCI	Acute coronary syndrome	Following randomization to PCI, no re-flow developed following initial predilatation with cutting balloon. ST-elevation with patient agitation requiring intubation. Multiple intracoronary adenosine boluses. PCI completed with drug eluting stent implantation. Persisting ST-elevation at end of case with

			troponin > ULN. Remained inpatient for 48 hours. Unblinded. Uneventful recovery.
9	PCI	Acute coronary syndrome	Randomized to PCI for severe in-stent restenosis of LAD-Diagonal bifurcation stent. Loss of flow to diagonal branch following pre-dilation of LAD with cutting balloon. ST elevation and patient agitation. Unable to pass back into diagonal branch despite extensive efforts. Accepted loss of branch vessel. Troponin > ULN. Patient unblinded and remained inpatient for 3 days prior to discharge.
10	PCI	Acute coronary syndrome	Following randomization to PCI, LAD predilated with cutting balloon. Loss of flow noted to two diagonal branches. Culotte bifurcation stenting performed to restore flow to larger vessel. Accepted loss of flow to smaller branch. Patient remained inpatient for rhythm monitoring. Chest pain, ECG changes and troponin > ULN. Remained blinded and completed trial.
11	PCI	Acute coronary syndrome	Following randomization to PCI, patient underwent rotablation and intravascular lithotripsy to heavily calcified LAD. 2 x drug luting stents deployed. LAD perforation during post-dilation of the proximal stent. Managed with balloon tamponade and placement of 3 x covered stents. Troponin > ULN. Unblinded with uneventful recovery.

Supplementary Table S13: Serious adverse events not contributing primary end point.

Event	Arm	Event	Details
12	PCI	Stroke	Diplopia reported by patient immediately after randomization procedure. Transferred to stroke unit and underwent thrombolysis. Remained blinded and completed study.
13	Placebo	GI bleed	Admitted to hospital with per-rectal bleeding. Small arterial blush noted in distal sigmoid colon. Managed conservatively with blood products and tranexamic acid. DAPT withheld for 1 week. Remained blinded and completed study.
14	Placebo	Delirium	New onset delirium 54 days following randomization to placebo. No focal neurology. Normal CT Head. Resolved spontaneously. Remained blinded and completed study.
15	PCI	Coronary dissection	Noticed after randomization to PCI. Proceeded to PCI.

		during pressure wire	
16	PCI	Anaphylaxis	During routine follow-up dobutamine stress echocardiography patient had anaphylactic reaction on Sonovue contrast agent. Resuscitated with IM adrenaline. Remained blinded and completed study.
17	Placebo	Chest pain requiring hospital admission	Patient presented to emergency department with more severe chest pain than usual. Reviewed by blinded cardiologist. Atypical pain, with serial negative troponins. Discharged with no changes to medications. Remained blinded and completed study.
18	Placebo	Hospital admission for carcinoid syndrome	Patient with known neuroendocrine tumour admitted from clinic with fatigue, weight loss, diarrhoea, anorexia, peripheral oedema, visual symptoms. Managed by specialist team with course of steroids. Antianginal medications changed during admission. Remained blinded and completed study.
19	PCI	Coronary dissection requiring additional PCI	Following randomization to PCI, target vessel (LAD) predilated and intravascular lithotripsy performed. Catheter associated dissection of LMS subsequently noted and successfully treated with DK crush of LMS-LAD-LCx. Discharged same day. Remained blinded and completed study.
20	Placebo	Femoral bleed	Femoral approach for randomization procedure (severe radial spasm). Angioseal deployed at end of case and patient discharged. Readmitted 5 days later with large femoral bleed and pseudoaneurysm. 2 x thrombin injections performed with successful resolution. Remained blinded and completed study.
21	Placebo	Femoral bleed	Femoral approach for randomization procedure (failed radial). Micropuncture kit utilized. Despite this, large femoral haematoma. Managed conservatively. Admitted overnight for monitoring. Remained blinded and completed study.
22	Placebo	Radial artery dissection	Dissection of right radial artery with large haematoma. Switched to left radial artery and pressure cuff applied to right arm. Stayed in hospital overnight for observation. Remained blinded and completed study.
23	Placebo	Epistaxis requiring	Severe epistaxis requiring admission to hospital for nose packing. Continued dual

		hospital admission	antiplatelet therapy. Remained blinded and completed study.
24	PCI	Stroke	Patient presented to hospital 1 day after randomization to PCI with left sided foot drop and abnormality of his gait. MRI brain showed bilateral small infarcts. Remained on dual antiplatelet therapy and subsequently discharged Remained blinded and completed study.
25	Placebo	Hospital admission	Admission to hospital with lymphadenopathy and night sweats. Full diagnostic work up ongoing. Remained blinded
26	Placebo	Bell's Palsy	Woke with left sided facial droop 10 days following randomization to placebo. MRI excluded acute stroke. Diagnosed with Bell's palsy. Treated with course of steroids. Remained blinded and completed study
27	PCI	Loss of diagonal branch during PCI	Following randomization to PCI, loss of flow to small diagonal branch noted after deploying stent to LAD. No clinical sequelae. Patient discharged same day. Remained blinded and completed study.

Supplementary Table S14: Failure to deliver randomized therapy.

All were analysed on an intention-to-treat principle.

**Event**	**Arm**	**Event**	**Details**
28	Placebo	Pressure wire dissection requiring crossover to PCI	Very severe LAD stenosis, difficulty crossing lesion with pressure wire. Microcatheter exchange required. Following randomization to placebo and wire removal, flow appeared reduced in target vessel, therefore cross over to PCI. Remained blinded and completed trial.
29	Placebo	Pressure wire dissection requiring crossover to PCI	Very severe proximal LAD stenosis. Following randomization to placebo and withdrawal of pressure wire, patient developed ST elevation. Concern regarding plaque instability, therefore crossed over to PCI. Remained blinded and completed trial.
30	PCI	Diffuse disease crossover to placebo	Following randomization to PCI, optical coherence tomography (OCT) showed extremely diffuse disease not appreciated on angiography alone. Operator decision that PCI would not be appropriate based on pattern of disease. Crossed over to placebo. Remained blinded and completed trial.

31	PCI	Failure to complete PCI	Complex RCA and LAD disease. Following randomization to PCI, RCA treated but with high contrast and radiation dose. Operator decision that treatment of LAD could not be performed during same sitting. Patient unblinded and withdrawn from trial.

References

1. Nowbar AN, Rajkumar C, Foley M, et al. A double-blind randomised placebo-controlled trial of percutaneous coronary intervention for the relief of stable angina without antianginal medications: design and rationale of the ORBITA-2 trial. EuroIntervention 2022;17:1490–7.

2. Ganesananthan S, Rajkumar CA, Foley M, Francis D, Al-Lamee R. Remote digital smart device follow-up in prospective clinical trials: early insights from ORBITA-2, ORBITA-COSMIC, and ORBITA-STAR. Eur Heart J Suppl 2022;24:H32–42.

3. Bang H, Ni L, Davis CE. Assessment of blinding in clinical trials. Controlled Clinical Trials 2004;25:143–56.

4. Al-Lamee R, Howard JP, Shun-Shin MJ, et al. Fractional Flow Reserve and Instantaneous Wave-Free Ratio as Predictors of the Placebo-Controlled Response to Percutaneous Coronary Intervention in Stable Single-Vessel Coronary Artery Disease. Circulation 2018;138:1780–92.

5. Steg PG, Greenlaw N, Tardif J-C, et al. Women and men with stable coronary artery disease have similar clinical outcomes: insights from the international prospective CLARIFY registry. Eur Heart J 2012;33:2831–40.

6. Kannel WB, Feinleib M. Natural history of angina pectoris in the Framingham study. Prognosis and survival. Am J Cardiol 1972;29:154–63.

7. Zaman MJS, Philipson P, Chen R, et al. South Asians and coronary disease: is there discordance between effects on incidence and prognosis? Heart 2013;99:729–36.

8. Colantonio LD, Gamboa CM, Richman JS, et al. Black-White Differences in Incident Fatal, Nonfatal, and Total Coronary Heart Disease. Circulation 2017;136:152–66.

9. British Heart Foundation UK Factsheet [Internet]. 2023;Available from: https://www.bhf.org.uk/-/media/files/for-professionals/research/heart-statistics/bhf-cvd-statistics-uk-factsheet.pdf

10. Roth GA, Johnson C, Abajobir A, et al. Global, Regional, and National Burden of Cardiovascular Diseases for 10 Causes, 1990 to 2015. J Am Coll Cardiol 2017;70:1–25.

11. Schultz WM, Kelli HM, Lisko JC, et al. Socioeconomic Status and Cardiovascular Outcomes: Challenges and Interventions. Circulation 2018;137:2166–78.

12. Perera D, Clayton T, O'Kane PD, et al. Percutaneous Revascularization for Ischemic Left Ventricular Dysfunction. N Engl J Med 2022;387:1351–60.

13. De Bruyne B, Pijls NHJ, Kalesan B, et al. Fractional flow reserve-guided PCI versus medical therapy in stable coronary disease. N Engl J Med 2012;367:991–1001.

14. Boden WE, O'Rourke RA, Teo KK, et al. Optimal medical therapy with or without PCI for stable coronary disease. N Engl J Med 2007;356:1503–16.

15. Al-Lamee RK, Shun-Shin MJ, Howard JP, et al. Dobutamine Stress Echocardiography Ischemia as a Predictor of the Placebo-Controlled Efficacy of Percutaneous Coronary Intervention in Stable Coronary Artery Disease: The Stress Echocardiography-Stratified Analysis of ORBITA. Circulation 2019;140:1971–80.
